# Genome-Wide Analysis of Terpene Synthase Gene Family in *Mentha longifolia* and Catalytic Activity Analysis of a Single Terpene Synthase

**DOI:** 10.3390/genes12040518

**Published:** 2021-04-02

**Authors:** Zequn Chen, Kelly J. Vining, Xiwu Qi, Xu Yu, Ying Zheng, Zhiqi Liu, Hailing Fang, Li Li, Yang Bai, Chengyuan Liang, Weilin Li, Bernd Markus Lange

**Affiliations:** 1 Institute of Botany, Jiangsu Province and Chinese Academy of Sciences (Nanjing Botanical Garden Mem. Sun Yat-Sen), Nanjing 210014, China; chenzq1219@cnbg.net (Z.C.); xiwuqi@cnbg.net (X.Q.); yuxu84@163.com (X.Y.); fanghailing2013@163.com (H.F.); xinwenbanlili@163.com (L.L.); baiyang.89@163.com (Y.B.); 2 Department of Horticulture, Oregon State University, Corvallis, OR 97331, USA; kelly.vining@oregonstate.edu; 3 Zhejiang Provincial Key Laboratory of Resources Protection and Innovation of Traditional Chinese Medicine, Hangzhou 311300, China; alice.zhengy@gmail.com; 4 Nanjing Foreign Language School, Nanjing 210008, China; lzq030729@gmail.com; 5 Jiangsu Key Laboratory for the Research and Utilization of Plant Resources, Nanjing 210014, China; 6 College of Forest, Nanjing Forestry University, Nanjing 210037, China; wlli@njfu.edu.cn; 7 Institute of Biological Chemistry and M.J. Murdock Metabolomics Laboratory, Washington State University, Pullman, WA 99164, USA; lange-m@wsu.edu

**Keywords:** *Mentha longifolia*, terpene synthase, terpenoids, limonene synthase

## Abstract

Terpenoids are a wide variety of natural products and terpene synthase (TPS) plays a key role in the biosynthesis of terpenoids. *Mentha* plants are rich in essential oils, whose main components are terpenoids, and their biosynthetic pathways have been basically elucidated. However, there is a lack of systematic identification and study of TPS in *Mentha* plants. In this work, we genome-widely identified and analyzed the *TPS* gene family in *Mentha longifolia*, a model plant for functional genomic research in the genus *Mentha*. A total of 63 *TPS* genes were identified in the *M. longifolia* genome sequence assembly, which could be divided into six subfamilies. The *TPS-b* subfamily had the largest number of genes, which might be related to the abundant monoterpenoids in *Mentha* plants. The *TPS-e* subfamily had 18 members and showed a significant species-specific expansion compared with other sequenced Lamiaceae plant species. The 63 *TPS* genes could be mapped to nine scaffolds of the *M. longifolia* genome sequence assembly and the distribution of these genes is uneven. Tandem duplicates and fragment duplicates contributed greatly to the increase in the number of *TPS* genes in *M. longifolia*. The conserved motifs (RR(X)8W, NSE/DTE, RXR, and DDXXD) were analyzed in *M. longifolia* TPSs, and significant differentiation was found between different subfamilies. Adaptive evolution analysis showed that *M. longifolia TPSs* were subjected to purifying selection after the species-specific expansion, and some amino acid residues under positive selection were identified. Furthermore, we also cloned and analyzed the catalytic activity of a single terpene synthase, *MlongTPS29*, which belongs to the *TPS-b* subfamily. *MlongTPS29* could encode a limonene synthase and catalyze the biosynthesis of limonene, an important precursor of essential oils from the genus *Mentha*. This study provides useful information for the biosynthesis of terpenoids in the genus *Mentha*.

## 1. Introduction

Terpenoids are the largest and a structurally diverse group of natural products in plants [1]. To date, more than 80,000 terpenoid compounds, including monoterpenes, sesquiterpenes, and diterpenes, have been identified [2,3]. Terpenoids play important roles in both primary and secondary metabolism of plants. For example, gibberellin, brassinosteroid, and carotenoid are well characterized terpenoids, which play important roles in plant growth and development as plant hormones and photosynthetic pigments [4]. Compared to the small amount of terpenoids involved in primary metabolism, the majority of terpenoids are classified as secondary metabolites. Although they are not involved in the basic growth and development of plants, they still have some physiological functions and a wide range of applications, including plant defense response, pharmacological compounds, and fragrance and aroma constituents [5,6,7].

Although the number of terpenoids is huge, they are all derived biosynthetically from common precursors, dimethylallyl diphosphate (DMAPP) and isopentenyl diphosphate (IPP) [8]. These precursors are produced by two biosynthetic pathways, the methylerythritol phosphate pathway (MEP) in the chloroplast and the mevalonate pathway (MVA) in the cytosol [9]. The condensation reaction of DMAPP and IPP catalyzed by prenyltransferases produces the direct precursors geranyl diphosphate (GPP C10), farnesyl diphosphate (FPP C15), and geranylgeranyl pyrophosphate (GGPP C20). Subsequently, terpene synthases (TPSs) catalyze the precursors to form a variety of terpenoids, including hemiterpene (C5), monoterpene (C10), sesquiterpene (C15), and diterpene (C20) [10,11]. The products of TPS can be further modified by other enzymatic reaction, such as dehydrogenation, isomerization, and group transfer. In the biosynthetic pathway of terpenoids, TPSs is positioned at the branch point and is a key enzyme for terpenoid biosynthesis.

Each full-length TPS is characterized by two conserved domains with Pfam ID PF01397 (N-terminal) and PF03936 (C-terminal) [1]. The N-terminal domain has a conserved RRX8W motif, and the C-terminal domain has a conserved DDXXD motif and NSE/DTE motif [12]. *TPSs* constitute a mid-size gene family, the number of which varies greatly in different plants [12]. To date, *TPS* gene families have been genome-widely identified in various plant species, ranging from spermatophytes to mosses [13]. According to the phylogenetic analysis, the plant *TPS* family can be classified into seven subfamilies (TPS-a, TPS-b, TPS-c, TPS-d, TPS-e/f, TPS-g, and TPS-h) [12,13]. Different subfamily genes also encode terpene synthase with different functions, for example, TPS-a subfamily genes encode sesquiterpene synthases, while TPS-b and TPS-g subfamily genes encode monoterpene synthases [14]. TPS-d is a gymnosperm-specific subfamily, which performs several functions, such as diterpene, monoterpene, and sesquiterpene synthases [15]. The *TPS* genes could also been classified into different classes according to their genomic structure, including class I (13-15 exons), class II (10 exons), and class III (7 exons) [16].

The genus *Mentha* encompasses mint species cultivated for their essential oils, which are widely used in the flavor, fragrance, and aromatherapy industries [17]. The major constituents of mint essential oils are monoterpenes, including (−)-menthol, (+)-neomenthol, (+)-isomenthol, (+)-carvone, and (+)-menthofuran [18,19]. The biosynthetic pathway of the most abundant oil constituents has been well illustrated in peppermint (*Mentha* × *piperita* L.) and spearmint (*Mentha spicata* L.) [20,21]. Limited by the complex polyploidy, the genome research of peppermint and spearmint has been progressing slowly. The horse mint (*Mentha longifolia*) is an ancestor species of the genus *Mentha*, which has been developed as a model species for mint genomics because of its diploid genome structure, relatively small genome, and other genetics features [22]. The genome sequencing of *M. longifolia* has been completed and updated to a pseudochromosome level of quality, which provides good opportunities for genome-wide analysis of terpenoid biosynthesis in the genus *Mentha* [23].

Considering the importance of terpenoid compounds in *M. longifolia* and the limited knowledge of their biosynthesis, genome-wide identification of *TPS* genes was conducted in this study. Then, sequence features, gene family classification, genome localization, and phylogenetic analyses were performed to characterize the *TPS* family. Furthermore, a candidate *TPS* gene encoding a limonene synthase was cloned, and the catalytic activity was also assayed.

## 2. Materials and Methods

### 2.1. Data Retrieval and Identification of TPSs

The proteome data of the sequenced Labiatae plants were downloaded from http://www.ndctcm.org/shujukujieshao/2015-04-23/27.html (*Salvia miltiorrhiza*) [24], http://caps.ncbs.res.in/Ote/ (*Ocimum tenuiflorum*) [25], http://ocri-genomics.org/Sinbase/ (*Sesamum indicum*) [26], and http://gigadb.org/dataset/100463 (*Salvia splendens*) [27] (Accessed data: 21 July 2020). For the identification of TPSs, the TPS specific Pfam N-terminal domain model (PF01397) and C-terminal domain model (PF03936) were downloaded from the Pfam website (http://pfam.xfam.org/) [28]. Then, an HMM search (v3.1b2) [29] was conducted to search the proteome using the PF01397 and PF03936 domain model data as queries. Candidate genes with both N-terminal and C-terminal domains were considered as complete TPSs and used for further analysis. The *Arabidopsis* TPS sequences were downloaded from TAIR (https://www.arabidopsis.org/) (Accessed data: 21 July 2020). The genome data of *M. longifolia* were downloaded from Mint Genomics Resource (http://langelabtools.wsu.edu/mgr/) (Accessed data: 5 May 2020). The assembly of the *M. longifolia* genome contains 12 large scaffolds encompassing 462.6 Mb, which is consistent with the previously reported genome size (400~500 Mb) [22]. The new assembly corresponds to at least 92.5% of the predicted genome size. Due to the lack of gene prediction of the *M. longifolia* genome sequence assembly, a BLAT-based method was used to identify TPSs in *M. longifolia* genome sequence assembly [30]. The protein query set representing the TPS family used for BLAT was constructed based on the PF01397 and PF03936 seed sequences. The target sequences and flanking sequences in the *M. longifolia* genome sequence were extracted and then imported to Genscan for gene prediction [31]. The conserved N-terminal and C-terminal domains of *M. longifolia* TPSs were confirmed on the SMART website (http://smart.embl-heidelberg.de/).

### 2.2. Multiple Sequence Alignment and Phylogenetic Analyses

The multiple sequence alignment of TPSs from *M. longifolia* and other plants was performed using the MUSCLE3.6 software [32]. The alignment results were imported to MGEA X to construct the phylogenetic tree [33]. The phylogenetic tree was constructed using the maximum likelihood method with the Jones Taylor Thornton (JTT) model. The bootstrap value for the phylogenetic tree was 1000 replicates. The phylogenetic tree was further modified using iTOL (https://itol.embl.de/) [34].

### 2.3. Characterization of TPSs from M. longifolia

The gene structure of TPSs from *M. longifolia* was determined based on annotation information and then illustrated using Exon-Intron Graphic Maker (http://www.wormweb.org/exonintron). Subcellular localization of *M. longifolia* TPSs was predicted using the AtSubP tool (http://bioinfo3.noble.org/AtSubP/index.php) and ProtComp (http://linux1.softberry.com/berry.phtml?topic=protcomppl&group=programs&subgroup=proloc). The location of *M. longifolia TPS* genes on the scaffold was determined by Tbtools [35]. Tandemly duplicated genes were identified by their sequence similarity and scaffold localization according to earlier studies [36,37]. The conserved motifs of *M. longifolia* TPSs, including the RR(X)8W motif, NSE/DTE motif, RXR motif, and DDXXD motif, were identified based on the multiple sequence alignment results.

### 2.4. Adaptive Evolution Analysis of M. longifolia TPSs

Based on the phylogenetic tree and duplication gene analysis of the *M. longifolia TPS* gene family, 14 paralog pairs were selected to calculate the nonsynonymous-to-synonymous substitution ratio (Ka/Ks). The calculation was conducted using a KaKs-Calculator 2.0 [38] with the sliding window method (90 bp window and 30 bp slide). Then, the site model of EasyCodeML [39] was used to conduct adaptive evolution analyses on each subfamily of *M. longifolia TPSs*. Three pairs of models (M0 (one-ratio) vs. M3 (discrete), M1a (neutral) vs. M2a (positive selection), and M7 (β) vs. M8 (β + ω)) were chosen to test positive selection using the likelihood ratio test (LRT) and the Bayes empirical Bayes (BEB) method [40,41].

### 2.5. RNA Isolation and MlongTPS29 Cloning

The *M. longifolia* used to extract RNA was introduced from the Botanical Garden Berlin-Dahlem in Germany with the accession number of ES-0-B-0180887 and then cultivated at the Germplasm Nursery in the Institute of Botany, Jiangsu Province and Chinese Academy of Sciences, Nanjing, Jiangsu Province. Total RNA of *M. longifolia* leaves was extracted using a FastPure Plant Total RNA Isolation Kit (Vazyme, Nanjing, China) according to the manufacturer’s instructions. After quality and concentration detection, 1 μg of total RNA was used to synthesize the first strand cDNA with a HiScript II 1st Strand cDNA Synthesis Kit (Vazyme, Nanjing, China). To identify the candidate limonene synthase in *M. longifolia* genome sequence, limonene synthases of *M. spicata* (AAC37366.1) and *M. piperita* (ABW86881.1) were used as queries to BLAST in *M. longifolia* TPSs. Polymerase chain reaction (PCR) was performed to amplify *MlongTPS29* with a gene-specific forward primer (5′-ATGGCTTTCAAAGTGTTTAGTG-3′) and reverse primer (5′-TCATGCAAAGGGCTCGAAT-3′). The amplified fragments were purified using the TaKaRa MiniBEST Agarose Gel DNA Extraction Kit Ver.4.0 (Takara, Dalian, China) and then cloned into the pClone007 Blunt Simple Vector (Tsingke, Beijing, China). The positive clones were screened and sequenced for confirmation.

### 2.6. Expression of Recombinant MlongTPS29 in Escherichia coli and Enzyme Assays

The coding sequence of *MlongTPS29* was cloned into the prokaryotic expression vector pET28a using the homologous recombination method. Briefly, *MlongTPS29* was amplified with primers containing homology arms. The forward primer was 5′-*CAAATGGGTCGCGGATCC*ATGGCTTTCAAAGTGTTTAGTG-3′, and the reverse primer was 5′-*GGCCGCAAGCTTGTCGAC*TCATGCAAAGGGCTCGAAT-3′ (Italic indicates homology arms). The pET28a vector was digested with the restriction endonuclease *Bam*HI and *Sal*I. Then, the homologous recombination was performed with a Trelief™ SoSoo Cloning Kit Ver.2 (Tsingke, Beijing, China) according to the manufacturer’s instructions. The recombinant vector was transformed into *E. coli* BL21 (DE3), and the expression of recombinant MlongTPS29 was induced by addition of isopropyl-β-D-thiogalactoside (IPTG) to a final concentration of 1 mM. After cultured at 16 °C for 20 h, the cells were collected by centrifugation and washed twice using reaction buffer (50 mM HEPES, pH 7.5, with 5 mM MgCl_2_, 2 mM MnCl_2_, 200 mM KCl, 5 mM dithiothreitol, and 10% (*v*/*v*) glycerol). Then, the cells were resuspended in reaction buffer and disrupted by sonication. After centrifugation at 16,000× *g* at 4 °C for 15 min, the supernatant was collected and used for further enzyme assays.

The enzyme activity of MlongTPS29 was detected according to an earlier report with minor modification [42]. Briefly, the supernatant of *E. coli* with recombinant MlongTPS29 was added to a 200 μL reaction mixture, and then 10 μM of GPP was added to initiate the reaction. The reaction mixture was incubated at 30 °C for 1 h. Products of the reaction were extracted with dichloromethane and then detected by an Agilent 8860/5977B GC-MS system equipped with a DB-5MS column (30 m × 0.25 mm i.d.). The oven temperature was isothermal at 45 °C, then increased at a rate of 10 °C/min to 220 °C, and maintained at 220 °C for 2 min.

## 3. Results

### 3.1. Identification of TPS Genes in M. longifolia Genome Sequence

The HMM-based method and BLAST-based method are commonly used to identify the TPS gene family in plants. In this study, due to the lack of gene prediction of the *M. longifolia* genome, a BLAT-based method was used to identify TPS family. Using the conserved TPS N-terminal domain (PF01397) and C-terminal domain (PF03936) seed sequences as queries, 89 and 99 TPS-N and TPS-C genes were identified after gene model prediction, respectively. By comparing the two results, 78 candidate *TPS* genes were obtained. After confirming the conserved domains manually, we finally identified 63 TPSs containing both TPS N-terminal and TPS C-terminal domains in the *M. longifolia* genome sequence (Table 1, File S1).

### 3.2. Phylogenetic Analyses of TPSs from M. longifolia and Other Lamiaceae Plants

**Figure 1:**
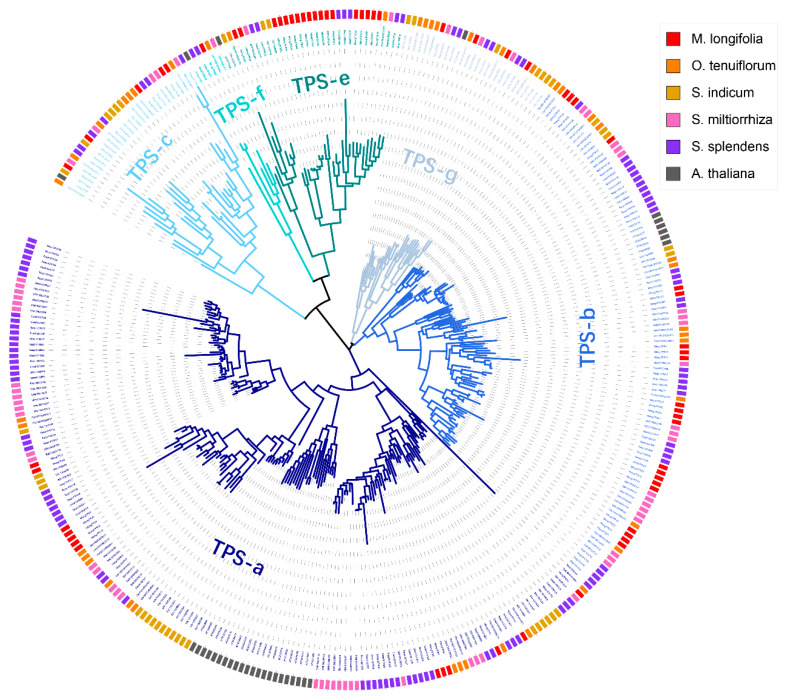
Phylogenetic analysis of TPSs in *M. longifolia*, *Arabidopsis thaliana* and other Lamiaceae plants. Species: *M. longifolia* (Mlong), *Ocimum teruiflorum* (Ote), *Sesamu indicum* (Sin), *Salvia miltiorrhiza* (Smi), *Salvia splendens* (Ssp), *A. thaliana* (Ath).

To examine the evolutionary relationships of *M. longifolia* TPSs, a phylogenetic tree was constructed using the *M. longifolia* TPSs and TPSs from *Arabidopsis thaliana* and the other four sequenced Lamiaceae plants, namely, *O. teruiflorum*, *S. indicum*, *S. miltiorrhiza*, and *S. splendens*. The phylogenetic tree demonstrated that TPS proteins were clustered into six subfamilies, including TPS-a, TPS-b, TPS-c, TPS-e, TPS-f, and TPS-g (Figure 1). No TPS-d or TPS-h gene was identified because TPS-d was gymnosperm specific, and TPS-h was only observed in *Selaginella moellendorffii* [12]. Some species-specific clades were observed, for example, 22 TPS-a subfamily genes of *A. thaliana* clustered into a clade and 11 TPS-b subfamily genes of *S. splendens* clustered into a clade. Among the Lamiaceae plants analyzed in this study, the TPS-a subfamily had the largest number of genes except for *M. longifolia*, the gene number of TPS-b subfamily of which was more than that of the TPS-a subfamily (Table 2). Comparing the gene numbers of each subfamily, it is worth noting that the gene number of the TPS-e subfamily in *M. longifolia* genome sequence assembly was much higher than that of the other Lamiaceae plants, and there was a significant species-specific expansion for the TPS-e subfamily in *M. longifolia* (Table 2).

### 3.3. Classification of M. longifolia TPSs Based on the Phylogenetic Tree

**Figure 2:**
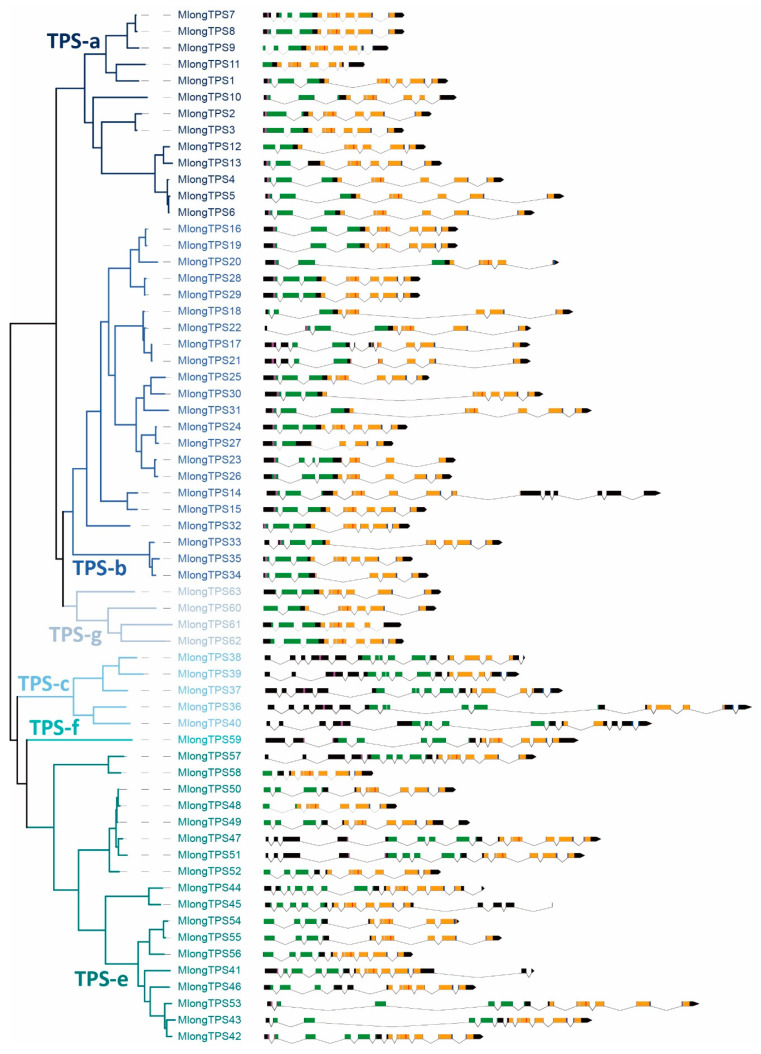
Phylogenetic analysis, subfamily classification, gene structure and conserved domains in *M. longifolia* TPSs. The black rectangles represent exons and the lines represent introns. The coding sequences of the conserved N-terminal domain, C-terminal domains, RR(X)8W motif, NSE/DTE motif, RXR motif, and DDXXD motif are represented in green, orange, purple, blue, gray, and red, respectively.

The phylogenetic analysis of 63 *M. longifolia TPSs* was performed using MEGA X with the maximum likelihood method. Based on the phylogenetic tree, 63 *M. longifolia TPSs* could be divided into 6 subfamilies, namely, 13 *TPS-a* genes, 22 *TPS-b* genes, 5 *TPS-c* genes, 18 *TPS-e* genes, 1 *TPS-f* gene, and 4 *TPS-g* genes. The *TPS-e* and *TPS-f* subfamilies were always merged into one subfamily since *TPS-f* is derived from *TPS-e*, and they were clustered into one clade (Figure 2). It is worth noting that there are 18 *TPS-e* subfamily genes in *M. longifolia* genome sequence, which is much more than that reported for most other plants [13].

### 3.4. Exon-Intron Stucture of M. longifolia TPS Genes

The numbers of exons and introns in plant *TPS* genes are relatively low. According to the intron-exon pattern, *TPS* genes can be divided into three classes, class I, class II, and class III, which contain 12-14 introns, 9 introns, and 6 introns, respectively [16]. In this study, most *TPS-a*, *TPS-b* and *TPS-g* subfamily genes of *M. longifolia* contain six to eight exons and five to seven introns (Table 1 and Figure 2), and they all belonged to class III TPSs. The *TPS-c* subfamily genes contain 14 to 15 exons and 13 to 14 introns (Table 1 and Figure 2), which belonged to class I TPSs. The gene structure of the *TPS-e* subfamily genes showed a relatively large variation. The exon numbers of *TPS-e* subfamily genes varied from 6 to 14, and part of which exhibited a loss of exons in the 5′-terminal (Table 1 and Figure 2).

### 3.5. Genomic Distribution of M. longifolia TPS Genes

**Figure 3:**
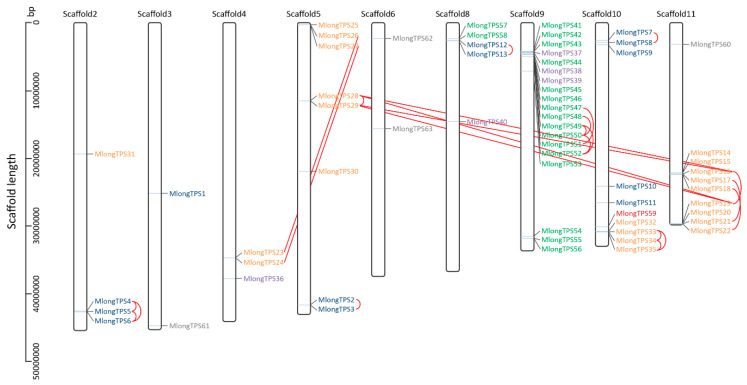
Scaffold localization of *TPS* genes in *M. longifolia* genome sequence assembly. The *M. longifolia* genome sequence assembly contains 12 large scaffolds encompassing 462.6 Mb, and the 63 *TPS* genes are mapped to nine scaffolds based on their localization information. The Y-axis represents the length of the scaffolds. *TPS* genes of *TPS-a*, *TPS-b*, *TPS-c*, *TPS-e*, *TPS-f*, and *TPS-g* subfamilies are indicated in blue, orange, purple, green, red, and gray fonts, respectively. The tandem duplication and segment duplication *TPS* genes are indicated in red lines.

The 63 *TPS* genes were mapped to nine scaffolds of *M. longifolia* genome sequence assembly based on their localization information (Figure 3). The distribution of these genes is uneven, for example, only two *TPS* genes mapped onto scaffold3 and scaffold6, while 19 *TPS* genes clustered on scaffold9. The clustered distribution of some subfamily members was also observed, such as nine *TPS-b* genes clustering on scaffold11 and 16 *TPS-e* genes clustering on scaffold9. Tandem duplication and segment duplication are common phenomena related to the increase in gene copies in plants. In this study, tandem duplication and segment duplication of *TPS* genes were also analyzed. Seven tandem duplicates and 3 segment duplicates of *TPS* genes were observed in the *M. longifolia* genome sequence assembly, and it contained a total of 30 *TPS* genes (Figure 3). The duplication events occurred in the *TPS-a*, *TPS-b*, and *TPS-e* subfamilies.

### 3.6. Conserved Motif Analyses of M. longifolia TPSs

**Figure 4:**
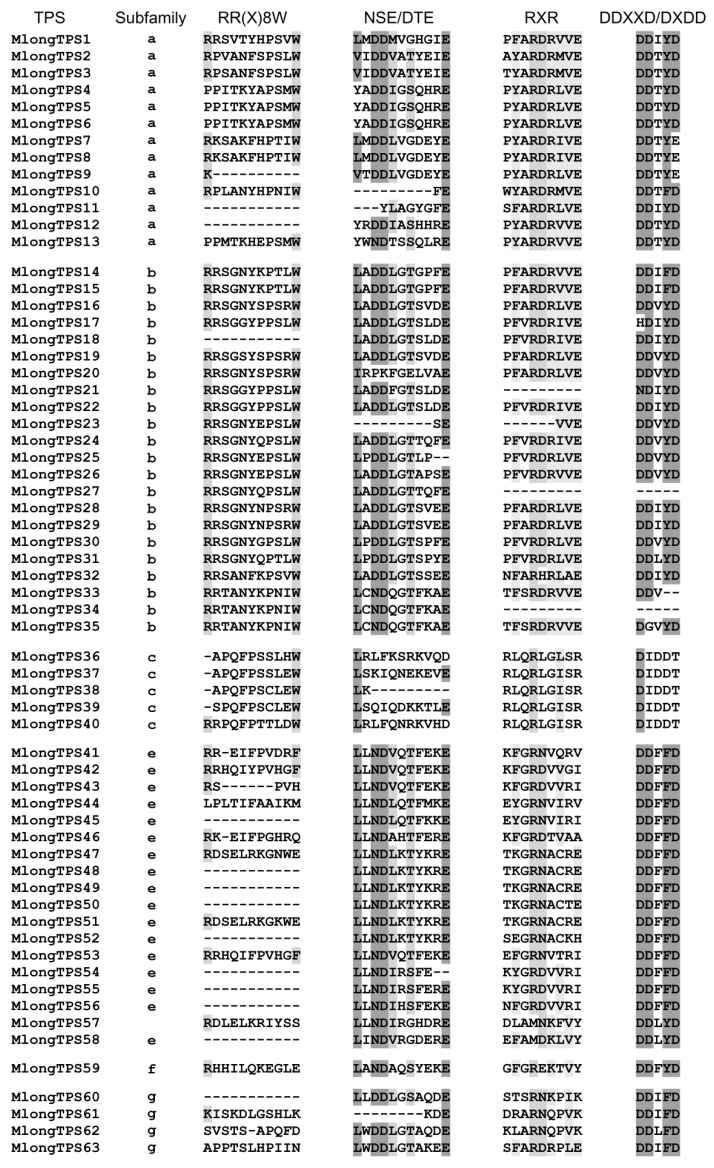
The conserved RR(X)8W, NSE/DTE, RXR, and DDXXD motifs in *M. longifolia* TPSs.

TPS harbors conserved structural features such as the RR(X)8W motif in the N-terminal domain and DDXXD and NSE/DTE motifs in the C-terminal domain, which play important roles in the catalytic function of TPS [12,43]. In our study, conserved motifs were analyzed in *M. longifolia* TPSs, and significant differentiation was found between different subfamilies (Figure 4). The RR(X)8W motif is conserved in the TPS-b subfamily and plays a role in initiation of the isomerization cyclization reaction [44]. Both the TPS-b and TPS-g subfamilies are angiosperm monoterpene synthases, but the TPS-g proteins do not contain this motif. The TPS-g proteins are required for the biosynthesis of acyclic monoterpenes, which form floral volatile organic compounds (VOCs) [45]. It has been reported that the TPS-a subfamily encodes only sesquiterpene synthase, and the second arginine of the RR(X)8W motif is not conserved [46]. The NSE/DTE motif is conserved in most subfamilies except for the TPS-c subfamily. The RXR motif is conserved in the TPS-a and TPS-b subfamilies. The DDXXD motif is the most conserved motif among these TPSs and is conserved in the TPS-a, TPS-b, TPS-e, TPS-f, TPS-g subfamilies but not the TPS-c subfamily (Figure 4). The DDXXD motif is involved in the coordination of divalent ions and water molecules and the stabilization of the active site [47,48]. The TPS-c proteins are not expected to have this domain as they do not cleave the prenyl diphosphate unit; however, they contain a DXDD motif that is critical for the protonation initiate reaction [49].

### 3.7. Adaptive Evolution Analysis of M. longifolia TPSs

**Figure 5:**
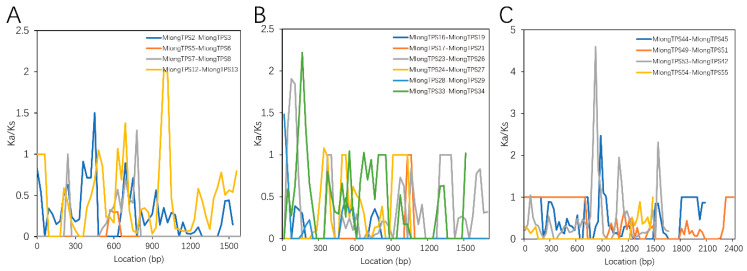
Sliding-window adaptive evolution analysis of the *M. longifolia TPS* paralog genes. (**A**–**C**) represent paralog genes of TPS-a, TPS-b, and TPS-e subfamilies, respectively.

In order to explore whether positive selection drove the evolution of the *M. longifolia TPS* gene family, the nonsynonymous-to-synonymous substitution ratio (Ka/Ks = ω) was calculated to estimate the positive selection. Using the sliding window of 90 bp and a moving step of 30 bp, the Ka/Ks ratios of 14 *M. longifolia TPS* paralog pairs were calculated (Figure 5). A few sites in eight paralog pairs (three, three, and two for the TPS-a, TPS-b, and TPS-e subfamilies, respectively) had Ka/Ks > 1, and most sites had Ka/Ks < 1, suggesting that most *M. longifolia TPS* genes were subjected to purifying selection after the species-specific expansions. To further investigate the evolutionary selection pressures acting on *M. longifolia TPS* genes, the site models of each subfamily were calculated using EasyCodeML. As shown in Table 3, all the subfamilies were subject to purification selection with ω ranging from 0.202 to 0.310. Some amino acid residues under positive selection were identified in the TPS-c and TPS-g subfamilies.

### 3.8. Enzyme Activity Assays of MlongTPS29

**Figure 6:**
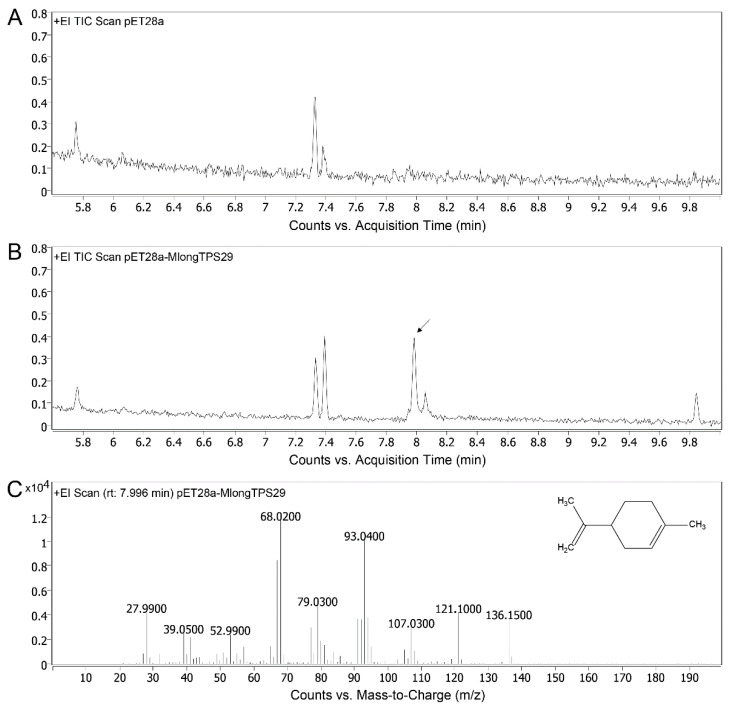
GC-MS analysis of the products formed by recombinant MlongTPS29 proteins via in vitro assays. (**A**,**B**) Total ion current of products yielded by pET28a and pET28a-MlongTPS29, respectively. (**C**) Mass spectrum of the indicated peak.

Limonene is an important precursor of the essential oil components of the genus *Mentha*, whose synthesis is catalyzed by limonene synthase (LS). In order to identify the candidate LS in *M. longifolia* genome sequence, LSs of *M. spicata* and *M. piperita* were used as queries to BLAST in *M. longifolia* TPSs. As a result, a candidate LS-coding gene, *MlongTPS29*, was identified in *M. longifolia* genome sequence. The coding sequence of *MlongTPS29* is 1800 bp, which is the same as that for the *LS* homologs in *M. spicata* and *M. piperita*. Multiple sequence alignment also showed that MlongTPS29 was considerably similar to the LS of *M. spicata* and *M. piperita* (Figure S1). Both the sequence length and sequence similarity indicate that *MlongTPS29* is complete. This gene was cloned and then subjected to assay its catalytic activity. The recombinant MlongTPS29 was heterologous expressed in *E. coli* and used to construct the reaction in vitro. After adding GPP as a substrate, GC-MS analysis showed that the limonene could be detected in the MlongTPS29 group, while no limonene was detected in the empty pET28a group (Figure 6). This result indicates that MlongTPS29 could catalyze the production of limonene from GPP.

## 4. Discussion

The genus *Mentha* has important economic value for its abundance of essential oils. The major constituents of mint essential oils are monoterpenes and sesquiterpenes [18,19]. *Mentha* plants (especially peppermint and spearmint) have been employed as model systems for the study of monoterpene biosynthesis [20,21]. However, the complex polyploidy and lack of genomic information limited further study. Horse mint (*M. longifolia*) is a diploid ancestor species of the genus *Mentha*, which has been developed as a model species for mint genomics [22]. The completion of *M. longifolia* genome sequencing provides opportunity to perform functional genomic studies of *Mentha* plants [23]. In this study, the *TPS* gene family, which is positioned at the branch point and is a key enzyme for terpenoid biosynthesis, was genome-widely identified and analyzed in *M. longifolia* genome sequence assembly. A total of 63 complete *TPS* genes were identified in the *M. longifolia* genome sequence assembly according to the conserved N-terminal and C-terminal domains of TPS. *TPS* belongs to a medium-sized gene family, with various gene numbers (approximately 20-150) among different plants [12]. The number of *TPS* genes in *M. longifolia* genome sequence assembly is moderate when compared to that of other reported plants.

According to the phylogenetic analysis, *TPSs* of *M. longifolia* fall into six known angiosperm *TPS* subfamilies (*TPS-a*, *TPS-b*, *TPS-c*, *TPS-e*, *TPS-f*, and *TPS-g*). No gymnosperm-specific *TPS-d* subfamily or *S. moellendorffii*-specific *TPS-h* subfamily genes were identified. However, recent studies indicated that the *TPS-d* subfamily is not gymnosperm-specific, it was also found in *Ananas comosus* and *Marchantia polymorpha* [13]. *TPS-b* is the largest subfamily in *M. longifolia* genome sequence, and it has more members than the *TPS-a* subfamily (34.9%*TPS-b* genes and 20.6% *TPS-a* genes). This is in contrast to most other plants, such as *A. thaliana* (18.8% *TPS-b* genes and 68.8% *TPS-a* genes) [50], *Vitis vinifera* (29.0% *TPS-b* genes and 43.5% *TPS-a* genes) [46], and *Oryza sativa* (5.0% *TPS-b* genes and 62.5% *TPS-a* genes) [13]. The genomic distribution analysis showed that there were some tandem duplicates and segment duplicates in *TPS-b* genes, which might be the cause of the increase in the number of TPS-b subfamily genes in *M. longifolia* genome sequence [13]. The *TPS-b* subfamily is mainly responsible for catalyzing the biosynthesis of monoterpenoids, and monoterpenoids are the main components of the essential oils of *Mentha* plants [1,18]. Therefore, we speculate that the expansion of the *TPS-b* subfamily of *Mentha* may be related to the rich monoterpenoid content. Another interesting phenomenon is that there are 18 *TPS-e* subfamily genes in *M. longifolia* genome sequence, which is much higher than that of most other plants. It is worth noting that most *TPS-e* genes (15 of 18) are distributed on scaffold9, and tandem duplicates also exist in this subfamily. Whether the species-specific expansion of *TPS-e* in *M. longifolia* causes functional differentiation remains unclear. The integrated chemical-genomic-phylogenetic approach in Lamiaceae revealed that gene family expansion rather than increasing the enzyme promiscuity of terpene synthase is correlated with mono- and sesquiterpene diversity [51]. GC-MS analysis showed that the diversity of mono- and sesquiterpene in the genus *Mentha* was more abundant than that in other genera of Lamiaceae [51]. The catalytic function of the expanded *TPS-e* subfamily needs further investigation.

The *TPS* genes could also been classified into different classes according to their genomic structure, including class I (13-15 exons), class II (10 exons), and class III (7 exons), which appear to have evolved sequentially from class I to class III [16]. Class I TPSs consist primarily of diterpene synthases found in gymnosperms (secondary metabolism) and angiosperms (primary metabolism). Class II TPSs evolved from class I by loss of the conifer diterpene internal sequence domain. Class III TPSs consist of angiosperm monoterpene, sesquiterpene, and diterpene synthases involved in the secondary metabolism, which evolved from Class II by loss of introns [16]. There are differences in gene structure between different subfamilies, while members of the same subfamily show minor differences. *TPS-a*, *TPS-b*, and *TPS-g* subfamilies with 6 to 8 exons belong to class III *TPS*, while *TPS-c*, *TPS-e* and *TPS-f* with 13 to 15 exons belong to class I *TPS*. In *M. longifolia* genome sequence, the gene structure of *TPS* is basically consistent with the subfamily classification, except for *TPS-e*. By comparing *TPS-e* genes with other plants, it was observed that some *M. longifolia TPS-e* genes have a loss of exons in the 5′-terminal. It has been suggested that during the evolutionary process, class I *TPS* genes will loss exons and introns successively to form a new class, so we speculate that these exon-losing *TPS* genes may be involved in this evolutionary process. Whether this exon deletion affects its function remains unclear.

The main components of essential oils of *Mentha* plants are monoterpenoids, which are mainly catalyzed by the TPS-b subfamily. In this study, we selected the *MlongTPS29*, a putative limonene synthase encoding genes belonged to the TPS-b subfamily, for catalytic activity analysis. Limonene is the most important precursor of the essential oil components of the genus *Mentha*, which is catalyzed by limonene synthase. In peppermint and spearmint (two widely cultivated *Mentha* plants), the limonene synthase has been identified and shown to catalyze the synthesis of limonene from GPP [52]. The results of our study indicate that MlongTPS29 could also catalyze the production of limonene from GPP in vitro.

## 5. Conclusions

In this study, we genome-widely identified and analyzed the *TPS* gene family in *M. longifolia* genome sequence assembly, a model plant for functional genomic research in the genus *Mentha*. A total of 63 *TPS* genes were identified in the *M. longifolia* genome sequence, which could be divided into six subfamilies. The TPS-e subfamily had 18 members and showed a significant species-specific expansion compared with other plants. The 63 *TPS* genes could be mapped to nine scaffolds of *M. longifolia* genome sequence assembly, and the tandem duplicates and fragment duplicates contributed greatly to the increase in the number of *TPS* genes. The conserved motifs of *M. longifolia* TPSs were significantly differentiated between different subfamilies. Adaptive evolution analysis showed that *M. longifolia TPSs* were subjected to purifying selection after the species-specific expansion, and some amino acid residues under positive selection were identified. We also cloned a *TPS-b* gene, *MlongTPS29*, which could encode a limonene synthase and catalyze the biosynthesis of limonene, an important precursor of essential oils from the genus *Mentha*. This study provides useful information for the biosynthesis of terpenoids in the genus *Mentha*.

## Figures and Tables

**Table 1:** Statistics of *TPS* gene information of *Mentha longifolia*.

Gene ID	Scaffold	Start	End	Strand	Gene Length (bp)	CDS (bp)	Amino Acid	Exon Number	pI	Mw (kDa)	Localization
MlongTPS1	scaffold3	25207839	25211071	−	3233	1635	544	7	5.08	62.93	Chloroplast ^a^/Cytoplasm ^b^
MlongTPS2	scaffold5	41734433	41737382	−	2950	1488	495	6	5.28	57.36	Chloroplast ^a^/Cytoplasm ^b^
MlongTPS3	scaffold5	41781767	41784235	−	2469	1638	545	6	4.99	63.01	Chloroplast ^a^/Cytoplasm ^b^
MlongTPS4	scaffold2	42600236	42604433	+	4198	1626	541	7	5.63	63.19	Chloroplast ^a^/Cytoplasm ^b^
MlongTPS5	scaffold2	42646914	42652153	+	5240	1626	541	7	5.56	63.06	Chloroplast ^a^/Cytoplasm ^b^
MlongTPS6	scaffold2	42808876	42813607	+	4732	1641	546	7	5.70	63.65	Chloroplast ^a^/Cytoplasm^b^
MlongTPS7	scaffold10	2519038	2521515	+	2478	1572	523	8	5.01	60.86	Chloroplast ^a^/Cytoplasm^b^
MlongTPS8	scaffold10	2869515	2871994	+	2480	1674	557	7	5.11	65.04	Chloroplast ^a^/Cytoplasm ^b^
MlongTPS9	scaffold10	3245887	3248093	+	2207	1311	436	8	5.82	51.00	Chloroplast ^a,b^
MlongTPS10	scaffold10	24101862	24105239	+	3378	1341	446	7	5.94	52.67	Chloroplast ^a^/Cytoplasm ^b^
MlongTPS11	scaffold10	26605063	26606857	−	1795	1155	384	6	6.97	44.60	Chloroplast ^a^/Cytoplasm ^b^
MlongTPS12	scaffold8	2619187	2622034	−	2848	1482	493	6	5.44	57.37	Chloroplast ^a^/Cytoplasm ^b^
MlongTPS13	scaffold8	2629991	2633116	−	3126	1563	520	7	5.59	60.59	Chloroplast ^a^/Cytoplasm ^b^
MlongTPS14	scaffold11	22094766	22101682	+	6917	2589	862	13	5.30	100.10	Chloroplast ^a,b^
MlongTPS15	scaffold11	22132562	22135423	+	2862	1791	596	7	5.26	69.43	Chloroplast ^a,b^
MlongTPS16	scaffold11	22353164	22356569	+	3406	1782	593	7	5.73	68.84	Chloroplast ^a,b^
MlongTPS17	scaffold11	22376541	22381192	−	4652	1560	519	10	5.78	60.82	Chloroplast ^a,b^
MlongTPS18	scaffold11	22424761	22430157	−	5397	1449	482	7	5.46	56.62	Chloroplast ^a,b^
MlongTPS19	scaffold11	29807062	29810465	+	3404	1782	593	7	5.65	68.76	Chloroplast ^a,b^
MlongTPS20	scaffold11	29816966	29822114	+	5149	1362	453	6	7.12	52.57	Chloroplast ^a,b^
MlongTPS21	scaffold11	29845320	29849984	−	4665	1320	439	8	5.79	51.21	Chloroplast ^a,b^
MlongTPS22	scaffold11	29920867	29925533	−	4667	1476	491	7	5.61	57.69	Chloroplast ^a,b^
MlongTPS23	scaffold4	34738619	34741984	+	3366	1374	457	7	5.74	53.33	Chloroplast ^a,b^
MlongTPS24	scaffold4	34742308	34744838	−	2531	1800	599	7	5.41	69.98	Chloroplast ^a,b^
MlongTPS25	scaffold5	285351	288259	+	2909	1734	577	7	5.18	67.16	Chloroplast ^a,b^
MlongTPS26	scaffold5	291563	294867	+	3305	1737	578	7	5.46	67.19	Chloroplast ^a,b^
MlongTPS27	scaffold5	296099	298389	−	2291	1383	460	5	5.78	53.55	Chloroplast ^a,b^
MlongTPS28	scaffold5	11506827	11509585	−	2759	1800	599	7	5.32	69.92	Chloroplast ^a,b^
MlongTPS29	scaffold5	11621067	11623817	−	2751	1800	599	7	5.43	69.91	Chloroplast ^a,b^
MlongTPS30	scaffold5	21893670	21898545	−	4876	1779	592	7	6.23	69.34	Chloroplast ^a,b^
MlongTPS31	scaffold2	19325281	19331000	+	5720	1737	578	7	5.36	67.30	Chloroplast ^a,b^
MlongTPS32	scaffold10	30749715	30752287	+	2573	1653	550	7	5.55	63.29	Chloroplast ^a,b^
MlongTPS33	scaffold10	30761480	30765652	−	4173	1599	532	8	5.55	62.05	Chloroplast ^a,b^
MlongTPS34	scaffold10	30776115	30779012	−	2898	1374	457	6	6.07	53.11	Chloroplast ^a^/Cytoplasm ^b^
MlongTPS35	scaffold10	30785670	30788296	−	2627	1590	529	7	6.77	61.55	Chloroplast ^a,b^
MlongTPS36	scaffold4	37761090	37769581	+	8492	2430	809	15	6.76	92.10	Chloroplast ^a,b^
MlongTPS37	scaffold9	4343490	4348710	−	5221	2409	802	14	5.95	91.97	Chloroplast ^a,b^
MlongTPS38	scaffold9	4410562	4415127	−	4566	2178	725	15	7.84	82.44	Chloroplast ^a,b^
MlongTPS39	scaffold9	4626769	4631237	−	4469	2304	767	14	5.84	87.25	Chloroplast ^a,b^
MlongTPS40	scaffold8	14598298	14605058	−	6761	2346	781	14	6.19	89.79	Chloroplast ^a,b^
MlongTPS41	scaffold9	4215819	4220540	−	4722	2085	694	13	5.65	80.41	Chloroplast ^a,b^
MlongTPS42	scaffold9	4297285	4301128	−	3844	1737	578	11	6.10	67.05	Chloroplast ^a,b^
MlongTPS43	scaffold9	4315863	4321588	−	5726	1755	584	11	5.48	67.38	Chloroplast ^a, b^
MlongTPS44	scaffold9	4400967	4404832	−	3866	1827	608	14	5.90	70.06	Chloroplast ^a,b^
MlongTPS45	scaffold9	4663702	4668738	+	5037	1752	583	14	5.43	66.94	Chloroplast ^a,b^
MlongTPS46	scaffold9	4696275	4699991	+	3717	1689	562	10	5.58	65.28	Chloroplast ^a,b^
MlongTPS47	scaffold9	4746792	4752673	−	5882	2295	764	14	5.88	87.58	Chloroplast ^a,b^
MlongTPS48	scaffold9	4791367	4793719	−	2353	1134	377	6	5.31	43.28	Mitochondrion ^a^/Chloroplast ^b^
MlongTPS49	scaffold9	4890741	4894353	−	3613	1734	577	10	5.69	66.69	Chloroplast ^a,b^
MlongTPS50	scaffold9	4940721	4944084	+	3364	1536	511	9	5.30	59.27	Mitochondrion ^a^/Chloroplast ^b^
MlongTPS51	scaffold9	4988299	4993896	+	5598	2292	763	14	5.77	87.38	Chloroplast ^a,b^
MlongTPS52	scaffold9	5111972	5115082	+	3111	1515	504	9	5.38	58.34	Mitochondrion ^a^/Chloroplast ^b^
MlongTPS53	scaffold9	7132180	7139762	+	7583	1755	584	11	5.38	67.56	Chloroplast ^a,b^
MlongTPS54	scaffold9	31439884	31443309	−	3426	1350	449	8	5.03	52.24	Chloroplast ^a,b^
MlongTPS55	scaffold9	31907037	31911201	−	4165	1533	510	9	5.09	59.61	Chloroplast ^a,b^
MlongTPS56	scaffold9	31917248	31919875	−	2628	1578	525	9	5.53	60.86	Chloroplast ^a,b^
MlongTPS57	scaffold8	2453217	2457977	−	4761	2322	773	14	5.62	88.21	Chloroplast ^a,b^
MlongTPS58	scaffold8	2469812	2471751	−	1940	1308	435	7	5.22	50.43	Chloroplast ^a,b^
MlongTPS59	scaffold10	30078136	30083625	−	5490	2478	825	12	5.99	94.00	Chloroplast ^a^/Cytoplasm ^b^
MlongTPS60	scaffold11	3129977	3133005	+	3029	1521	506	6	5.97	57.84	Unknown ^a^/Cytoplasm ^b^
MlongTPS61	scaffold3	44742988	44745414	+	2427	1572	523	7	7.04	61.62	Unknown ^a^/Cytoplasm ^b^
MlongTPS62	scaffold6	2272054	2274523	+	2470	1728	575	7	5.82	66.44	Unknown ^a^/Cytoplasm ^b^
MlongTPS63	scaffold6	15636480	15639592	−	3113	1764	587	7	5.31	66.38	Unknown ^a^/Cytoplasm ^b^

^a^ Predicted results of AtSubP tool. The prediction approach followed the best hybrid-based classifier (AA + PSSM + N-Center-C + PSI-BLAST).^b^ Predicted results of ProtComp.

**Table 2:** Statistics of *TPS* subfamily gene numbers in *M. longifolia*, *A. thaliana* and other Lamiaceae plants.

Species	Subfamily						Total
	a	b	c	e	f	g	
*M. longifolia*	13	22	5	18	1	4	63
*O. teruiflorum*	14	12	7	2	1	7	43
*S. indicum*	21	5	6	3	0	7	42
*S. miltiorrhiza*	32	21	5	2	1	3	64
*S. splendens*	52	30	7	7	2	6	104
*A. thaliana*	22	6	1	1	1	1	32

**Table 3:** Tests for selection among codons of *M. longifolia TPSs* using site models.

TPS SubFamily	Model	np	Ln L	Estimates of Parameters				Model Compared	LRT *p*-Value	Positive Sites
TPS-a	M3	29	−6662.29	*p*:	0.300	0.605	0.095	M0 vs. M3	0.000	[]
				ω:	0.047	0.287	0.782			
	M0	25	−6742.49	ω_0_:	0.225					Not Allowed
	M2a	28	−6701.40	*p*:	0.819	0.044	0.138	M1a vs. M2a	1.000	[]
				ω:	0.191	1.000	1.000			
	M1a	26	−6701.40	*p*:	0.819	0.181				Not Allowed
				ω:	0.191	1.000				
	M8	28	−6664.45	p_0_ = 0.989	*p* = 0.948	q = 2.701		M7 vs. M8	0.631	212 C 0.781
				p_1_ = 0.011	ω = 1.525					
	M7	26	−6664.91	*p*=	0.912	q=	2.472			Not Allowed
TPS-b	M3	47	−2367.77	*p*:	0.109	0.602	0.289	M0 vs. M3	0.000	[]
				ω:	0.000	0.228	0.612			
	M0	43	−2393.98	ω_0_:	0.289					Not Allowed
	M2a	46	−2382.37	*p*:	0.756	0.123	0.121	M1a vs. M2a	1.000	[]
				ω:	0.230	1.000	1.000			
	M1a	44	−2382.37	*p*:	0.756	0.244				Not Allowed
				ω:	0.230	1.000				
	M8	46	−2374.65	p_0_ = 1.000	*p* = 1.135	q = 2.498		M7 vs. M8	1.000	
				p_1_ = 0.000	ω = 1.000					
	M7	44	−2374.65	*p*=	1.135	q=	2.498			Not Allowed
TPS-c	M3	13	−9115.18	*p*:	0.548	0.420	0.032	M0 vs. M3	0.000	[]
				ω:	0.070	0.407	8.173			
	M0	9	−9231.50	ω_0_:	0.202					Not Allowed
	M2a	12	−9133.53	*p*:	0.779	0.166	0.055	M1a vs. M2a	1.000	[]
				ω:	0.129	1.000	1.000			
	M1a	10	−9133.53	*p*:	0.779	0.221				Not Allowed
				ω:	0.129	1.000				
	M8	12	−9115.20	p_0_ = 0.968	*p* = 0.772	q = 2.595		M7 vs. M8	0.000	8 F 0.567,16 A 0.551,19 L 0.515,28 Y 0.916,32 I 0.748,33 K 0.649,41 E 0.627,212 L 0.711,591 L 0.828,636 E 0.875,637 Q 0.838,639 M 0.851,640 A 0.712,641 A 0.611,643 V 0.944,647 D 0.627,654 K 0.738
				p_1_ = 0.032	ω = 8.049					
	M7	10	−9124.83	*p*=	0.673	q=	1.922			Not Allowed
TPS-e	M3	39	−6467.88	*p*:	0.300	0.539	0.160	M0 vs. M3	0.000	[]
				ω:	0.077	0.351	0.785			
	M0	35	−6537.92	ω_0_:	0.310					Not Allowed
	M2a	38	−6492.46	*p*:	0.739	0.167	0.095	M1a vs. M2a	1.000	[]
				ω:	0.231	1.000	1.000			
	M1a	36	−6492.46	*p*:	0.739	0.261				Not Allowed
				ω:	0.231	1.000				
	M8	38	−6468.70	p_0_ = 0.966	*p* = 1.035	q = 2.155		M7 vs. M8	0.858	45 R 0.514,234 V 0.633
				p_1_ = 0.034	ω = 1.000					
	M7	36	−6468.86	*p*=	0.962	q=	1.829			Not Allowed
TPS-g	M3	11	−5784.14	*p*:	0.284	0.560	0.156	M0 vs. M3	0.000	[]
				ω:	0.046	0.296	24.257			
	M0	7	−5866.96	ω_0_:	0.202					Not Allowed
	M2a	10	−5795.20	*p*:	0.652	0.232	0.117	M1a vs. M2a	1.000	[]
				ω:	0.134	1.000	1.000			
	M1a	8	−5795.20	*p*:	0.652	0.348				Not Allowed
				ω:	0.134	1.000				
	M8	10	−5784.63	p_0_ = 0.869	*p* = 0.935	q = 2.849		M7 vs. M8	0.008	15 K 0.532,141 C 0.547,177 N 0.551,294 R 0.510,299 W 0.517,363 R 0.524,423 D 0.501
				p_1_ = 0.131	ω = 31.804					
	M7	8	−5789.50	*p*=	0.716	q=	1.590			Not Allowed

## Supplementary Materials

The following are available online at https://www.mdpi.com/2073-4425/12/4/518/s1, Figure S1: Multiple sequence alignment of LS from *M. spicata*, *M. piperita*, and *M. longifolia*, File S1: Coding sequences of *M. longifolia TPSs*.
