# Correction: Chen et al. Genome-Wide Analysis of Terpene Synthase Gene Family in *Mentha longifolia* and Catalytic Activity Analysis of a Single Terpene Synthase. *Genes* 2021, *12*, 518

**DOI:** 10.3390/genes13010069

**Published:** 2021-12-28

**Authors:** Zequn Chen, Kelly J. Vining, Xiwu Qi, Xu Yu, Ying Zheng, Zhiqi Liu, Hailing Fang, Li Li, Yang Bai, Chengyuan Liang, Weilin Li, Bernd Markus Lange

**Affiliations:** 1 Institute of Botany, Jiangsu Province and Chinese Academy of Sciences (Nanjing Botanical Garden Mem. Sun Yat-Sen), Nanjing 210014, China; chenzq1219@cnbg.net (Z.C.); xiwuqi@cnbg.net (X.Q.); yuxu84@163.com (X.Y.); fanghailing2013@163.com (H.F.); xinwenbanlili@163.com (L.L.); baiyang.89@163.com (Y.B.); 2 Department of Horticulture, Oregon State University, Corvallis, OR 97331, USA; kelly.vining@oregonstate.edu; 3 Zhejiang Provincial Key Laboratory of Resources Protection and Innovation of Traditional Chinese Medicine, Hangzhou 311300, China; alice.zhengy@gmail.com; 4 Nanjing Foreign Language School, Nanjing 210008, China; lzq030729@gmail.com; 5 Jiangsu Key Laboratory for the Research and Utilization of Plant Resources, Nanjing 210014, China; 6 College of Forest, Nanjing Forestry University, Nanjing 210037, China; wlli@njfu.edu.cn; 7 Institute of Biological Chemistry and M.J. Murdock Metabolomics Laboratory, Washington State University, Pullman, WA 99164, USA; lange-m@wsu.edu

The authors have requested that the following changes be made to their paper [1].

The original authors wish to add Dr. Kelly J. Vining and Dr. Bernd Markus Lange as coauthors of this paper. The author contributions were: K.J.V. and B.M.L. contributed to this paper by providing the genome data of *M. longifolia*. The updated “Author Contributions” and “Funding” are provided below.

We would like to apologize for any inconvenience caused to the authors and readers by this mistake. The published version will be updated on the article webpage, with a reference to this notice.
