# Supplementary material for: Genome-Wide Analysis of Terpene Synthase Gene Family in Mentha longifolia and Catalytic Activity Analysis of a Single Terpene Synthase
Source: Genes (Basel). 2021 Apr 2;12(4):518. doi: 10.3390/genes12040518 (PMC8066702; doi:10.3390/genes12040518)
Supplement: Supplementary file 1 [file genes-12-00518-s001.zip › genes-1056921-supplementary/Figure S1.pdf]

```
MpLS        : MALKVFSGAMQMPIPSKLTTYLQPSHLNSSPKLLSNTKGTSRSRLRVSCSSSQLTTERRSGNYNPSRWDVDFIQTLH :  77
MsLS        : MALKVLSVATQMAIPSNLTTCLQPSHFKSSPKLLSSTNSSSRSRLRVYCSSSQLTTERRSGNYNPSRWDVNFIQSLL :  77
MlongTPS29  : MAFKVFSVATQMAIPSNLTTCLQPSHLKSSPKLLSSTNSSSRSRLRVYCSSSQLTTERRSGNYNPSRWDVDFIQSLH :  77

MpLS        : SDYKDEKHARRASELVTLVKMELEKETDQIRQLELIDDLQRMGLSDHFQNEFKEILSSVYLDHGYYKNPDPKEERDL : 154
MsLS        : SDYKEDKHVIRASELVTLVKMELEKETDQIRQLELIDDLQRMGLSDHFQNEFKEILSSIYLDHHYYKNPFPKEERDL : 154
MlongTPS29  : SDYKEDKHAIRASELVTLVKMELEKETDHIRQLELIDDLQRMGLSDHFQNEFKEILSSIYLDHHYYKNPFPKEERDL : 154

MpLS        : YSTSLAFRLLREHGFQVAQEVFDSFKNEEGEFKESLSDDTRGLLQLYEASFLLTEGETTLESAREFATKFLEERVNE : 231
MsLS        : YSTSLAFRLLREHGFQVAQEVFDSFKNEEGEFKESLSDDTRGLLQLYEASFLLTEGETTLESAREFATKFLEEKVNE : 231
MlongTPS29  : YSTSLAFRLLREHGFQVAQEVFDSFKNEEGEFKESLSHDTRGLLQLYEASFLLTEGETTLESAREFATKFLEERVNE : 231

MpLS        : GGGDENLLTRIAYSLEIPLHWRIKRPNAPVWIDSYRKRPNMNPVVLDLAILDLNIVQAHFQQELKESFRWWRNTGFV : 308
MsLS        : GGVDGDLLTRIAYSLDIPLHWRIKRPNAPVWIEWYRKRPDMNPVVLELAILDLNIVQAQFQEELKESFRWWRNTGFV : 308
MlongTPS29  : GGVDGDLLTRIAY LDIPLHWRIKRPNAPVWIEWYRKRPDMNPVVLELAILDLNIVQAQFQEELKESFRWWRNTGFV : 308

MpLS        : EKLPFARDRLVECYFWNTGIIEPRQHASARIMMGKVNALITVIDDIYDVYGTLEELEHFTDLIRRWDIDSIDQLPDY : 385
MsLS        : EKLPFARDRLVECYFWNTGIIEPRQHASARIMMGKVNALITVIDDIYDVYGTLEELEQFTDLIRRWDINSIDQLPDY : 385
MlongTPS29  : EKLPFARDRLVECYFWNTGIIEPRQHASARIMMGKVNALITVIDDIYDVYGTLEELEQFTDLIRRWDINSIDQLPDY : 385

MpLS        : MQLCFLALNNFVDETSYDVMKEKGVNVIPYLRQSWVDLADKYMVEARWFYGGHKPSLEEYLENSWMSISGPCMLTHI : 462
MsLS        : MQLCFLALNNFVDDTSYDVMKEKGVNVIPYLRQSWVDLADKYMVEARWFYGGHKPSLEEYLENSWQSISGPCMLTHI : 462
MlongTPS29  : MQLCFLALNNFVDDTSYDVMKEKGVNVIPYLRQSWVDLADKYMVEARWFYGGHKPSLEEYLENSWQSISGPCMLTHI : 462

MpLS        : FFRVTDSFTKETVDSLYKYHDLVRWSSFVLRLADDLGTSVEEVSRGDVPKSLQCYMSDYNASEAEARKHVKWLIAEV : 539
MsLS        : FFRVTDSFTKETVDSLYKYHDLVRWSSFVLRLADDLGTSVEEVSRGDVPKSLQCYMSDYNASEAEARKHVKWLIAEV : 539
MlongTPS29  : FFRVTDSFTKETVDSLYKYHDLVRWSSFVLRLADDLGTSVEEVSRGDVPKSLQCYMSDYNASEAEARKHVKWLIAEV : 539

MpLS        : WKKMNAERVSKDSPFGKDFIGCAVDLGRMAQLMYHNGDGHGTQHPIIHQQMTATLFEPFA : 599
MsLS        : WKKMNAERVSKDSPFGKDFIGCAVDLGRMAQLMYHNGDGHGTQHPIIHQQMTRTLFEPFA : 599
MlongTPS29  : WKKMNAERVSKDSPFGKDFIGCAADLGRMAQLMYHNGDGHGTQHPIIHQQMTRTLFEPFA : 599
```

Figure S1. Multiple sequence alignment of LS from *M. spicata*, *M. piperita*, and *M. longifolia*.
