# Supplementary material for: Genome-Wide Analysis of Terpene Synthase Gene Family in Mentha longifolia and Catalytic Activity Analysis of a Single Terpene Synthase
Source: Genes (Basel). 2021 Apr 2;12(4):518. doi: 10.3390/genes12040518 (PMC8066702; doi:10.3390/genes12040518)
Supplement: Supplementary file 1 [file genes-12-00518-s001.zip › genes-1056921-supplementary/File S1.docx]

File S1. Coding sequences of *M. longifolia TPSs*

>MlongTPS1

ATGGCTGAAATCTGTGCGTCGGCTGCTCCAATCTCAACAAAGAATACAAGTGTAGAGGAAATCCGTCGATCGGTAACATATCATCCCAGCGTTTGGAGAG

ATCATTTTCTTGCATATACTAACGATGTCACGGAAATCAGTGCTGCTGAGAAGGAACAACTCGAAAAGCAAAAGGAAAAGGTTAAGAATTTGCTAGCTCA

AACTCCAAATGATTCAACGCTCAAGATCGACCTCATCGATGCAATCCAACGTCTAGGGTTGGGCTATCATTTCGAAGAGGAAACCGACGGATCCTTGCGA

AAAATTCGCGACAGTTATGAAATGTTAAGTAGCAAAGGCGAGGACGATGTCCGTGTTCTTGCTCTTCGCTTTCGTCTGCTTAGACAACAAGGTTATCGCG

TCCCATGCGAAGTGTTCAACAAATTGGTAGACGACGAAGGGAATTTTAAGGAGTCGTTGATTAACGACGTTGAAGGGATGCTAAGCTTGTACGAAGCTTC

GAATTATGGAATAAATGGAGAGGAAATTATGGACAAAGCCTTAGAATTTTCTTCTTCTCATCTTGAGGATTCAATCCAAAAAAAACCCACTTGTCTTTCG

AGACGAGTGAAAGAAGCTTTGGATATGCCGATTAGCAAGACTTTGATGAGATTGGGAGCGAGAAAATTCATATCTATTTATGAAGAAGACGAGTCGCATA

ATGAATTATTATTGAATTTTGCAAAACTGGACTTCAATATAGTACAAAAGATGCATCAAAGAGAGTTGCACCATATTACAAGGTGGTGGGAAGGTTTAGA

CTTTAAAAGTAAACTACCTTTTGCAAGAGATAGAGTGGTGGAGTGCTACTTCTGGATTTTGGGAGTGTATTTTGAACCAAAATACGAAACTGCAAGAAGA

TTACTAACTAAAGTCATATCCATGGCTTCCATCCTTGATGATATATATGATGTTTATGGGAGTTTAGATGAACTCCGACATTTCACCGATGCTATTCAAA

GATGGGATATTATCGGTGCTGAAGAGTTGCCACCATACATGAGAATATGTTATGAAGCTTTACTAGGTGTATATGCCGAAATGGAAGATGAAATGATGAT

AAAATTGGTGAGGGCGTATATGGAAGAGGCGGAATGGTGTTATAGCAAGTATATTCCAAGGATGGATGAGTATATGAAACTGGCACTCGTATCCGGCGCT

TACATGATGCTATCAACAACTTCTTTAGTCGGGATGATGGAAGAACCCATCTCAGTACAAGATTTTGATTGGATCACCGGCGAACCACCCATCTTACGAG

CTGCATCCGTTATTTGTAGGTTGATGGACGACATGGTAGGCCATGGGATTGAGCAAAAAATTACGAGCGTGGATATTTACATGAGGGAAAACGGGTGCTC

GAAGACGGAAGCTTTTGGAGAGTTTTGGAAACGAGTGAAGAAAGCATGGAAGGATATGAATGAGGAGTGCCTGGAGCCAAGGCCAGCATCCATGCCCATA

CTTACTCGAGTTCTCAATCTTGCTCGCGTCATCAATTTATTGTACGTTGGTGAAGATGCCTATGGTAGTTCAAGTACTAAGACCAAAGATTTCATTCAAT

CCGTGCTCGTAGATCCCCTGCACTCAACTATTTGA

>MlongTPS2

ATGGCTTCCTCTGCTAATAACGTGCGCCCCGTAGCCAACTTCTCCCCAAGCCTATGGGGCGATCTCTTCACTTCATCTTCCATCAACGTTGTCCACAATC

ACGAGGAAGAAATCGAAGCATTGAAAGAGGAAGTAGCAAGAATGCTCGTCGCAAAGGATGCGACCCCGGCGGAGAAGCTGAAGCTCATCGACACCGTCGA

GCGCCTCGGGGTTTCGTACCACTTTGAGAAGGAAATCGACGAGCAGATTCGGTTTATGTACACTAATGCCTATCCAGGAAACGAAGACGACGACAGCAAC

CTGCAAACCGTCGCTCTGTATTTCAGGCTGCTCAGGCAACACGGCTACAGCATTTCTTCCGGTAAAGGAAATTTCTCCCTCAGCTCGATCGGTCGTATAC

CGATGAAGGAGCAAGTAGAGCATGCCCTCGAGCAATGCCTCCACCGCGGCCTCCTGAGAGTCGAGGCGCGAAGCTACGTGTTAGTATACCAAGAGCAGGA

GTCGAAGAACGAGTCGTTGTTGAGGCTGGCTAAACTGGACTTCAATATGGTCCAGGCTTTGCACAAAGAAGAACTCCGCGACTTAATGAGGTGGTGGAAG

GAGATGGATCTGGTTTTGAAGCTCGCTTATGCAAGAGATAGAATGGTGGAATGCTACTTCTGGGCATTGGGAGTTTACTTCGAGCCTCAATACTCTCGAG

CTCGAGTAATGTTGGCCAAAACTATTGCTATGATTTCAGTCATTGATGATACCTACGATTCCTATGGTACGGTTGATGAGCTTCAAATTTTCACAGATGT

TGTGCAAAGATGGGAAATGAAAGAAATGAACAAATTACCAGAGTACATGAAGATAATCTTCAGAGCTCTTCTCCAACTCTACGATGAGTACGAGGAGGAA

TTAAGGCCACAAGGAAGATCATTTGCAGTTGATTATGCAAAAGCCACAATGCAAGAGATCGTGAGAAGCTACAACATTGAGGCGAAATGGTTCATCGAAG

GCTACACGCCTCCGTTCCTCGACTACATGGCCAACGGCTTCATCACCGGCACCTACTACTTGCTCGGCGCCACGTCCTTCGTCGGGATGAGCTCCGTGAC

GAAAGAAGCGTTTGATTGGCTCATCAACAAGCCCATCATCCAAGTAGCTAACGTCATCATCGGTCGAGTTATCGACGACGTCGCAACTTATGAGATTGAG

AAGAAAAGAGGACAGAGTGCGACGGGAATCGAATGCTACATGAAAGATCACGGCGTGTCGAAGGAGGAGGCCATGGAGGAGTTCAAGAAAATGGCGGAGG

ATGCATGGAAAGATATGAACGGTGAAATATTTAAGGAGAAGTCGGTTTCGATGGAGGTTATGAAGAGGATAGTGAATCTTTCTCGATTGATCGACGTTGC

TTACAAGAATAATCAAGATGGATATACTCATCCCGAGAAAGTGTTGAAGCCCATTGTTCATGCATTGCTCGTCGACTCACTTCATTAG

>MlongTPS3

ATGGCTTCCTCTGCTAATAACGTGCGCCCCTCTGCCAACTTCTCCCCAAGCCTATGGGGCGATCTCTTCACTTCATCTTCCATCAACGTTGTCCACAATC

ACGAGGAAGAAATCGAAGCACTGAAAGAGGAAGTAGCAAGAATGCTCGTCGCAAAGGATGAGACTGTGGCCGAGAAGCTGAAGCTCATCGACACCGTCGA

GCGCCTCGGGGTTTCGTACCACTTCGAGAAGGAAATCGACGAGCAGATTCGGTTTCTGTACACTAATGCCTATCCAGGAAACGAAGACGACGACAACAAC

CTGCAAACCGTTGCTCTGTATTTCAGGCTGCTTAGGCAACACGGCTACAACATTTCTTCCGGTATTTTCGAGAAATTCAGATCACAAGTTGGTGGGAGGT

TCGATGATGGCGTGAAGGACGACGATGTTGAGGGAATGTTGAGCCTATACGAAGCTGCGCATTTAAGAACGCACGGCGAACAAGTTCTCGACGAGGCCTT

CGCCTTCGCCACGGCTCGTCTCGAGTCGATTGCTCCGTTTCTGAGCTCTCCGATGAAGGAGCAAGTAGAGCATGCCCTCGAGCAATGCCTCCACCGCGGC

CTCCCGAGAGTCGAGGCGCGAAGCTACGTGTTAGTATACCAAGAGCAGGAGTCGAAGAACGAGTCGTTGTTGAGGCTGGCTAAACTGGACTTCAATATGG

TCCAGGCTTTGCACAAAGAAGAACTCCGCGAATTGATGAGGTGGTGGAAGGAGATGGATCTGGTTTCGAAGCTCACTTATGCAAGAGATAGAATGGTGGA

ATGCTATTTCTGGGCATTGGGAGTTTACTTCGAGCCTCAATACTCTCGAGCTCGAGTAATGTTGGCCAAAACTATTTCTATGATTTCAGTGATTGATGAT

ACCTACGATTCTTATGGTACGGTTGATGAGCTTCAAATTTTCACAGATGCTGTGCAAAGATGGGAAATGAAGGAAATCAACAAATTACCAGAGTACATGA

AGATAATCTTCAGAGCTCTTCTCCAACTCTACGATGAGTATGAGGAGGAATTAAGGCCACAAGGAAGATCAGTTGCAGTTGATTATGCAAAAGCCACAAT

GCAAGAGATTGTGAGAAGCTACAACATTGAGGCGAAATGGTTCATCGAAGGCTACATGCCTCCGTTCCTCGACTACATGGCCAACGGCTTCGTCACCGGC

ACCTACTACTTGCTCAGCGCCACGTCCTTTGTCGGGATGAGCTCCGTGACGAAAGAGGCCTTCGATTGGCTCATCAACAAGCCCACCATCCAAGTAGCTA

ACGTCATTATTGGTCGAGTTATCGACGACGTCGCAACTTATGAGATTGAGAAGAAAAGAGGTCAGAGCGCGACGGGAATCGAATGTTACATGAAAGATCA

CGGCGTGTCGAAGGAGGAGGCGATGGAGGAGTTCAAGAAAATGGCGGAGGATGCATGGAAGGATATGAACGGTGAAATATTTAAGGAGAAGTCTGTTTCG

ATGGAGGTTTTGGAGAGGATAGTGAATCTTTCTCGATTGATCGACGTTGCTTACAAGAATAATCAAGATGGATATACTCATCCTGAGAAAGTGTTGAAGC

CCATCATTCATGCATTGCTCGTCGACTCACTTCATTAG

>MlongTPS4

ATGGCAGCCAGTAATGTTGTAAGTTGCTTGAGGGATGTAAGGCCACCTATTACCAAGTATGCGCCAAGCATGTGGACTCATACTTTCTCTAACTTTTCTT

TTGACGAACAGGAACAACAAAAGTACGCGGAAACAATTGAAGCGCTGAAGCAAGAAGTAAGAGGCATGCTTATGGCTACACCCACGCCTCTCAAACAAAT

GATGCTAATCGACACTCTCGAGCGTTTGGGATTGGCTTACCATTTCAAGACGGAGATCGATAACAAAATCGCACATATCAATAACGCAGAAGTCGACGTT

CTTCAAGACAATGATTTGTTCGCTACTGCACTTCGATTCCGTTTGCTCAGACAACATCGACACCACGTTTCTTGCGATGGTATGTTTGAGGAAGGCCTCC

GCAGAGATGATGAAGGGCTATTAAGCTTGTATGAAGCAGCTCATGTTAGATTTCGTGGGGAAAAAATATTACAAGAGGCTGTGAATTTTACAAGGCATTA

TTTGAGACGTGTGGAAGCAGAGTTAGAGTCTCCACTTAAGGAGAAAGTGAAGCGAGCTTTGGAGCACCCTCTTCACTGGGAATTGCCCATTGTCTCTGCA

CGCCTTTTCATCTCCATTTACGAAAAGGATGACTCTAGAAATGAATTGCTTCTCAAATTAGCAAAAATTAACTTCAAATTTATGCAGAATGTGTACAGGA

ATGAGCTCTCCCAACTCTCAAGGTGGTGGAACAAATTTGATCTGAAATCAAAATTACCATACGCAAGAGATCGACTGGTTGAGGCCTACCTTTGGGCTAT

AGGATACAATTATGAACCTCAATCTTCTTATGTTCGAATTGGACTTGTCAAAGGCATACAAATTATTGGAGTTATGGATGATACATATGATAATTATGCC

ACAGTGAATGAAGCTCAACTTTTTACTGAAGTCTTGGACAAATGGAATGCAGAAGAGGCTGAAAGACTCCCAGAATATATGAGAATTGTTTATCATTTTA

TTTTGAGTATATCTGAAGATTATGAACGTGATGCGATGAAACTTGGGAAAAGCTTTGCAACTCCTTATTTCAATGAATCGGTGAAACAACTTGCAAGGGG

TTACAACCAAGAGCTAAAGTGGGTTATGGAAAGACAACTACCTCCATTCCAAGACTACGTAAAAAATTCAGAGATTACTAGTTGTGTTTATCTCATGTTT

GCTTCAATTATCCCGGGCTTGAAATCTGTTACCCAAGAAACCATTAATTGGGTGAAGAGTGAACCCAAGCTCGCACGATCAACTGCTATGGTTGGTCGAT

ATGCGGATGACATTGGCTCTCAGCACCGCGAGAACAAAGAAGGAAATATATTCACTTCATTAGATTGCTACATGAAACAATACGGCATTTCGAAACAAGA

GACGCTATTCAAGTTTGCGGAACTACTTGAGGAAGAGTGGGAGAATCTAAACAAGGAATTCGTCGTCGCAACAGTTTCTGTGCCTAAGGAAATTACCATT

ACATTCCTCAACTATGCTCGGATGTGCGACGCCACTTACAACCAAAATAATGGAGACGCTTATACAGATCCTAATGTTGCCAAGACAAATGTTGTTACTC

TCTTTGTTGATGACATAGTCATTTGA

>MlongTPS5

ATGGCAGCCAATAATGTTGTAAGTTGCTTGAGGGATGTAAGGCCACCTATTACCAAGTATGCGCCAAGCATGTGGACTGATACTTTCTCTAACTTTTCTT

TTGACGAACAGGAACAACAAAAGTACGCGGAAACAATTGAAGCGCTGAAGCAAGAAGTAAGAGGCATGCTTATGGCTACACCCACGCCTCTCAAACAAAT

GATGCTAATCGACACTCTCGAGCGTTTGGGATTGGCTCACCATTTCGAGACGGAGATCGATAACAAAATCGCACATATCAATAACGCAGAAGTCGACGTT

CTTCAAGACAATGATTTGTTCGCTACTGCACTTCGATTCCGTTTGCTCAGACAACATCGACACCACGTTTCTTGCGATGGTATGTTTGAGGAAGGCCTCC

GCAGAGATGATGAAGGGCTATTAAGCTTGTATGAAGCAGCTCATGTTAGGTTTCGTGGGGAAAAAATATTACAAGAGGTTGTGAATTTTACAAGGCATTA

TTTGAGACGTGTGGAAGCAGAGTTAGAGTCTCCACTTAAGGAGAAAGTGAAGCGAGCTTTGGAGCACCCTCTTCACTGGGAATTGCCCATTGTCTCTGCA

CGCCTTTTCATCTCCATATACGGAAAGGATGACTCTAGAGATGAATTGCTTCTCAAATTAGCAAAAACTAACTTCAAATTTATGCAGAATGTGTACAGGA

ATGAGCTCTCCCAACTCTCCAGGTGGTGGAACAAATTTGATCTGAAATCAAAATTACCATACGCAAGAGATCGACTGGTTGAGGACTACCTTTGGACTAT

AGGATACAATTATGAACCTCAATCTTCTTATGTTCGAATTGGACTTGTCAAAGGCATACAAATTATTGGAGTTATGGATGATACATATGATAATTATGCC

ACAGTGAATGAAGCTCAACTTTTTACTGAAGTCTTGGACAAATGGAGTGCAGAAGAGGCTGAAAGACTCCCAAAATATATGAGAATTGTTTATCATTTTA

TTTTGAGTATATCTGAAGATTATGAACGTGATGCGATGAAACTTGGGAAAAGCTTTGCAACTCCTTATTTCAATGAATCGGTGAAACAACTTGCAAGGGG

TTACAACCAAGAGCTAAAGTGGGTTATGGAAAGACAACTACCTCCATTCCAAGACTACGTTAAAAATTCAGAGATTACTAGTTGTGTTTATCTCATGTTT

GCTTCAATTATCCCGGGCTTGAAATCTGTTACCCAAGAAACCATTAATTGGGTGAAGAGTGAACCCAAGCTCGTACGATCAACTGCTATGGCTGGTCGAT

ATGCGGATGACATTGGCTCTCAGCACCGCGAGAACAAAGAAGGAAAAATATTCACTTCATTAGATTGCTACATGAAACAATACGGCGTTTCAAAACAAGA

GACGCTATCCAAGTTTGTTGAACTACTCGGGGAAGAGTGGGAGAATCTAAACAAGGAATTCGTCGTCGCAACAGTTTCTGTGCCTAAGGAAATCACCATT

ACATTCCTCAACTATGCTCGGATGTGCGACGCCACTTACGACCAAAATAATGGAGATGCTTATACAGATCCTAATGTTGCCAAGACAAATGTTGTTACTC

TCTTTGTTGATGACATAGTCATTTGA

>MlongTPS6

ATGTCTTGTAGTAGAATGGCAGCCAGTAATGTTGTAAGTTGCTTGAGGGATGTAAGGCCACCTATTACCAAGTATGCGCCAAGCATGTGGACTGATACTT

TCTCTAACTTTTCTTTTGACGAACAGGAACAACAAAAGTACGCGGAAACAATTGAAGCGCTGAAGCAAGAAGTAAGAGGCATGCTTATGGCTACACCCAC

GCCTCTCAAACAAATGATGCTAATCGACACTCTCGAGCGTTTGGGATTGGCTCACCATTTCGAGACGGAGATCGATAACAAAATCGCACATATCAATAAC

GCAGAAGTCGACGTTCTTCAAGACAATGATTTGTTCGCTACTGCACTTCGATTCCGTTTGCTCAGACAACATCGACACCACGTTTCTTGCGATGGTATGT

TTGAGGAAGGCCTCCGCAGAGATGATGAAGGGCTATTAAGCTTGTATGAAGCAGCTCATGTTAGGTTTCGTGGGGAAAAAATATTACAAGAGGTTGTGAA

TTTTACAAGGCATTATTTGAGACGTGTGGAAGCAGAGTTAGAGTCTCCACTTAAGGAGAAAGTGAAGCGAGCTTTGGAGCACCCTCTTCACTGGGAATTG

CCCATTGTCTCTGCACGCCTTTTCATCTCCATTTACGAAAAGGATGACTCTAGAGATGAATTGCTTCTCAAATTAGCAAAAACTAACTTCAAATTTATGC

AGAATGTGTACAGGAATGAGCTCTCCCAACTCTCCAGGTGGTGGAACAAATTTGATCTGAAATCAAAATTACCATACGCAAGAGATCGACTGGTTGAGGC

CTACCTTTGGGCTATAGGATACAATTATGAACCTCAATCTTCTTATGTTCGAATTGGACTTGTCAAAGGCATACAAATTATTGGAGTTATGGATGATACA

TATGATAATTATGCCACAGTGAATGAAGCTCAACTTTTTACTGAAGTCTTGGACAAATGGAATGCAGAAGAGGCTGAAAGACTCCCAAAATATATGAGAA

TTGTTTATCATTTTATTTTGAGTATATCTGAAGATTATGAACGTGATGCGATGAAACTTGGGAAAAGCTTTGCAACTCCTTATTTCAATGAATCGGTGAA

ACAACTTGCAAGGGGTTACAACCAAGAGCTAAAGTGGGTTATGGAAAGACAACTACCTCCATTCCAAGACTACGTTAAAAATTCAGAGATTACTAGTTGT

GTTTATCTCATGTTTGCTTCAATTATCCCGGGCTTGAAATCTGTTACCCAAGAAACCATTAATTGGGTGAAGAGTGAACCCAAGCTCGTACGATCAACTG

CTATGGTTGGTCGATATGCGGATGACATTGGCTCTCAGCACCGCGAGAACAAAGAAGGAAAAATATTCACTTCATTAGATTGCTACATGAAACAATACGG

CGTTTCAAAACAAGAGACGCTATCCAAGTTTGTTGAACTACTCGGGGAAGAGTGGGAGAATCTAAACAAGGAATTCGTCGTCGCAACAGTTTCTGTGCCT

AAGGAAATCACCATTACATTCCTCAACTATGCTCGGATGTGCGACGCCACTTACAACCAAAATAATGGAGATGCTTATACAGATCCTAATGTTGCCAAGA

CAAATGTTGTTACTCTCTTTGTTGATGACATAGTCATTTGA

>MlongTPS7

ATGGAATTTTATTCCCCAATTATTCCTGCAACTGCAATCAAAGATGTGAAGAAGTTGAATGAAATTCGAAAATCCGCCAAATTCCATCCAACTATTTGGG

GAGACTATTTTCTAGCTTATAATTCTGATAATACGCTAACCTCTGATGGTGAAGAAAATGAAGTTGCAAAGCAGAAAGAAATGGTACGGAAACTGCTAGC

TGAAGCCCCAGAAAATTCGACTAAAGACGACGATCTCCACACCGTGGCTCTTCGTTTTCGTTTGCTCAGACAACAAGGTTATAACGTCCCATGCGATGTT

TTCCGCAAATACATTGACAGTGAAGGAAATTTTATGGAGTCGTCGAAAGAGAATGTTGAAGGTTTGTTGGAATTATACGAGGCATCGCATCTCGCGACAC

GTGGCGAGGAAATTCTGAATAGAGCAATGGAGTTTTGTTCTTCCCATCTCCAAACATTAGTGAATCAGCAGTTGGTGGAAAATGTTTCTCTCTCTAAACG

TGTTAGCGAAGCTCTGAAGATGCCAATTCGCAGGAGTCTTACAAGATTGGGTGCCAGAAAGTTCATCTCTCTATACGAAGAGGATGATTCGCACAATGAA

ATGCTTTTGAATTTTGCGAAATTGGATTTCAATATTGTGCAGAAGATGCACCAGAGAGAGCTCAGTGATGCTACAAGGTGGTGGAAAAAATTGGAGGTGG

CAAATAAAATGCCTTACGCGAGAGACAGAATTGTGGAGTGCTTCTTTTGGATTGTGGGGGTCTACTTCGAGCCATGCTATGCCACTGCAAGAAGAATATT

GATTAAAAGCATAAGTATGGCTTCCATTATTGATGACACCTACGAATATGCAACCCTAGATGAACTGCAAATTCTAACTGATGCTATTCAACGTTGGGAT

GTTAACGAGACGTTGGAAGATTCGCCACCGTACATCCAAATGTGCTACAGAAGCCTTATTCGATCTTATGCTGAAATAGAAGATGAAGTAGTGAAGAAGA

ACTCGGAAGAATCGTACCGTGTCCAATATGCAATACAAGATATGAAAAAATTGGTGATGGCATATTTTGAAGAGGTGAAATGGTTGTACAGTAATAGTAT

TCCAACAGTGGAGGAATATATGAAGGTGTCACTCGTATCTTGTGGTTACATGATGTTGTCAATCACTTCTTTAGTTGGTATGGGGACTAATCAAGTTAGC

AAAGCTGATTTTGATTGGATTGTAAATGAACCTCTAATTGTTCGAGCATCCTCAGTAATTTTTCGACTAATGGACGACTTAGTCGGAGACGAGTATGAGG

AGAAGCCGTCGTCGGTCCATTGTTACATGAAGGAATATGGAATGTCGAAGGAGGAAGCTCGAACTCAACTCGAAGAACAAGTGAAAGGGGCATGGAAGGA

TATGAATGAAGAATGCCTCGAGCCGAGACCAGCCTCGATGCAAATCCTTATGCGCGTTTTGAATTTTGGTCGAGTCATAAATCTTCTGTATGCAGAAGAC

GATTGCTATGCCAATCCCATTAATTCCAAAGAATGGGTGAAGATGGTGCTTGCTGAGCCTGTTCCCATTTGA

>MlongTPS8

ATGGAATTTTATTCCCCAATTATTCCTGCAACTGCAATCAAAGATGTGAAGAAGTTGAATGAAATTCGAAAATCCGCCAAATTCCATCCAACTATTTGGG

GAGACTATTTTCTAGCTTATAATTCTGATAATACGCTAACCTCTGATGGTGAAGAAAATGAAGTTGCAAAGCAGAAAGAAATGGTACGGAAACTGCTAGC

TGAAGCCCCAGAAAATTCGACGTACAAAATGGAACTCATCGATACAATCCAGCGACTGGGAGTGGAATATCATTTTGAAAAAGAAATTGAAAAATTCTTA

AAACATATTCATGAAAATTATGTCCACCAAAATAGTAAAGACGACGATCTCCACACCGTGGCTCTTCGTTTTCGTTTGCTCAGACAACAAGGTTATAACG

TCCCATGCGATGTTTTCCGCAAATACATTGACAGTGAAGGAAATTTTATGGAGTCGTCGAAAGAGAATGTTGAAGGTTTGTTGGAATTATACGAGGCATC

GCATCTCGCGACACGCGGCGAGGAAATTCTGAATAGAGCAATGGAGTTTTGTTCTTCCCATCTCCAAACATTAGTGAATCGGCAGTTAGTAGAAAATGTT

TCTCTCTCTAAACGTGTTAGCGAAGCTCTGAAGATGCCAATTTGCAGGAGTCTTACAAGACTGGGTGCCAGAAAGTTCATCTCTCTATACGAAGAGGATG

ATTCGCACAATGAAATACTTTTGAATTTTGCGAAATTGGATTTCAATATTGTGCAGAAGATGCACCCAGAGAGAGCTCAGTGGTGGAAAAAATTGGAGGT

GGCAAATAAAATGCCTTACGCGAGAGACAGAATTGTGGAGTGCTTCTTTTGGATTGTGGGGGTCTACTTCGAGCCATGCTATGCCACTGCAAGAAGAATA

TTGATTAAAAGCATAAGTATGGCTTCCATTATTGATGACACCTACGAATATGCAACCCTAGATGAACTGCAAATTCTAACTGATGCTATTCAACGTTGGG

ATGTTAACGAGACGTTGGAAGATTCGCCACCGTACATCCAAATGTGCTACAGAAGCCTTATTCGATCTTATGCTGAAATAGAAGATGAAGTAGTGAAGAA

GAACTCGGAAGAATCGTACCGTGTCCAATATGCAATACAAGATATGAAAAAATTGGTGATGGCATATTTTGAAGAGGTGAAATGGTTGTACAGTAATAGT

ATTCCAACAGTGGAGGAATATATGAAGGTGTCACTCGTATCTTGTGGTTACATGATGTTGTCAATCACTTCTTTAGTTGGTATGGGGACTAATCAAGTTA

GCAAAGCTGATTTTGATTGGATTGTAAATGAACCTCTAATTGTTCGAGCATCCTCAGTAATTTGTCGACTAATGGACGACTTAGTCGGAGACGAGTATGA

GGAGAAGCCGTCGTCGGTCCATTGTTACATGAAGGAATATGGAATGTCGAAGGAGGAAGCTCGAACTCAACTCGAAGAACAAGTGAAAGGGGCATGGAAG

GATATGAATGAAGAATGCCTCGAGCCGAGACCAGCCTCGATGCAAATCCTTATGCGCGTTTTGAATTTTGGTCGAGTCATAAATCTTCTGTATGCAGAAG

ACGATTGCTATGCCAATCCCATTAATTCCAAAGAATGGGTGAAGATGGTGCTTGCTGAGCCTGTTCCCATTTGA

>MlongTPS9

ATGGTGAAGAAGAAGAAGTTGCAAAGCAGAAAGAAATGCAAAGACGACGATCTTCACACCGTGGCTCTTCGTTTTCGTTTGCTTAGACAACAAGGTTACA

ACGTCCCATGCGACGTTTTCCGCAAATTCACCGACCGTGAAGGAAATTTTTCAGCGGCGCTGAGAAATGACGTTGAAAGTTTGTTGGAATTATACGAGGC

GTCGCATCTCGCGACACGTGGCGAGGAAATTCTGGATAGAGCAATGGAGTTCTGTTCTTCCCATCTCCAAGCATTAGTAAATCAGCAGTTGGTGAACAAT

GTTTCTCTCTCTAAACGAGTGATTGAAGCTCTGAAGATGCCAATTTCCAAGAGTCTCACGAGATTGGGTGCCAGAAAGTTTATCTCTCTATACGAAGAGG

GCGATTCGAATAATGAAATACTTTTAAATTTTGCAAAAATAGATTTCAATATAGTGCAGAAGATGCACCAGAGAGAGCTCAGTGATGCTACGAGGTGGTG

GAAGAAATTGGATGTGGCGAATGCAATGCCTTACGCAAGAGATAGATTAGTGGAATGCTTCTTTTGGATGGTGGGGGTCTACTTCGAGCCATGCTATGCT

ACTGCTAGAAGAATATTAATTAAAAGCATAAGTATGGCTTCCATTATTGATGATACCTACGAATATGCAACCCTAAATGAACTGCGAATCCTCACTGACG

CTATTCAACGTTGGGATGTTAACGAGACATTGGAGGATTCTCCAGCGTACGTACAAATGTGCTACAGAAGCCTTATTCAAACTTATGCTGAAATAGAACA

TGAAGTACGGAAGAATAACTCAGAAGAATTGTACCGCGTCCAATATGCATTACAAGATATGAAAAAATTGGTGATGGCATATTTTGAAGAGGCAAAATGG

TTGTATGATGATTATATGCCAACATTTGAGGAGTATATGAAGGTAGCACTGGTATCTAGTGGCTACATGATGTTGTCAACAACTTCTTTAGTTGTAATCT

GTCGTGTAACTGACGATTTAGTCGGAGATGAGTATGAGCAGAAACCCTCGTCAATTCTATGTTACATGAAACAATATGGAGTAAGTGAGGAGGAAGCTCG

AGCTCAACTTGGACAACAAGTGAAGAATGCATGGAAGGACATGAACCAAGAATGCCTCGAGCCAAGACCGGCCTCGATGCAAATCTTAATGCGCGTTATC

AATCTCAATCGAGCCATAGATCTACTCTATTCGAGCCACGATTGCTATAGCGATCCACACAAATCGAAAGATTGGGTGAAGATGGTGCTTGTTGAACCTA

TCACAATTTGA

>MlongTPS10

ATGGAGATGAAGAATCAAAGTGGTGTTGTCACAACTTCATCTCGCCCTCTTGCAAATTATCACCCAAATATCTGGGGAGACCGCTTCCTACTCTACACAC

CCGAGTCATCCATGGCTGGCAAAGAAGAATCAATTGAAAAGCTAAAAGAGGAAGTGAAAGAAGAGATAAGGGAAATACCAGCAAACGACGTCGTAAGGCT

ACTAAAAACGATCGATGCAATACAAAGATTAGGCATCAAGTATCATTTTGAGGAGGACATTGATGAAAACCTGCAAAATTTGTCCCAAAAATTTGAAGAG

TACTGCAAAGACAATGACGATATGCTCATCACAGCTCTTGGTTTTCGGATTCTGAGGCAACATGGGCACCGAATCTCTTGCGAATCAGCACTGCCACGTG

TGAGCAATCCCATAGCTGAGCAAGCCGATCACGCGCTACACCAATACTCCAATCGTAGGGGATTGACACGTGTCGAGGCGAGACACTACATCTCCATCTA

CGGCCAATACGCCTCCCATCATCCAGGATTGCTCAGGCTTGCTAAGCTGGACTTCAACCTCCTTCAGTCTTTGCACAAAAGGGAGTTGAGCCTCCTCTAT

AGGTGGTTGAAAGATTTAGAGGTTCCGACAAAATTGTGGTATGCAAGAGATCGAATGGTGGAGGGCTACTTTTGGGTAATGGGAGTCTATTTTGAACCAA

AATTTGGTTTGGCAAGGAACATTTTAACCAAATTGCTGGCCATAGCGTCTCTATTTGATGATACTTTCGACGCATATGCTACTTTTCAAGAACTACAACT

CTTCGTGGAAGCAATGGAAGGGTGGAGCTATTCGTGTTTGGAGAAACTTCCGGACTATATGAAAATAATATACAAGGCACTCTTGGAAACATTTGAAGAA

ATTGAGGAACACATGATCAAATTGGGAACTTCTTATCGCCTCAACTATGGAATCGAAGCAATGAAAGTCGTGGCTCGAGACTACTTCGCTGAGGTTAAAT

GGAGGGAAGAAAAGTACAAACCAACAAGCGAGGAGTACATGCAGTTTGAGCAGAAAAGGGAGCACATACCTTCAATTTCAATAGTAGAGTGCTATAGAGA

AGAACACAAGAAATCAAAAGCAGAAGCGGTTTATGAATTCCGGAATCTGATGGAGGCGGCGTGGAAGGACATAAACGAGGCTTTCTTGAAACCAACTAAA

ATTCCGACCCCTCCACTCTATCGTATTCTCAATTTCACCCGTGTAATTGAGGTTATTTACAGTAAGGGCGATTGGTACACGCACGTAGGTCCTCAAATGC

AATTTTTCATCACCCAACTTCTCATCGACCCCGTTTCTTGA

>MlongTPS11

ATGAACGATGTTGAAGGGATATTGAGCTTATACGAGGCATCAAATTTTGGAGTGCATGGAGAAGAAATTCTTGACAAAGCATTAGGTTTTTGTTCGTCTC

GTCTCGAATCCTTAGTCACTAACATGAATAATAGTTGTCTTTCAAGACAAGTTAAGGAAGCTTTGAAGATCCCAATTAGCAAGACTCTAACAAGGTTGGG

AGCAAGAAAGTTCATTTCTATGTATCGAGAAGTCGACTTACACAACGAAAAATTACTCAACTTTGCCATATTGGACTTCAACCTAGTACAAAGGCTACAT

CAGAATGAGCTTAGCCATCTTACAAGGTGGTGGAAGGAATTAGACTTTGCAAATAATCTATCTTTTGCTAGAGATAGACTTGTGGAATGCTATTTTTGGA

TTATGGGAGTTTATTTTGAGCCGCGGTTCGGTATTGCACGAAAATTACTAACCAAAGTCATTTATATGGCTTCCGTCCTTGATGACATTTACGACGTGTA

TGGAACTTTGGACGAACTAATGCTTTTCACGACCATTGTTCGAAGGTGGGACATTAGTGCTATTGATCAATTGCCGCCATACATGAGAATATACTTGAAA

GCCCTTTTCGATGTGTATGTTGAAATGGAAGGAGAAATGGGAAAAATAGGCAAATTATACGCAGTTGAATATGCAAAGGAAGAGATGAAAAGATTAGGCG

AGATGTACCTTGAAGAGGCAAAATGGTCCTTTAGCAAGTACAAGCCCACAATGCGAGAGTACATGAAGGTGGCTCTTTTATCATCGGGGTACATGATGCT

GACGCGAACCGCCAATTCTAAAGTCATCGCAGATGATTACAAGATTAATGGACGACCTTACCTTGCAGGATATGGGTTTGAAGAGAAAAACTCAGCAGTG

CATTACTACATGAATGAAAAAGGCGTGTCGGAGGAGGAAGCTATTGCGCTCCGGAAACAAGTGGAGAAGTCATGGAAGACTCTAAATAAGGAATGCTTAG

AGCCAAGAGCAGCCTCCATGCCCATCCTTAAGTGTGTTGTGAATTTTACTCGTGTCATAGTTGTGTTATACACAGATGAAGATGCATACGGAAATTCCAA

AACTAAAACCAAAGATATGATCAAATCCGTACTCGTTGATCCCCTAACAGTTTAG

>MlongTPS12

ATGCTTATAGCTGCAACCACGCCTCTCCAACAAATGACACTAATCGACACTCTCGAGCGTTTGGGATTGTCTTTCCATTTTGAGACGGAGATCGAATACA

AAATCGAACAAATCAATGCTGCAGAAGACGACGGCTTCGATTTGTTCGCTACAGCTCTTCGGTTCCGTTTGCTCAGACAGCATCAACGCCGCGTTTCTTG

CAATGTTTTCGACAAGTTTGTCGACAAATATGGCAGGTTTGAAGAATCCCTTAGCAAAAATGTTGAAGGCCTATTGAGCTTGTACGAAGCATCTCATGTT

GGATTTCGCGACGAAAAAATATTACAAGAGGCTGTAAAATTTACGAGGCAGCAGTTGATATGCATGGAAGGAGAGTTAGAGTCTCCACTATTGATTAAAG

AGAAAGTGAAGCGAGCTTTGGAGCACCCTCTTCATAGGGATTTCCCCATCGTCTACGCACGCCTTTTCATCTCCATTTACGAAATGGATGACTCTAGAGA

TGAATTACTTCTCAAACTATCCAAAATTAACTTCAACTTCATGCAGAATTTGTATAAGAAAGAGCTCTCCCAACTCTCCAGGTGGTGGAGCACATGGGAT

CTGAAATCAAAATTACCATATGCAAGAGATCGAGTCGTGGAGGCTTACGTTTGGGGGGTAGGATACCATTACGAACCCAGGTACTCATATGTTCGAATGG

GACTTGCCAAAGGCATACAAATTATTGGAATCATGGATGATACATATGATAATTATGCTACACTCAATGAAGCTCAGCTTTTTACTCAAGTTTTAGACAA

GTGGGATAGAGATGAAGCTGATCAACTCCCGGAATACATGAAAATCGTTTATGAATTTATTTTGAGTACATGTGAAGATTATAAACGTGATGCAGTGAAA

CTTGGAAAAAGCTTTGCAGCTCCTTATTTCAACGAAACCGTGAAACAACTTGCAAGGGCATACAATCAAGAGCTGAAGTGGGTCATGGGAAGACCGTTGC

CTTCGTTCCAAGACTATGTAAAAAACTCAGAGATAACTAGTTGCATCTATATAATGTTTGCTTCTATTATCCCGGGCTTAAAATCTGTGACCCAAGAAAC

CATTGACTGGATGAAGAGTGAACCTGTGCTCGCAATATCAACCGGTATGATCGGTCGATACCGGGACGACATTGCCTCTCACCACCGCGAAAGTAAAGGA

GGGCAAGTGTTGACCGCGTTGGATTGCCACATGAAACAATACGGTTTGACAAAGGAAGAGTCGCTATCTAAGTTTGAAGGATTGGTTGAGGAAACATGGA

AGGATACAAACAAGGAATTCGTAGCCACAACTTATGTGCCTAAAGAAATCACCATCACATTCCTTAATTACGCTCGGATGTGTGAGGCCACTTACAACAA

CAATAACGGAGACGCTTATACAGATCCTAATGTTGCCAAGGCAAATGTTACTGCTCTTTTTATTGATGCCATAGTCATTTGA

>MlongTPS13

ATGGCTGCGAACAGCGTCGTAATTAGTTGCCTAAGGGACGTGAGGCCACCTATGACGAAGCACGAGCCAAGCATGTGGGCTGATACTTTTTCTAACTTTT

CTCTTGACGATCAGGAACAACAAAAGTACACAGAAACCATTGAAGCGCTGAAGCAAGAAGCAAGAGGCATGCTTATGGCTTCAACCACGCCTCTCCAACA

AATGACACTAATCGACACTCTCGAGCGTTTGGGATTGTCTTTCCATTTTGAGACGGAGATCGAATACAAAATCGAACTAATCAACGCTGCAGAAGACGGC

GACTTTGATTTGTTCGCTACAGCTCTTCGATTCCGCTTGCTCAGACAGCATCAGCGCCGAGTTTCTTGCGCTCATGTTGGGTTTCGCGACGAAAAAATAT

TACAAGAGGGTGTAAAATTTACGAGGCATCAGTTAATACACATGGAAGGAGAGTTAGAGTCTCCATTTAAAGAGAGAGTGAAGCGAGCTTTGGAGCACCC

TCTTCATAGGGATTTCCCCATTGTCTATGCACGCCTTTTCATCTCCATTTACGAAAAAGATGACTCTAGAGATGAATTACTTCTCAAACTATCCAGAATC

AACTTCAAATTCATGCAGAATTTGTATAAGAAAGAGCTCTCCCAACTCTCCAGGTGGTGGAACACATGGAATCTGAAATCAAAATTACCATACGCAAGAG

ATCGAGTGGTGGAGGCTTATGTTTGGGGAGTAGGTTACCATTACGAACCTCAATACTCATATGTTCGAATGGGACTTACCAAAGGCGTACTAATTTGTGG

AATCATGGACGATACATATGATAATTATGCTACACTCAATGAAGCTCAACTTTTTACTCAAGTCTTAGACAAATGGGATAGAGATGAAGCTGAACGACTC

CCAGAATACATGAAAATCGTTTATCAATTTATTTTGAGTATATATGAAAATTATGAACGTGATGCAGCGAAACTTGGAAAAAGCTTTGCAGCTCCTTATT

TTAAGGAATCCGTGAAACAACTGGCAAGGGCATTCAATGAGGAGCAGAAGTGGGTTATGGAAAGGCAGCTACCGTCATTCCAAGACTACATAAAGAATTC

AGAGAAAACCAGCTGCATTTATACCATGTTTGCTTCTATCATCCCAGGCTTGAAATCTGTTACCCAAGAAACCATTGATTGGATCAAGAGTGAACCCACG

CTCGCAACATCGACTGCTATGATCGGTCGGTACTGGAATGACACCAGCTCTCAGCTTCGTGAAAGCAAAGGAGGGGAAATGCTGACTGCGTTGGATTTCC

ACATGAAAGAATATGGTTTGACGAAGGAAGAGGCGGTATCTAAGTTTGAAGGATTGGTTGAGGAAACATGGAAGGATATAAACAAGGAATTCGTAGTAGC

CACAGCTAATTATAATGTGGGTAGAGAAATTGCCATCACATTCCTCAACTACGCTCGGATATGTGAAGCCAGTTACAGCAAAACTGATGGAGACGCTTAT

TCGAATCCTAATGTTGCCAAGGCAAATGTCGTTGCTCTCTTTGTTGATGCCATAGTCATTTGA

>MlongTPS14

ATGTGTATTCCTATCAAACGAAGTACTAATTTTGTTGATAAGAAACATCAAAAACTGCGGCTTGCTTCTCCCGCTTCATGCACGCTGCAGTCTTCCTCCC

TCAACCTCACTGCTACTCCCACTGTTCCTTGTACTGATGAACTTAGCTCTACACGAAGATCGGGAAATTACAAGCCTACCCTTTGGGATTTCGACCGTAT

TCAATCACTCAACAGTGTTTACACGGAAGAGAAGCACGTGAAAAGGGCTTGTGAGCTAGTTGTGCAAGTGAAGAAGTTGGTTGAGGAAGAACCGAGCTGG

TTTCGACAGTTGGAGTTGATCGATAATCTTCAGAGGCTAGGGCTATCCTATCATTTTGAGGATGAAATCCGCCAAATCTTGAGTTGTATATATTTGGATG

AGAAATACTGCAAGAAAATGGATCTCTACTCAACATCTCTTTCCTTCAGACTCCTCAGACAGCATGGCTTTAAAGTTTCTCAAGAGACTCTGGAGCAAGC

GAGGGTATTTTCGACATACCTTCTGCAGGCAAAGCTTGACGATGGTGGAATAATGGACGAACATCTTTCGGATTTGGTGCGCCATTCACTAGGGTTACCT

CTTCATTGGAGCGTTCAGAGGCCTAACGCCAGATGGTTCATAGATGCTTGTGCGAAGAAGAGATCGGAGAACATGAACCCTATTCTGCTCGAGCTTGCTA

AGTTGGACTTCAACATTGTTCAAGCAGCACATCAACAAGAACTTAAGCATGTCTCAAGATGGTGGGAGGAATCAAAGCTGGCTGAGAAATTGCCGTTTGC

AAGAGATAGGGTGGTAGAGAACTACATATGGAATGTGGGATTGCTATTTGAGCCTCAATATGGATATCCAAGGATCATGACAACCAAGCTTTTCATTTTG

ATTACAGTAATTGATGATATATTTGATGTCTATGGCACCTTGGAAGAAACTCAACTTTTCAACCATACCATTCAAAGATGGGATATTGAAGCATTAGATA

AACTCCCAAAGTACATGCAAATTTGTTATCTGGCATTAGACAGCTTCATCGATGAGACGGCGTACCATGTTCTCAAGGAACAAGACGTTCTCGTCATTCA

AGATTTAAGAAAATCCTGGGCGGATTTGTGCGGAGCATTTGCAAAAGAGGCAGAATGGTACTATACTGGGTATAAACCAACACTAGAAGAATACATTGAA

GTTGCATGGATTTCAATATCAGCTCATACAATATTATCATATGTATTCTTCCTCATATCAAATCCAATAGAGAAGGATGCTGCTGACAGCTTGCGCAATT

ATCATAACGTCATTCGATGTTCGGCAATGGTTCTACGCCTTGCAGATGATCTTGGAACCGGACCGTTCGAGACGAGGAGAGGAGATGTGCCCAAAGCAAT

GGAATGTTACATGAACGATACGGGTGGTTCGATGGAGGAGGCTCGTGAGCATAATGAGGAGGGCGATTTTAAAGGAAGCCTAGGTGAAGATACGAAGGGT

TTGCTACAGCTATATGAAGCTTCCTTCCTTCTGACACACGGTGAAGAGACTCTGGAGCAAGCGAGGGTATTCTCAACAAACCTTCTGCAGAAAAAGCTTG

ATGATGGTGGAATAATGGACGGACATCTTTCGGATTTGGTGCGCCATTCGCTAGGGTTACCTCTTCATTGGAGCATTCAAAGGCCTAACGCAAGATGGTT

CGTAGATGCTTGTGCGAAGAGATGGAACATGAACCCTATTATGCTCGAGCTTGCCAAATTGGACTTCAACATTGTTCAAGCAACACATCAACAAGAACTC

AAGCAAGTCTCAAGATGGTGGGAGGAATCCAAACTAGCTGAAAAGCTGCCGTTTGCGAGAGATAGGGTGGTGGAGAACTATATATGGAATGTGGGGTTCC

TATTAATTGATGATATATTTGATGTCTATGGCACTTTGGAAGAAATTCAACTTTTCAACGACACCATTCAAAGCTTCATCGATGAGATGGCGTACCATGT

TCTCAAGGAACAAGACGTTCTCGTGATTCAAGATTTAAGAAAATCTTGGGCGGATTTGTGCGGAGCATACGCAAAAAAGGCAGAATGGTACTACACTGGG

TATAAACCAACACTAGAGGAATACATGGAAGTTGCATGGATTTCAATATCAGCTCCTACAATGTTAACACATGTATTCTTCCTCATATCAAATCCAATAG

AGAATGAGGCTGCTGACAACTTGCGCAATTATCACAACGTCGTTCGTTGTTCAGCAATGGTTCTACGCCTTGCAAATGATCTAGCAACCGGACCGTTCGA

GACGAGGAGAGGTGATGTGCCAGAAGCAGTTGAGTGCTACATGAACGATACGGGTGGTTCGATGGAGGAGGCTCGTGAGTATGTAAAGTTTATGATAAGA

GAAACATCGAAAGATTCAAATGAAGAAAGATTTAAGAAAAAGTTGCCATTCGCAGAAAACTTTATGAGAATTGCAGCTGATCTTGGAAGGCAGGCGCAGT

ATATGTACCAATATGGAGATGGACATGGAATTAGCAATACACAGATGAAGGAACGCATTTTGGGTCTCATATTTGAACCTATTGTTTAA

>MlongTPS15

ATGTCCACCATTATAATGACCATGACGCTTCCCAACAAACCTACCATTTGTGTTGATAACTTCACAACGAAACATCCAAAACTGCGCCAAGCTTTTCCGG

TTTCCTGCCGCCGCTGGCAGAGTTCCGCCGTCAAATTCAGTGCTATCAATCCTTGTACGGAAGAACTCCAATCTACACGACGATCCGGAAATTACAAACC

TACCCTTTGGGACTTCGATCGTATTCAGTCACTAAACAGTGTTTACACGGAAGAGAAGTATGCGACAAGGGCTTCTGAGCTAGTTGTGGTAGTGAAGAAG

TTGCTTGAGGAAGAATCGAGCTGGTTTGGACAGTTGGAGTTGATCGATGATCTTCAGAGGCTAGGGCTGTCCTATCATTTCAAGGATGAAATCCGCCAAA

TCTTGAGCTCTATATATTTGGATGATAAATACTGCAAGAAAATGGATCTCTACTCAACATCTCTTTGCTTCAGACTCCTCAGACAACATGGCTTTAAAGT

TTCTCAAGATGTGTTTAATTGTTTCAAGAACAAGAAGGGCGATTTTGAGTCAAGCCTAGGTGAAGATACGAAGGGATTGCTAGAGCTGTATGAAGCTTCC

TTCCTTTTGACACACGGTGAAGAGACTCTAGAGCAAGCGAGGGTATTTTCGACAAATCTTCTGCAGAAGAAACTTGATGATGGTGAAATAATGGATGAAC

ATCTTTTGAATTTGGTGCGCCATTCACTAGGGTTACCTCTTCATTGCAGCGTTCAAAGGCCTAACGCGATATGGTTCATAGATGCCTACGCCAAGAGATC

GGACACGAACCCTATTCTGCTCGAGCTCGCTAAATTGGACTTCAATATTGTGCAAGCAGCACATCAACAAGAACTTAAGCATGTCTCAAGATGGTGGGAG

GAATCCAAACTGGCTGAGAAGCTGCCGTTTGCGAGAGATAGGGTGGTGGAGAACTACATATGGAATGTGGGTATGCTGTTTGAGCCTCAATATGGATATC

CAAGAATCATGACAACCAAGCTCTTCATTTTGATTACAGTAATAGATGATATCTTTGATGTCTATGGCACCTTGGAGGAAACCCAGCTTTTCAACAACAC

CATTCAAAGATGGGATACTGAAGGCTTAGATAAACTCCCAGAGTATATGCAAATTTGTTATCTGGCACTAGATAGCTTCATCGATGAGACGGCATACCAT

GTTCTAAAGGAACAAGGCGTTCTCATCATTCAAGATTTAAGAAAATCTTGGGCGGATTTGTGCGCAGCATACGCAAAGGAGGCTGAATGGTACTATACTG

GGTATAAACCAACACTAGAGGAATACATGGAAGTTGCATGGATTTCAATATCAGCTCATACAATACTATCATATGTATTCTTTCTCATATCAAATCCAAT

AGAGAAGGATGCTGCTGAGAACTTGCGTAATTATCACAACGTCATCCGTTGTTCAGCAATGGTTCTACGCCTCGCAGATGATCTCGGAACCGGACCGTTC

GAGATGAGAAGAGGAGATGTGCCGAAAGCAGTGGAGTGCTACATGAATGATACGGGTGCCTCGATGGAGGAGGGTCGTGAGTATGTGAAGTTTATGATAA

GGGAAACATGGAAAGAAACAAATGAAGAAAGTTTTAAAGAAAAGTTGCCATTCTCAGAAATCTTTATGAGAAGTGCAGCTGATCTTGGAAGACAGGCGCA

GTATACGTACCAACATGGAGATGGACATGGAATTAGCAATCGGGAGATGGAGGAACGCATTTTAGGTCTCATATTTGAACCTATTGTTTAG

>MlongTPS16

ATGTGCAGCGTTGTTATGCAAAAGGTGATTCCTAACAACCTAAGTAACGATCGTCACGCCTCACCACTCATCAAATCTACTTCAAAACTGCTCTCCGCCA

CTAAAACTAGTAGACGACGTTTGCGTTGCTCCTCCTCCTCACAAACCGATGCTACCGAAAGACGATCCGGAAACTACAGCCCTTCTCGTTGGAGCGTCGA

TTTTATTCATTCGCTCGACACTGATGTTAAGGAACTGGAGAAGCGCGCGGCGAGGGCTTCAGAGCTAATTACGCAGGTGAAGAAGGAAGTGGAGAAAGAA

AGGGATCAGATTCGACAGCTTGAGTTGATTGATGAGTTGCAGAGCTTGGGTTTGTCCGATCATTTCCGGGATGAGTTCAAAGAAATCTTAAACTCTGTAT

ATCTGGACCATAAATATTACAAGAATCCCGATTCAAAAGATTTGTACTTTACATCTCTCGCTTTCAGGCTCCTCAGAGAACATGGTTTTCAAGTTGCTCA

AGAGGTGTTTGATTGTTTCAAGAATGAGGAGGGTGAGTTCAAAGCAAGCCTTGGCGACGACACAAGAGGATTGCTGCAGCTATACGAAGCTTCTTTCTTG

TTGAAGGAAGGCGAAAACACGCTCGAGTCAGCAAGGGAATTCGCCACCAAATTCCTGCAGGAAAAAGTGAAGGAGGGTGGAGTTGATGACAACCTTTTAA

CAAGAATCGCGTATTCTATGGACATCCCAATTCATTGGAGGATTAAAAGGCCAAATTCAACGGTTTGGATTGATTCATGTAGGAAGAGAGCTGACATGAA

TCCACTAGTGTTGGAGCTGGCCATACTCGACTCCAACCTCCTTCAAGCGCGATATCAAGAAGAACTTAAGCAGGCCTTTAGGTGGTGGAGGAATACATGT

TTTGTGGAGAAGCTTCCTTTCGCTAGGGACAGGCTAGTTGAATGTTATTTTTGGACGACCGGAATCATTGAGCCACGTCAGCATGCGAGTGGTAGGATAA

CGGTGGCCAAAGTTAACGCTCTTATAACTACAATTGATGACGTCTACGATGTGTATGGTACCTTAGAGGAACTTCAACAATTCACGGAGGCCATCCATAA

ATGGGATGTGAGTTCAATCAGCCATCTTCCTACTTACATGCAACTTTGCTTTCTTGCATTGGACAACTTTGTGAACGACACAGCATACGATGTACTGAAA

GAGAAAGGCTTCAACATCATCCCCTATCTGCGTAAAACGTGGAGGGATTTGGTGGATGCATATCTTATAGAAGCCAAATGGTACAACAACGGACATAAAC

CAAATCTGGAAGAGTATCTCAACACCGCCTGGATTTCAATCGGAGCCACAGTTATATTGACTCATGCATTCTTCAGAGTAACAGATTCATTCACAACGGA

GACTGTCGACAGTTTGTACAAATACCATGATTTAGTTCGTTGGTCTGCACTCATTCTGCGCCTCGCCGATGATTTAGGAACCTCTGTGGATGAGGTGAGC

AGAGGCGATGTGCCGAAATCAATTCAGTGCTACATGAATGATAATAATGCTTCAGAGGCGGAGGCGCGAGAGCACGTAAAATGGCTGATAGCAGAGACGT

GGAAGAAGATGAACGAGGAGAGGGTTAAGAAGGATTCTCCATTTTGTGAAGATTTCACCGGATGTGCAGTGGATCTGGGAAGAATGGCGCAGTATATGTA

TCATTATGGAGATGGACATGGGATTCAACACCCTATAATACACCAACAAATGACCACATGCTTGTTCGAGCCTTGTGCGTGA

>MlongTPS17

ATGTGCAGCGTTGCAATGCAAATGGCGATTATTCTTAGCAAGCAAACAAATTATGCCCATAACTCACACATCATTAACTCTTCTCCAATAATCATTCGTC

GATTGCATATTAGTAATGATCGGCGCTCTGGAGGCTACCCTCCTTCCCTTTGGGATTTCCACTCTATTCAAGAGCTAAACACCTCTCAATACAAGGAGGA

GGAGAGGCACCTAAGAAGGGCTGCGGTTTTGATAAGGGAAGTGAAGATGTTGTTGCTGCAGGAAGAAGAAGTTGTTGTTCATCGACAGTTGGAGTTGATT

GATGAGTTGCAGAGGCTGGTTGCAGAGGAACAAAGAGATTTGTACTCCACAGCTCTTGCATTCAGACTACTCAGACAACACGGTTTTCACGTCTCTCAAG

AAGTCTTTGAGTACTATTTCAAGAGTGAAAAGGGTATTGATTTCAAGCCCATCCATGCTGGAGATACCAAAGGATTGTTACAATTGTACCAAGCATCTTT

TCTATCAACACAAGGCGAAGAAACCCTACAATTAGCAAGAGAATATGCCACAAATTTTCTGCAGAAAATACCACTTGACCATCATGAAACTAATGTCGAA

AATCTCTCATCATCATCAATCCGAGACGCCATGGAAATCCCCATTCACTGGAGGGTTCAAATGCCGAACTCAAGATCCTTTATCGATGTCTACAAGAGGA

GAAAGGCCTCGAGGTGGTGGCATAGCATGGGCCTCGTCCAACAGCTTCCCTTTGTGAGGGATAGGATTGTGGAGTGCTACTTTTGGACAACCGGAGTCCT

TGAGCATGACATCTATGATGTTTACGGCACGATTCAAGAGCTTCGACTATTCACTAACGCGATTCAAAGATGGGATATAGAATCGATGAATCAACTACCT

CCTTACATGGAATTGTGTTATCTTGCACTTCACAACTTTGTTAACGAGATGGCTTATGATACCCTCAAGGAAAAAGGTTTCAACTCAATCCCATATCTAC

GGAAAACGTGGGTTGATTTGGTTGAGGCATATATGAGAGAGGCGGAATGGTACCACAACGGTCATAAACCTAGCCTCGAAGAATATATGAAAAATGCTTG

GATATCAATCGGAGGCGTCCCCGATTTTATCCCATATGTTTTCCAGCTAACAAATTCTATAGACGAAGTGACCGTTGAGAGCATGCACGAATACCATGAT

ATAGTTCGTGCATCGTGTACGATTGTTAGGCTTGCTGATGATTTGGGAACATCCTTGGATGAGGTGAAGAGAGGCGACGTACCAAAATCAGTTCAATGTT

ACATGAATGATGAGAAGAATGCTTCTGAGCAAGAGGCACGGGAGCATGTACGATCTCTCATAGAGAAGACATGGAAAACGATGAATGAGGAAATGATGAC

ATCAGCCGATTCTCCATTTTTGAAATATTTTGTGGAAGCTGCTGCTAATCTTGGAAGAATGGCACTCTGCGTCTACCAACATGAATCCGATGGCTTTGGC

ATGCAACATTCAAGGGTTAACAAAATGCTAAGAGGCTTGCTCTTCGACCCCTGTTCATAG

>MlongTPS18

ATGTTGTTGCAGGAAGAAGAAGTTGTTGTTCGACAGTTGGAGTTGATTGATGAGTTGCAGAGGCTGGTTGCAGAGGAACAAAGAGATTTGTACTCCACAG

CTCTTGCATTCAGACTACTCAGACAACACGGTTTTCACGTCTCTCAAGAAGTCTTTAAGTACTACTTCAAGAGTGAAAAAGGTACTGATTTCAAGCCCAT

CCATGGTGAAGATACCAAAGGATTGTTACAAGTGTACGAAGCATCTTTTCTATCAAGACAAGGAGAAGAAACCCTGCAATTAGCAAGAGAATATGCCACA

AACTTTCTGCAGAAAATACCACTAGATCATGAAACTAATGATGAAAATCTCTCATCATCATCAATTCGAGACGCCATGGAAATCCCCAATCACTGGAGGG

TACAAATGCCGAATGCGAGATCCTTCATCGATGCCTACAAGAGGAGAAGTAACATGAATCCAATTGTACTAGAGCTAGCCAAACTTGACTTAACTATTGT

TCAAGCACAGTTTCAACAAGAACTCAAAGAGGCCTCGAGGTGGTGGCATAGCATGGGCCTCGTCCAACAGCTTCCCTTTGTGAGGGATAGGATTGTGGAG

TGCTACTTTTGGACAACCGGAGTCCTTGAGCGTCGTCAACATGGATATGAGAGAATAATGCTCACCAAAAAATATGCTCTTATTACAACTATAGATGACA

TCTATGATGTTTACGGCACGATTCAAGAGCTCCGACTATTCACTAACGCGATTCAAAGATGGGATATAGAATCGATGAATCAACTACCTCCTTACATGCA

ATTGTGTTATCTTGCACTTCACAACTTTGTTAACGAGATGGCTTATGATACCCTCAAGGAAAAAGGTTTCAATTCAATCCCATATCTACGGAAAACGTGG

GTTGATTTGGTTGAGGCATATATGAGAGAGGCGGAATGGTACCACAACGGTCATAAACCTAGCCTCGAAGAATATATGAAAAATGCTTGGATATCAATCG

GAGGCGTCCCGATTTTATCCCATATGTTTTTCCAGCTAACAAATTCCATAGACGAAGTGACCGTTGAAAGCATGCACGAATACCATGATATAGTTCGTGC

ATCGTGTACGATTGTTAGGCTTGCTGATGATTTGGGAACATCCTTGGATGAGGTGAAGAGAGGCGACGTACCAAAATCAGTTCAATGTTACATGAACGAT

GAGAAGAATGCTTCTGAGCAAGAGGCGCGGGAGCATGTACGATCTCTCATAGAGAAGACATGGAAAATGATGAATGAGGAAATGATGACATCAACCGATT

CTCCATTTTCGAAATATTTTGTGGAAGCTGCTGCTAATCTTGGAAGAATGGCACACTGCGTCTACCAACATGAATCCGATGGCTTTGGCATGCAACATTC

AAGGGTTAACAAAATGCTAAGAGGCTTGCTCTTCGACCCCTGTTCATAG

>MlongTPS19

ATGTGCAGCGTTGTTATGCAAAAGGTGATTCCTAACAACCTAAGTAACGATCGTCACGCCTCACCACTCATCAAATCTACTTCAAAACTGCTCTCCGCCA

CTAAAACTAGTAGACGACGTTTGCGTTGCTCCTCCTCCTCACAAACCGATGCTACCGAAAGACGATCTGGAAGCTACAGCCCTTCTCGTTGGAGCGTCGA

TTTTATTCATTCGCTCGACACTGATGTTAAGGAACTGGAGAAGCGCGCGGCGAGGGCTTCAGAGCTAATTACGCAGGTGAAGAAGGAAGTGGAGAAAGAA

AGGGATCCGATTCGACAGCTTGAGTTGATTGATGAGTTGCAGAGCTTGGGTTTGTCCGATCATTTCCGGGATGAGTTCAAAGAAATCTTAAACTCTGTAT

ATCTGGACCATAAATATTACAAGAATCCCGATTCAAAAGATTTGTACTTTACATCTCTCGCTTTCACGCTCCTCAGAGAACATGGTTTTCAAGTTGCTCA

AGAGGTGTTTGATTGTTTCAAGAATGAGGAGGGTGAGTTCAAAGCAAGCCTTGGCGACGACACAAGAGGATTGCTGCAGCTATACGAAGCTTCTTTCTTG

TTGAAGGAAGGCGAAAACACGCTCGAGTCAGCAAGGGAATTCGCCGCCAAATTCCTGCAGGAAGAAGTGAAGGAGGGTGGAGTTGATGACAACCTTTTAA

CAAGAATCGCGTATTCTATGGACATCCCAATTCATTGGAGGATTAAAAGGCCAAATTCAACGGTTTGGATTGATTCATGTAGGAAGAGACCCCACATGAA

TCCACTGGTGTTGGAGCTGGCCATACTCGACTCCAACCTCCTACAAGCGCGATATCAAGAAGAACTCAAGCAGGCCTTTAGGTGGTGGAGGAATACATGT

TTTGTGGAGAAGCTTCCTTTCGCTAGGGACAGGCTAGTTGAATGTTATTTTTGGACGACCGGAATCATTGAGCCACGTCAGCATGCGAGTGGTAGGATAA

CGGTGGCCAAAGTTAACGCTCTTATAACTACAATTGATGACGTCTACGATGTGTATGGTACCTTAGAGGAACTTCAACAATTCACGGAGGCCATCCATAA

ATGGGATGTGAGTTCAATCAGCCATCTTCCTACTTACATGCAACTTTGCTTTCTTGCATTGGACAACTTTGTGAACGACACAGCATACGATGTACTGAAA

GAGAAAGGCTTCAACATCATCCCCTATCTGCGTAAAACGTGGAGGGATTTGGTGGATGCATATCTTATAGAAGCCAAATGGTACAACAACGGACATAAAC

CAAATCTGGAAGAGTATCTCAACACCGCCTGGATTTCAATCGGAGCCACAGTTATATTGACTCATGCATTCTTCAGAGTAACAGATTCATTCACAACGGA

GACTGTCGACAGTTTGTACAAATACCATGATTTAGTTCGTTGGTCTGCACTCATTCTGCGCCTCGCCGATGATTTAGGAACCTCTGTGGATGAGGTGAGC

AGAGGCGATGTGCCGAAATCAATTCAGTGCTACATGAATGATAATAATGCTTCAGAGGCGGAGGCGCGAGAGCACGTAAAATGGCTGATAGCAGAGACGT

GGAAGAAGATGAACGAGGAGAGGGTTAAGAAGGATTCTCCATTTTGTGAAGATTTCATCGGATGTGCAGTGGATCTGGGAAGAATGGCGCAGTATATGTA

TCATTATGGAGATGGACATGGGATTCAACACCCTATAATACACCAACAAATGACCACATGCTTGTTCGAGCCTTGTGCGTGA

>MlongTPS20

ATGTGCAGCGTTGTTATGCAAACGGTGATTCCTAGCAAGCTAAGTAATGATGGTCACACCTCATCATCACTCATCAAATCTTCTTCAAAACTGCTCTCCA

CCACTAAAACTAGTAGACGTTTGCGCTGCTCCTCCTCGCAAATCGTTGCTACTGAAAGGCGATCCGGAAACTACAGCCCTTCTCGTTGGAGCGTCGACTT

TATTCAGTTACTCCACACCGATGATAAGGAACTCGAGAAGTGCACGACAAGGGCTTCAGAGCTAATTACGCAGGTGAAGAAGGAAGTGGAGAAAGAAAGG

AATCCGATTCGACAGCTTGAGTTGATTGATGAGTTGCAAAGGTTGGGTTTGTCCGATCATTTCCAGGATGAGTTCAAAGAGATCTTGAACTCTGTATATC

TGGAACTGGACCATAAATATTACAAGAATCCCAATTCAAAAGAGAGAGGAGATTTGTACTTCACATCTCTCGCTTTCAGGCTCCTCAGAGAACATGGTTT

TCAAGTTGCTCAAGAGGTATTTGATTGTTTCAAGAACGAGGAGGGTGAGTTCAAAGCAAGTCTTAGCGACGACACCGGAGGATTGCTGCAGCTATACGAA

GCTTCTTTCCTGTTGACTGAAGGGGAAAACACGCTTGAGTCAGCAAGGGAATTCGCCGCCAAATTCCTGCAGGAAAAAGTGAAGGAGGGTGGAGTTGATG

GCAACCTTTTAACAAGAATCGAGTATTCTATGGACATCCCAATTCATTGGAGGATTAAAAGGCCAAATGCAACGGTTTGGATTGATTCATATAGGAAGAG

AGCTGACATGAATCCACTAGTGTTGGAGCTGGCCATACTCGACTCCAACCTCCTTCAAGCACGATATCAAGAAGAACTCAAGCAGGCTTTTAGGTGGTGG

AAGAATACATGTATTGCGGAGAAGCTTCCTTTCGCGAGGGACAGGCTAGTGGAATGTTATTTTTGGACATTTAGAGGTGTGGAGCCACGTCAGCATACGA

GTGCTAGGATATATGGGACCAAAGTTAACGCTCTTATAACTACAATTGATGATGTCTACGATGTTTATGGTACCTTAGAGGAACTTCAACAATTCACGGA

GGCCATCCGAAAATGGGATGTGAGTTCAATCCGCCATCTTCCTAAGTACATGCAACTATGCTTTCTTGTATTGGATAACTTTGTGAACGACGCAGCATAC

CATGTTATGAAAGAGAAAGGCTTCAACATCATCCCCTATATGCGAAAATCGAGGATCCGCCCAAAATTTGGAGAACTGGTGGCGGAGAATGCAGTGGTGG

TGTTTCGCTCGGAAGGGGTGTTGTATGTGCCACGTGGTCAAGCTCTTGCTGTACAGATCTGA

>MlongTPS21

ATGTGCAGCGTTGCAATGCAAATGGCGATTATTCTTAGCAAGCAAACAAATTATGCCCATAACTCACACATCATTAACTCTTCTCCAATAATCATTCGTC

GATTGCATATTAGTAATGATCGGCGCTCTGGAGGCTACCCTCCTTCCCTTTGGGATTTCCACTCTATTCAAGAGCTAAACACCTCTCAATACAAGGAGGA

GGAGAGGCACCTAAGAAGGGCTGCGGTTTTGATAAGGGAAGTGAAGATGTTGTTGCTGCAGGAAGAAGAAGTTGTTGTTCATCGACAGTTGGAGTTGATT

GATGAGTTGCAGAGGCTGGTTGCAGAGGAACAAAGAGATTTGTACTCCACAGCTCTTGCATTCAGACTACTCAGACAACACGGTTTTCACGTCTCTCAAG

AAGTCTTTGAGTACTATTTCAAGAGTGAAAAGGGTATTGATTTCAAGCCCATCCATGCTGGAGATACCAAAGGATTGTTACAATTGTACCAAGCATCTTT

TCTATCAACACAAGGCGAAGAAACCCTACAATTAGCAAGAGAATATGCCACAAATTTTCTGCAGAAAATACCACTTGACCATCATGAAACTAATGTCGAA

AATCTCTCATCATCATCAATCCGAGACGCCATGGAAATCCCCATTCACTGGAGGGTTCAAATGCCGAACTCAAGATCCTTCATCGATGTCTACAAGAGGA

GAAATGACATCTATGATGTTTACGGCACGATTCAAGAGCTTCGACTATTCACTAACGCGATTCAAAGATGGGATATAGAATCGATGAATCAACTACCTCC

TTACATGGAATTGTGTTATCTTGCACTTCACAACTTTGTTAACGAGATGGCTTATGATACCCTCAAGGAAAAAGGTTTCAACTCAATCCCATATCTACGG

AAAACGCTAACAAATTCTATAGACGAAGTGACCGTTGAGAGCATGCACGAATACCATGATATAGTTCGTGCATCGTGTACGATTGTTAGGCTTGCTGATG

ATTTCGGAACATCCTTGGATGAGGTGAAGAGAGGCGACGTACCAAAATCAGTTCAATGTTACATGAATGATGAGAAGAATGCTTCTGAGCAAGAGGCACG

GGAGCATGTACGATCTCTCATAGAGAAGACATGGAAAACGATGAATGAGGAAATGATGACATCAGCCGATTCTCCATTTTTGAAATATTTTGTGGAAGCT

GCTGCTAATCTTGGAAGAATGGCACTCTGCGTCTACCAACATGAATCCGATGGCTTTGGCATGCAACATTCAAGGGTTAACAAAATGCTAAGAGGCTTGC

TCTTCGACCCCTGTTCATAG

>MlongTPS22

ATGATGAGAAGTGGAACATTTCTGTTTATACTAGTTAATGATCGGCGCTCTGGAGGCTACCCTCCTTCCCTTTGGGATTTCCACTCTATTCAAGCGCTAA

ACACCTCTCAATACAAGGAGGAGGAGAGGCACCTAAGAAGGGCTGCGGTTTTGATAAGGGAAGTGAAGATGTTGTTGCAGGAAGAAGAAGTTGTTGTTCG

ACAGTTGGAGTTGATTGATGAGTTGCAGAGGCTGGGTATATCTTGCCATTTTGATGAAGAAATCAAACAAATCTTGAATTCTTTTTACTACAACAATTAC

TACGATCATGCAGTTGCAGAGGAACAAAGAGATTTGTACTCCACAGCTCTTGCATTCAGGCTACTCAGACAACACGGTTTTCACGTCTCTCAAGAAGTCT

TTAAGTACTACTTCAAGAGTGAAAAAGGTACTGATTTCAAGCCCATCCATGGTGAAGTTACCAAAGGATTGTTACAATTGTACGAAGCATCTTTTCTATC

AACACAAGGCGAAGAAACCCTACAATTAGCAAGAGAATATGCCACAAATTTTCTGCAGAAAATACCACTTGATCATCATGAAACTAATGAAAATCTCTCA

TCATCATCAATTCGAGACGCCATGGAAATCCCAATTCATTGGAGGGTACAAATGCCGAACGCAAGATCCTTCATCGATGCCTACAAGAGGAGAAGTAACA

TGAATCCAATTGTACTAGAGCTAGCCAAACTTGACTTAATTATTGTTCAAGCACAGTTTCAACAAGAACTCAAAGAGGCCTCGAGGTGGTGGCATAGCGT

GGGCCTCGTTCAACAGCTTCCCTTTGTGAGGGATAGGATTGTGGAGTGCTACTTCTGGACAACCGGAGTCCTTGAGCGTCGTCAACATGGATATGAGAGA

ATAATGCTCACCAAAATAAATGCTCTTATAACAACTATAGATGACATCTATGATGTTTACGGCACCATTCAAGAGCTCCGACTATTCACTAATGCGATTC

AAAGGTGGGTTGATTTGGTTGAGGCATATATGAGAGAGGCGGAATGGTACCACAACGGTCATAAACCTAGCCTCGAAGAATATATGAAAAATGCTTGGAT

ATCAATCGGAGGCGTCCCGATTTTATCCCATATGTTTTTCCAGCTAACAAATTCTATAGACGAAGTGACCGTTGAGAGCATGCACGAATACCATGATATA

GTTCGTGCATCATGTACGATTGTTAGGCTTGCTGATGATTTGGGAACATCCTTGGATGAGGTGAAGAGAGGCGACGTACCAAAATCAGTTCAATGTTACA

TGAACGATGAGAAGAATGCTTCTGAGCAAGAGGCGCGGGAGCATGTACGATCTCTCATAGAGAAGACATGGAAAATGATGAATGAGGAAATGATGACATC

AACCGATTCTCCATTTTCGAAATATTTTGTGGAAGCTGCTGCTAATCTTGGAAGAATGGCACACTGCGTCTACTAA

>MlongTPS23

ATGTGTACCATCCTTAGCAAGCCTCAACTAAATCTTTTCCACACCAAAAACAAGAGATCGGCTTCAATTAATCGCCCATGGCCATGGAGTCTCTCTCCTA

CTTCTAATTCCTCCTCTGATCTCCCCATGCCTTGTTCTATCTCCTCAAAACTACATACCATCCAAACAATCCAACGTCGTTCTGGAAACTACGAGCCTTC

ACTCTGGGATTTCGATTACATTCAGTCTCTCGACACTCACCGCTATAAGGAGGAGAAGCACTTGAGTAGGGAAGAAGAGTTGATTGTGCAGGTGAAGATG

CTGCTGGGGAAAGAAATGGAGGCGGTGAAGCAGTTGGAACTCCTCAGGCATCATGGTTTTGATGTTTCACAAGAAATATTTGATTGTTTCAAGAACGAGG

AGGGAAGTGATTTCGAGAAAACCCTAATTGGAGAAGATACAAAAGGGATATTACAACTTTTTGAAGCATCTTTCCTTTTGAGGGAAGGTGAAGATACACT

TGAGCTAGCTAGAAAATTCTCCACCAAATATTTGCAGAAAAGAGTTGATGAGGGAATTCTAAATGATGATGATAATAATAATAATATATTATCATGGATT

CGTCATTCTTTGGATCTCCCTCTTCATTGGAGGATTCAAAGACTCGAGGCAAGATGGTTCTTAGATGCTTACTCAAGGAGGAAAGACATGAATCCACTTA

TTTTCGAGCTCTCGATACTCGACTTCAATATCATTCAAGCAACACATATACTTGAACTCAAAGAGGTCTCAAGGGTGGTGGAAAGCTTCTTCTGGGCGGT

TGGGCTGTTTGAGGGCCATGAATATGGATATCAAAGAAAAATGGCTGCCTCCATTATTATTCTAATCACAGCCATTGACGATGTTTACGATGTGTACGGT

ACATTACACGAACTGCAGCTCTTCACAGACACCATTCGAAGATGGGATACCGCATCAATAGACCAGCTTCCATATTACATGCAACTATGCTATTTGGCGC

TCTACAACTATGTTTCCAATCTGGCTTACGATATTCTTAAAGACCGCCGTTTCAACACTATCCCATATTTGCATAAATCGTCTGAGCTGAAGAGAGGCGA

CGTGCCGAAAGCAATCCAGTGTTACATGAAGGATAAAAATTGTTCGGAGGAAGAGGCGCGGGCTCACGTGAGGGGGATGATCGGAGAGGTGTGGAAGGAG

ATGAACACGGCCATGGCGAAGTCCGATGATGATTGCCCATTTACGGAACAAGTGGTGGTGGCTGCAGCTAATCTGGGAAGAGCTGCACAGTTTATTTATA

TGGAGGGAGATGGCCATGGACATTCCCAAATACATGGACAGATGAGAAGCCTGCTGTTCCACCCATATATATGA

>MlongTPS24

ATGTCAATTATTAGCATGCATGCATCGATCCTCAGTAAGCAAGGAAATAACCCCCACAAGTTGGACATGAGATCATCAGCTCCAAAACTGATGAGGCGCG

TGTCTTGCACTCGCCTCCGGCCTTGTTGCTCCGCCTCTTCACAACCGCAGGTTGACGGAACCCGACGTTCCGGAAACTACCAGCCTTCACTCTGGGATTT

CGATTACATTCAATCTCTCAACACTCATCACTACAAGGAGGAGAGGCAGTTGAATAGGGAAGAAGAACTGATTATTCAAGTGAAAAAGATGCTTTTGGGG

GAAAAAATGGAGGCAGTGAAGCAGTTGGAATTGATTGATGACTTGAAAAATCTTGGATTATCTTATTTTTTTCAAGATGAGATAAAAAAGATCTTAAGTT

GTATATATAATGAGCACAATTTTTTCCAAAATAATAAAGTAGGGGATTTGCATTTCACTGCTCTAGGGTTCAGACTCCTCAGGCATCATGGTTTTGATGT

TTCACAAGAAATATTTGATTGTTTCAAGAATGAGGAGGGAAGTGATTTCGAGAAAACCCTAATTGGAGAAGATACAAAAGGGATGTTACAACTTTATGAA

GCATCTTTCCTTTTGAGGGAAGGTGAAGATACACTTGAGCTAGCTAGAAAATTCTCCACCAAATATCTGCAGAAAAGAGTTGATGAGGGAATTAGAAATG

ATGATAATAATCTATTATCATGGATTCGTCATTCTTTGGATCTCCCTCTTCATTGGAGGATTCAAAGGCTAGAAGCAAGATGGTTCTTAGATGCTTACTC

AAGGAGGAAAGACATGAATCCACTTATTTTCGAGCTCGCGATACTCGACTTCAATAATATTCAAGCAACACAACTAGAGGAGCTCAAAGAGGTCTCACGG

TGGTGGAATAGTTCTTGCCTCGCTGAGAAACTCCCATTCGTGAGGGATAGAATAGTGGAATGCTACTTTTGGGCGCTAGGGCTCTTTGAGCCTCATGAAA

ATGGATACCAAAGAAAGAAAGCCGCTATTATTATCACTTTTGTTACAATTATAGACGATGTTTACGATGTCTACGGTACATTAGACGAACTGCAGCTATT

CACCAATACAATTCAAAGATGGGACACTGATACAATAAACCAACTTCCTTATTACATGCAAGTGTGCTATTTGGCACTCTACACCTTTGTTTCCGAAATG

GCTTACGATATTCTCAAAGAGCAAGGTTTCAACAGCATCCCATATTTACAGAAATCGTGGGTGAGTTTGGTTGAAGGGTTTTTCAAAGAGGCACAATGGT

ACTACAAAGGATACACGCCAACCCTAGAAGAATATCTTAACAACGCCAAGATTTCAATTTCTTCTCCTACAATAATATCCCAAATTTATTTTACGCTACC

AAACTCGACTGAGAAAACCGCTATTGAAAGCTTGTACGAGTACCACGACATACTTTGTCTCTCAGGAATGATTCTGAGGCTTGCTGATGATCTGGGTACG

ACACAGTTTGAGCTGAAGAGAGGGGACGTGCCGAAAGCAATCCAGTGTTACATGAAGGATAGAAATAGTACGGAGAAAGAGGCGCAGGAGCACGTGAGGT

TTCTGATTCGAGAGGCGTGGAAGGAAATGAACACGGCCATGGCGGATTCCGATTGTCCGTTCTCGGAAGAACTGGTGGCGGCTGCAGCTAATCTGGGAAG

AGCGGCGCAGTATATATATCTAGAAGGAGATGGCCATGGCGTTCAACACTCAGAAATACATAAACAAATGGGAGGCCTTATTTTCGAGCCATATGCATGA

>MlongTPS25

ATGTGTACTATTATTAACGTAAATCACGCTAAAGTAAATCTTTTCCACACCAAAAACAAGAGATCAGCTTCCATTAATCTCCCATGGCCATGGACTCTCT

CTCCTACTTCTAACTCCTCCTCTCGCCCCATCACTTGTTCTATCTCCTCAAAACTACATACCACCGAAACAATCCGACGTCGTTCTGGAAACTACGAGCC

TTCACTCTGGGATTTCAATTACATCCAATCTCTCAACACTCATCACTATAAGGAGGAGAAGCTCTTGAATCGGGAGGAAGACCTGATCGTGGAGGCAAGA

GGTATTGGGGAAAATGAGGCAGTGAAGCAGTTGGAGTTGATTGATGACTTGCAGAATCTGGGATTGTCTTATTTTTTTCGAGACGAGATCAAAAATGTGT

TAGGTTTCATATATGTTGAGCACGGATGTTTTAGAAATAATAATCAAGTATGGGATTTGTATTTCACAGCTCTTGGATTCAGACTCCTCCGGCAGCATGG

TTTCAACGTTACACAAGGAGTATTTGATTGCTTCAAGAACGAGGATGGTAGTGATTTCGAGAAAACCCTAATCGGGGAAGATACTAAAGGAGTGTTGCAA

CTTTACGAAGCATCATTCCTTTTGAGAGAAGGTGAAGATACACTGGAGGTAGCTAGAAAATTCTCAACCGAATTTCTCGAGGAAAAACTCGAAGCCGGAA

TCGATGATGATAATCTATCATCATCGATTGGCGATTCTTTGGAGATCCCTCTTCACTGGAGGATTCAAAGACTAGAGGCAAGATGGTTCTTAGATGCTTA

TTCAAGGAGGAAAGACAAGAATCCACTTATTTTCGAGCTCGCAAAACTCGACTTCAATATTATTCAAGCAACGCAGCAAGAAGAACTCAAAGATCTCTCA

AGGTGGTGGAATGATTCAAGCCTACCTCAAAAACTCCCATTTGTGAGGGATAGGCTGGTGGAAAGCTACTATTGGGCCCTTGGGATGTTTGAGGCTCACA

AATTTGGATATGAAAGAAAAGCTGCTGCAAAGATTATAACCCTAATTACAGCTCTTGATGATGTTTATGATATCTATGGTACACTCGACGAGCTCCAACT

ATTTACCCACGTCATTCGAAGATGGGATACTGAATCAGCCGCCCAACTTCCTTATTACTTGCAATTATTCTATTTCGTACTATACAACTTTGTTTCCGAG

GTGGCGTACCACATTCTAAAAGAAGAGGGTTTCATCAGCATCCCATATCTACAGAGAGCGTGGGTGGATTTGGTTGAAGGATATTTACAAGAGGCAAAGT

GGTACTGCACTAAATATACACCAACCATGGAAGAATATTTGAACTATGCCAGCATCACAATAGGGGCTCCTGCAGTAATATCCCATGTTTATTTTATGCT

AGCCAAATCGAAAGAGAAACCGGTGATCGAGAGTTTTTACGAATTCGACGAAATAATTCGCCTTTCTGGGATGCTCGTGAGGCTTCCCGATGACCTAGGA

ACACTACCGAATGCAACACGGGAAGAAGCAGAAGAACACGTGAGGTTTATGATTGGGGAGGCGTGGAAGCAGATGAACACAACTATGGCGGCGAATTCTG

ATTTGAGAGGTGATGTGGTTATGGCTGCAGCTAATCTTGGAAGGGATGCACAGTTTATGTATCTCGACGGAGACGGTAACCACTCTCAATTACAGCACCG

GATTGCGAACTTGCTGTTCAAACCATATGTCTGA

>MlongTPS26

ATGTGTACCATCCTTAGCAAGCCTCAACTAAATCTTTTCCACACCAAAAACAAGAGATCAGCTTCAATTAATCGCCCATGGCCATGGAGTCTCGCTCTTG

CTTCTAATTCCTCCTCTGATATCCCCATGCCTTGTTCAATCTCCTCAAAACTACATACCATCGAAACAATCCAACGTCGTTCTGGAAACTACGAGCCTTC

ACTCTGGGATTTCGATTACATTCAGTCTCTCGACACTCACCGCTATAAGGTGAAGATGCTGCTGGGGAAAGAAATGGAGGCGGTGAAGCAGTTGGAGTTG

ATTGATGACTTGAAAAATCTTGGATTATCTTATTTTTTTCAAGACGAGATAAAAAAGATCTTAAGTTGTATATATAATGAACACAATTTTTCCCAAAATA

ATAAAGTAAGGGATTTGCATTTCACTGCTCTAGGGTTCAGACTCCTCAGGCATCATGGTTTTGATGTTTCACAAGAAATATTTGATTGTTTCAAGAACGA

GGAGGGTAGTGATTTCGAGAAAGCCCTAATTGGTGAAGATATGAAAGGAATATTACAACTTTACGAAGCATCTTTCCTTTTGAGGGAAGGTGAAGATACA

CTTGAGCTAGCTAGAAAATTCTCCACCAAATATTTGCAGAAAAGAGTTGATGAGGGAATTATAAATGATGATAATAATAATAATATATTATCATGGATTC

GTCATTCTTTGGATCTCCCACTTCATTGGAGGATTCAAAGGCTAGAAGCAAGATGGTTCTTAGATGCTTACTCTACGAGGAAAGACATGAATCCACTTAT

TTTCGAGCTCTCGATACTCGACTTCAATAATATTCAAGCAACACACATACTTGAACTCAAAGAGGTCTCAAGGTGGTGGAACAATTCATGTTTGGCTGAA

AAACTCCCCTTCGTGAGGGATAGGGTGGTGGAAAGCTTCTTCTGGGCGGCTGGGCTGTTTGAGGGTCATGAATATGGATATCAAAGAAAAATGGTTGCCT

CCATTATTATTCTAATCACAGCCATTGACGATGTTTACGATGTGTACGGTACATTAGGCGAACTGCAGCTGTTCACAGACACCATTCGAAGATGGGATAC

CGAGTCAATAGACCAGCTTCCATATTACATGCAACTATGCTATTTGGCGCTCTACAACTATGTTTCCAATCTGGCTTACGATATTCTTAAAGACCGCCGT

TTCAACACTATCCCATATTTGCATAAATCGGAGGCGGAGTGGTACGAGAGTGGATACACACCAACCCTAGAAGAGTATCTGAGCAATGCCAAGATTTCAA

TAGGCTCTCTTACAATATTATTACAAGTTGAATTATCATTACAAAAGTCAACTCTTGATCGCACCGCATTCGATCTCCGCCACAAAATACTTTATCTTTC

TGCCCTAGTTTCGAGGCTTGCTGATGATCTAGGAACAGCACCGTCTGAGCTGAAGAGGGGCGACGTGCCGAATGCAATCCAGTGTTACATGAAGGATAAA

AATTGTTCGGAGGAAGAGGCGCGGGCTCACGTGAGGGGGATGATCGGAGAGGTGTGGAAGGAGATGAACACGGCCATGGCGGTGTCCGATGATGATTGCC

CGTTTACGGAACAAGTGGTGGAGGCTGCAGCTAATCTGGGAAGAGCTGCACAGTTTATTTATATGGAGGGAGATGGCCATGGACATTCCCAAATTCATGA

ACAGATGAGAAGCCTGCTGTTCCACCCATATATATGA

>MlongTPS27

ATGTCAATTATTAGCATGCATGCATCGATCCTCAGTAAGCAAGGAAATAACCCCCACAAGTTGGACATGAGATCATCAGCTCCAAAACTGACGAGGCGCG

TGTCTTGCACTCGCCTCCAGCCTCGTTGCTCCGCCTCTTCACAACCGCAGGTTGACGGAACCCGACGTTCCGGAAACTACCAGCCTTCACTCTGGGATTT

CGATTACATTCAATCTCTCAACACTCATCACTACAAGGAGGAGAGGCAGTTGAATAGAGAAGAAGAACTGATCATTCAAGTGAAAAAGATGCTTTTGGGG

GGGAAAATGGAGGCAGTGAAGCAGTTGGAGTTGATTGATGACTTGAAAAATCTTGGATTAACTTATTTTTTTCAAGAGGAGATAAAAAATATCTTAAGTA

GTATATATAATGAGCATAAATTTTTCCAAAATAATAAAGTAGGGGATTTGCACTTCACTGCTCTAGTTAGGCAAGGTGAAAATACACTTGAGCTAGCTAG

AAAATTCTCCACCAAATATCTGCAGAAAAAGGTTGATGAGGGAATTATAAATGATGATAATAATCTATTATCATGGATTCGTCATTCTTTGGATCTCCCT

CTTCATTGGAGGATTCAAAGGCTAGAAGCAAGATGGTTCTTAGATGCTTATTCAAGGAGGAAAGACATGAATCCACTTATTTTTGAGCTCTCGATACTCG

ACTTCAATAATATTCAAGCAACACAACTAGAGGAGCTCAAAGAGGTCTCAAGATGGGACACTGATTCAATAAACCAACTTCCTTATTACATGCAAGTGTG

CTATTTGGCACTCTACACCTTTGTTTCCGAAATGGCTTACGATATTCTCAAAGAGCAAGGGTTTTTCAAAGAGGCACAATGGTACTACAAAGGATACACG

CCAACCCTAGAAGAATATCTTAACAATGCCAAGATTTCAATTTCTTCTCCTACAATAATATCCCAAATTTATTTTACGCTACCAAACTCGACTGAGAAAA

CCGCTATCGAAAGCTTGTACGAGTACCACGACATACTTTGTCTCTCAGGAATGATTCTGAGGCTTGCTGATGATCTGGGTACGACACAGTTTGAGCTGAA

GAGAGGGGACGTGCCGAAAGCAATCCAGTGTTACATGAAGGATAGAAATAGTACGGAGCAAGAGGCGCAGGAGCACGTGAGGTTTCTGATTCGAGAGGCG

TGGAAGGAAATGAACACGGCCATGGCGGATTCCGATTGTCCGTTCTCGGAAGAATTGGTGGCGGCCGCAGCTAATCTGGGAAGAGCGGCGCAGTATATAT

ATCTAGAAGGAGATGGCCATGGCGTTCAACACTCAGAAATACATAAACAAATGGGAGGCCTTATTTTCGAGCCATATGCATGA

>MlongTPS28

ATGGCTCTCAAAGTGTTTAGTGTTGTAACTCAAATGGCGATTCCTAGCAAGCTAACGAGATGTCTTCAACCCTCACACTTGAAATCCTCTCCAAAATTGT

TATCTAGCACTAACAGTAGTAGTCGGTCTCGCCTCCGTGTGTATTGCTCCTCCTCGCAACTCACTACTGAGAGACGATCCGGAAACTACAACCCTTCTCG

TTGGGATGTCGAATTCATCCAATCCCTCCACAGTGATTATGAGGAGGACAAACACGCGATTAGGGCTTCTGAGCTGGTCACTTTGGTGAAGATGGAATTG

GAGAAAGAAACGGATCATATTCGACAACTTGAGTTGATCGATGACTTGCAGAGGATGGGGCTGTCCGATCATTTCCAAAATGAGTTCAAAGAAATCTTGT

CCTCTATATATCTCGACCATCACTATTACAAGAACCCTTTTCCAAAAGAAGAAAGCGATCTCTACTCCACATCTCTTGCATTTAGGCTCCTCAGAGAACA

TGGTTTTCAAGTCGCACAAGAGGTATTCGACAGTTTCAAGAACGAGGAGGGTGAGTTCAAAGAAAACCTTAGCGACGACACCAGAGGATTGTTGCAACTG

TATGAAGCTTCCTTTCTGTTGACGGAAGGCGAAACCACGCTCGAGTCAGCGAGGGAATTCGCCACCAAATTTTTGGAGGAAAGAGTGAACGAGGGTGGTG

TTGATGGCGACCTTTTAACAAGAATCGCATATTCTTTGGACATCCCACTTCATTGGAGGATTAAAAGGCCAAATGCACCTACGTGGATCGAATGGTATAG

GAAGAGGCCCGACATGAATCCAGTAGTGTTGGAGCTTGCCATACTCGACTTAAATATTGTTCAAGCACAGTTTCAAGAAGAGCTCAAAGAATCCTTCAGG

TGGTGGAGAAATACTGGGTTTGTTGAGAAGCTGCCCTTCGCAAGGGATAGACTGGTGGAATGCTACTTTTGGAATACTGGGATCATCGAGCCACGTCAGC

ATGCAAGTGCAAGGATAATGATGGGCAAAGTCAACGCTCTGATTACGGTGATCGATGATATTTATGATGTCTACGGCACCTTAGAAGAACTCGAACAATT

CACTGACCTCATTCGAAGATGGGATATAAACTCAATCGACCAACTTCCCGATTACATGCAACTGTGCTTTCTTGCACTCAACAACTTCGTCGATGATACA

TCGTACGATGTTATGAAGGAGAAAGGCGTCAACGTTATACCCTACCTGCGGCAATCGTGGGTGGATTTGGCGGATAAGTATATGGTAGAGGCACGGTGGT

TCTACGGCGGACACAAACCAAGTTTGGAAGAGTATTTGGAGAACTCATGGCAGTCGATAAGTGGGCCCTGTATGTTAACGCACATATTCTTCCGAGTAAC

AGATTCGTTCACAAAGGAGACCGTCGACAGTTTGTACAAATACCACGATTTAGTTCGTTGGTCATCCTTCGTTCTGCGGCTTGCTGATGATTTGGGAACC

TCGGTGGAAGAGGTGAGCAGAGGCGATGTGCCGAAATCACTTCAGTGCTACATGAGTGACTACAATGCATCGGAGGCGGAGGCGCGGAAGCACGTGAAAT

GGCTGATAGCGGAGGTGTGGAAGAAGATGAATGCGGAGAGGGTGTCGAAGGATTCTCCATTCGGCAAAGATTTTATAGGATGTGCAGCTGATTTAGGAAG

GATGGCGCAGTTGATGTACCATAATGGAGATGGGCACGGCACACAACATCCTATAATACATCAACAAATGACCAGAACCTTATTCGAGCCCTTTGCATGA

>MlongTPS29

ATGGCTTTCAAAGTGTTTAGTGTTGCAACTCAAATGGCGATTCCTAGCAACCTAACGACATGTCTTCAACCCTCACACTTGAAATCTTCTCCAAAATTGT

TATCTAGCACTAACAGTAGTAGTCGGTCTCGCCTCCGTGTGTATTGCTCCTCCTCGCAACTCACTACTGAAAGACGATCCGGAAACTACAACCCTTCTCG

TTGGGATGTCGACTTCATCCAATCGCTTCACAGTGACTATAAGGAGGACAAACACGCGATTAGGGCTTCTGAGCTGGTCACTTTGGTGAAGATGGAATTG

GAGAAAGAAACGGATCATATTCGACAACTTGAGTTGATCGATGACTTGCAGAGGATGGGGCTGTCCGATCATTTCCAAAATGAGTTCAAAGAAATCTTGT

CCTCTATATATCTCGACCATCACTATTACAAGAACCCTTTTCCAAAAGAAGAAAGGGATCTCTACTCCACATCTCTTGCATTTAGGCTCCTCAGAGAACA

TGGTTTTCAAGTCGCACAAGAGGTATTCGACAGTTTCAAGAACGAGGAGGGTGAGTTCAAAGAGAGCCTTAGCCACGACACCAGAGGATTGTTGCAACTG

TATGAAGCTTCCTTTCTGTTGACGGAAGGTGAAACCACGCTCGAGTCAGCGAGGGAATTCGCCACCAAATTTTTGGAGGAAAGAGTGAACGAGGGTGGTG

TTGATGGCGACCTTTTAACAAGAATCGCATATTCTTTGGACATCCCACTTCATTGGAGGATTAAAAGGCCAAATGCACCTGTGTGGATCGAATGGTATAG

GAAGAGGCCCGACATGAATCCAGTAGTGTTGGAGCTTGCCATACTCGACTTAAATATTGTTCAAGCACAGTTTCAAGAAGAGCTTAAAGAATCCTTCAGG

TGGTGGAGAAATACTGGGTTTGTTGAGAAGCTGCCCTTCGCAAGGGATAGACTGGTGGAATGCTACTTTTGGAATACTGGGATCATCGAGCCACGTCAGC

ATGCAAGTGCAAGGATAATGATGGGCAAAGTCAACGCTCTGATTACGGTGATCGATGATATTTATGATGTCTATGGCACCTTAGAAGAACTCGAACAATT

CACTGACCTCATTCGAAGATGGGATATAAACTCAATCGACCAACTTCCCGATTACATGCAACTGTGCTTTCTTGCACTCAACAACTTCGTCGATGATACA

TCGTACGATGTTATGAAGGAGAAAGGCGTCAACGTTATACCCTACCTGCGGCAATCGTGGGTGGATTTGGCGGATAAGTATATGGTAGAGGCACGGTGGT

TCTACGGCGGACACAAACCAAGTTTGGAAGAGTATTTGGAGAACTCATGGCAGTCGATAAGTGGGCCCTGTATGTTAACGCACATATTCTTCCGAGTAAC

AGATTCGTTCACAAAGGAGACTGTCGACAGTTTGTACAAATACCACGATTTAGTTCGTTGGTCATCCTTCGTTCTGCGGCTTGCTGATGATTTGGGAACC

TCGGTGGAAGAGGTGAGCAGAGGCGATGTGCCGAAATCACTTCAGTGCTACATGAGTGACTACAATGCATCGGAGGCGGAGGCGCGGAAGCACGTGAAAT

GGCTGATAGCGGAGGTGTGGAAGAAGATGAATGCGGAGAGGGTGTCGAAGGATTCTCCATTCGGCAAAGATTTTATAGGATGTGCAGCTGATTTAGGAAG

GATGGCGCAGTTGATGTACCATAATGGAGATGGGCACGGCACACAACATCCTATAATACATCAACAAATGACCAGAACCTTATTCGAGCCCTTTGCATGA

>MlongTPS30

ATGTCTACCATTATTAGCGTACATCATCATCATCATCATCATGTGCCGAAGCCTCAACTAAATATTGTCCACACCAAAAACAAGAGAGCTTCTATCAATC

TCTCATGGAGTCTCTCTCCTTCTTCATCCGCCGCCTCTCGCCTCATGCCTCAGTCTATCTCCTCAAAACTAGATAACGAGAAGCCGCCCAATGAAACCAT

CCGACGTCGTTCTGGAAACTACGGGCCTTCACTCTGGGATTTCGATTACATTCAATCTCTCAACACTTATCACTACAAGGAGAAGCAATTGAATAGGGAA

GAAGAGCTGATCGTTCAAGTGAAGAAGATGCTGATGGGGAAAAAGATGGAGGCAGTGAAGCAATTGGAGTTGATTGATGACTTGAAAAATCTGGGGTTAT

CTTATTTTTTTCAAGGCGAGATTAAGAATATCTTAAATTCTATATATAACGAGCATAATTTTTCCCAAAATAATAAAGTTGGGGATTTGTATTTCACTGC

TCTTGGATTTAGACTCCTCAGACACCATGGTTTTGATGTTTCACAAGAAATATTTGACAGCTTCAAGGACGACAAGGGTAGTAGTGATGACACGAAAGGA

ATGTTACAACTTTACGAAGCATCTTTCCTTTTGAGGCAAGGTGAAGATACACTTGAGCTAGCTAAACAAATTTCCACCAAATTTCTTGAGGAAAAACTCG

AAGCCGGAATCTATGATGATAATCTATCATCATCGATTCGCCATTCTTTGGAGATCCCTCTTCACTGGAGAATCCAAAGCCTAGAGGCAAGATGGTTCTT

AGATGCTTATTCTATGAGGAAAGATATGAATCCAATCATTTTTGAGCTCGCCAAACTCGACTTCCATATTGTTCAAGCAACCCTCCAAGAAGAACTCAAA

GAGGTCTCAAGGTGGTGGAATAATTCTAGGCTCCCTCAAAAACTCCCATTTGTGAGGGATAGGTTGGTGGAAAGCTACTATTGGGCCCTTGGAATGTTTG

ATGCTCACAAATTTGGATATGAAAGAAAAACTGCTGCAAAGATTATAACCTTAATTACAGCTCTTGATGATGTTTATGATATCTATGGTACACTCGACGA

GCTCCAACAATTTACCCATGTCATTCGAAGATGGGATACTGAATCAGCCACCCAACTTCCTTATTACTTGCAATTATTCTATTTCGTACTATACAACTTT

GTTTCCGAGGTGGCTTATGATATTCTTAAAGAAGAGGGTTTCATCAGCATCCCGTTTCTACGCAGAGCGTGGGTGGATTTGGTTGAAGGATATTTACAAG

AGGCAAAGTGGTACCACAAAAAATATACACCAAACATGGAAGAATATTTAGCCAACGCCAGCATTACTATAGGGGCACCTGCAGTAATATCCCAAGTTTA

TTTTATGCTACCCAAATCCAAAGAGAAACCGGTGATCGAGAGTTATGATGAAATAATTCGCCTTTCTGGAATGCTCGTGAGGCTTCCTGATGATCTGGGA

ACGTCACCGTTTGAGATGAAGAGAGGCGACGTTGCAAAATCAATCCAGATTTACATGAAGGAACAGAATGTAACGCGGGAAGAAGCAGAAAAACACGTGA

GGTTTCTGATTTGGGAGGCGTGGAAGAAGATGAACACAGTGATGGCTCCGTTAAGGGATGATTTGGTTATGGCCGCAGCTAATCTTGGGAGAGATACACA

GTTTATGTATCTCGATGGAGACGGTAACCACACTCAGTTACACCACCACATTGCCAACTTGTTGTTCAACCAATACTAG

>MlongTPS31

ATGAGAGCTTCAAAGTCATGGCGATGGCGCCTCTCTTCTGCTTCTCCTGCTTCTCGCCTCCGGGCTTCGTACTCCACGCAACTAGATCTTAAGCCCGCAG

ATGAAGTCCGACGCTCCGGAAATTACCAGCCCACCCTTTGGGATTTCACTTACCTTCAATCTCTCACCAGTCAGTACAAGGAAAAGAGGCACTTGAGTCG

GGAAAGAGAGCTGATTGTGCAAGTGAAGATGTTGCTGCGGGGGGAAATGGAGGCAGTTGAGCAGTTGGAGTTGATTGATGACTTGCAAAATCTGGGATTG

TCTTATTTCTTTCAGGATGAAATTAAGCAAATGTTGAGCTGCATATATATCGACCACAAATATTTCAAAAGTGAGGAAATGGATCTGTATTTTACAGCTC

TTGGCTTCAGACTCCTCAGACAACATGGTTTTGATGTTTCCCCAGAAGTATTTGATTGTTACAAGAACAAGACGGGTAGGGATTTGAAAGCAAGCCTTTG

TGAGGATACAAAAGGGGTGCTTCAATTTTATGAAGCATCTTTCCTAGTGAGAGAAGGTGAAGATACATTGGAGGTAGCAAGAGAATTTGCCACCAAATTT

CTGCAAAAAAGGCTCGAGAGTGGTGATGCAAATGCAATTGACGGCCATATCTTCTCATCGATTCACCATTCTCTGGAGATCCCGCTTCATTGGAGGATAC

AAAGGCTAGAGGCAAGATGGTTCTTAGATGCTTACGCAAGGACACCCCACATGAATACAATTATTTTTGAGCTTGCCGCACTCGATTTCAACATTCTTCA

AGCAACACAACAAGAAGAACTCAAAGATATCTCAAGGTGGTGGAATAATTCAACGATTCCAGAAGATGTCCCATTTGTGAGGGACAGGCTAGTGGAAAGC

TACTTTTGGGCTCTTGGGCTCTTTGAGGGTCATGAATTTGGATATCAGAGGAAAACGGCCGCTAAGATTATTACTCTTATTACGGCCATCGATGATCTTT

ACGATATTTATGGTACATTAGATGAACTCGAATTGTTCACACGCGCATTTCAAAGGTGGGATAGTCCAGAATCAATCACCCATCTTCCTCATTACTTGAA

AATATTTTATTCTGTAGTCTATAACTTTGTCTCCGAGCTTGCATCCGATATTCACAAGGAGCAAGGATTCGCCAGTATTCCATATTTACAGAAATCGTGG

GTAGATTTGGTGCAAGGATATTTGCAAGAGGCAAAGTGGTACTATAGTGGTTATACACCAAGCACGGAAGAATATCTTAACAACGCCAGCATTACAATAG

GGGCTCCTGCAGTACTATCCCAACTTTATTTTACAATATCAAACTCGATAGAGAAACCGGTGATCGAGAGCTTGTTCAGATACGACAACATCATCCGCCT

TTCAGGATTGCTTGTTAGACTTCCCGATGATCTAGGAACTTCTCCGTATGAGATAAAGAGGGGGGACGTGCCGAAATCAATCCAGTGTTACATGAAGGAA

CGAAGCGTGAGCCAGAAAGAGGCAGAAGAACACGTGAGATTTGAGATAAGAGAGGCATGGAAGCAGATGAACACGGTGATGGTGGACGGCAGCTGCCCCT

TTACAAATACAAATGAAGTGGTTGTTGCTGCAGCTAACCTTGGAAGAGCGGCACAACTTATGTATCTTGAAGGTGATGGTAACCACTCTGAGTTACATCA

ACGGATTACGAGCTTGTTGTTCGACCCATATGTTTGA

>MlongTPS32

ATGGGGGCAAGGAGGTCGGCGAACTTCAAACCCAGTGTTTGGGATGACAAATACATTCAGTCCCTCACAACATCATATTCGGGGGATAAGTGCGTAGCCG

AAGCTGAGAAGCTGAAAACTCAGGTGAAATTTACGATAGACGAAGCCGAAGACGAACTAGCCCAGCTTGAGCTCATCGACAACCTTCAAAGGCTGGATTT

ATGCAGCCATTTCAAAGATCGCATTGCTAAAATCTTACTCAAAATCTATGCAGCTGCAAAAATTTGTGGAGATGACAATGAATGCATGGAAAAGGACCTG

CACTTGACCTCTCTCAAATTCAGACTTCTCAGGCAGCATGGATTCCACGTTCCTCAAGAGGTTTTCTGTTGTTTCATGGACGACAAAGGGAATTTCAAAG

ACTGCGCATCGGAAAACGTGAGGGGGCTCATGTCTCTGTATGAAGCTTCGTTTTTATCCATGGAAGGGGAAAGCGTGCTGGATTTGGCCAAAGATTTCTC

TTCGAATCATCTTACTCGAAAACTCGAAGAAATCGAAGATGGTGGTTTAGCTGAGGAAGTGAAGCATGCTTTGGAGCTTCCACTGCACTGGAGAATGCAG

AAACTTGAAGCTTTATGGTTCATAAAAGTTTACCAGAATAGATCCGACGCAAACACTCACCTCCTGCAGCTAGCTAAGTTGGATTTCAACATGGTGCAAG

CCACTTACCAGCACGAGCTCAAGCGCATGTCAAGTTGGTATAGAGAAACTGGGCTGCCGGAAAAGCTGAACTTTGCAAGGCACCGATTAGCAGAGTGTTT

CATGTGGGCCATGGGATTCATCCCTGAGGCCCACCTTGGGAAGTCAAGGGAGATTTTGACCAAGACTGCGGTGCTCATCACTATCATTGATGACATATAC

GATGTTTATGGAACGTTGGATGAACTCCAGCTCTTCACCGACACTATCGAGAGGTGGGGTATTAATTCGCTGGACCGACTTCCAGAGTATATGAGGATTT

GTTTCCTAGCTCTCTTCAACTCCGCTAATGAGCTGGCCTATCATATTCTAAGGGACCAAGGCTTCAACGTTATCTCAAATCTCAGAAGATTGTGGGGGGA

ACTGAGCAGAGCTTACTACTTAGAAGCTCTATGGTTTCACAGCGGATATTTCCCAACCACGAATGAGTATTTAAACACAGCATGGGTTTCCATTTCGGGT

CCTTTGCTTCTTTTCTATGCTTACTTCTCCACCACCGACTCCATTAACAAGGATGAGTTGCAGAGATTGGAGCAGCATTCTGGCCTCATTCGTTGGCCCT

CCATGGTTCTTCGACTCGCTGATGACTTGGGAACTTCATCCGAGGAAATAAAAAGGGGAGATGTTCCGAAGTCGATTCAGTGCATCATGAACGACACGGG

ATGCTGTGAGGAGGAAGCTCGAAAGCAAGTGAAGGAGTTGATAGAGGCGGCGTTGAAGGGAATGAACAAAGAGATATTGATGGAGAAGCCGTTGAAGGAT

TTCGGGGAAACTGCGATGAATCTGGGAAGAATCTCTCTCTGCATGTACCAACATGGAGATGGATTCGGTCTTCCTCATGCTGAGACCAAGAAGAATTTGG

TGTCTCTCCTCGTTCGCCCTATTCCGATGCTTTCATCTTCACTTACGATTTGA

>MlongTPS33

ATGGATTTGTGGTCCGATAATTCGAAACGTTTCCAATGTTCTCCTGCACCAGTAATATTTTCAGATATTTATTTTCAGCTAATGGAGATACAGTCTGCAA

CTCCGAGGAGGACAGCCAATTACAAGCCCAACATATGGAACTATGATTACTTACAATCTCTTACTACCAAATACCATGAAGAGTTGGAGGAGCACGAACG

AGAAGCCGAGGCGCTGAGAGATGAAGTTTGCAGCGAGTTTTGTGCGTTGAGAGATCCATTGATGAAGCTGGAGTTGGTAGATCGGATTAACAAACTTGCT

CTTTCTCATTACTTTGAGGAAGAAATCGACCATTACTTGAAGGAGATAGCATGCAAGGATTTGAGCCTCGACACTCATGATCTTTACTCTTCTGCTTTCT

ATTTCAGGATTCTAAGGGAGCATGGCTATCACGTTCCTCAAGATGGAATTATGCAGCTATTACATGATGAGTGGGACGAGAAAGCGAGGGTTGAAATATT

TGAAGCATCCCATCTTGGGGTGGACGATGAATGCATTTTTGATAGAGCAAAAGCGTTTGCGACAAAAAATATGATCTCCGATTGGAAAAACAATAATTCC

GGCAAGGGGCCGTCGCATCGGAATGTGAGCTGGTTTAATGTAAGAAGGTACGTGCATGAAAGTGACGACGAGTCAGCTGCACTCACTCGGTTGGCTCGAC

TCAGCTTCAACACGGCTCAAGTTCAGCACCAGAAGGAACTCAAGAACATTTTAAGATGGTGGAGAAATATGGGGTTGTTAGAAGTCCTAACCTTCTCGAG

AGATAGAGTGGTTGAAAGCTTTTTGTGGGCAGTGGGCGTTGCTTATGAGCCTCAGCATGGAAGCCTTAGAAAATGGCTCACAAAAGCCATCTTGTTAATA

CTAATAATTGATGATGTGTGGGACCCAAGTGAAATTCAGCAACTTCCTGAAGCCATAAAGAAGTGCTTCATGTTGCTGTATGATACTGCAAATGACATCG

ATCTTGAAATTCCAAAGGAAAAGGGTTGGGACTCTGTTCTGCCCCATTTCAAGAAAGTGTGGATAGGGTTCTGCAAATCATTGCTTGTGGAAGCAAAGTG

GGATAGGATGGGAGAATCTCCTTCCCTTAGAGAATATCTTGATAATGGATGGACTTCATCGTCAGGCCCAGTTCTCTCTCTTCATGTACTCATCGGCGTC

GGTTTAAAAAAGGCTGAAACTATGGCCTTCTTCCATTCCAACCAAGAAATCATCCACCAAGCTTCCCTCATTATCCGACTCTGCAACGACCAAGGAACTT

TTAAAGCGGAAGTGGAGAGAGGCGACGAGCCTTCATCAATCACGTGCCACGTGAGAGAAGCGAATGTGAGGGAGGAGGAAGGTCGTGAGCACATAAAAAA

CCTTATATTTGATTCATGGAGGAAAATAAATGCGATATTCATCGAGTGTCCGAAGGAGCATAAGGGAATGATGAGATATGTAGTGAATACAGCAAGAGTT

GCTAATTTTATTTATCAAAATGGAGACGGATTTGGGGTTCAAGATCGAGAGACCAGAGACCAAGTCCTATCGTGTTTGATCGAGCCTCTCCATGCATAA

>MlongTPS34

ATGGAGATGAAGTCTGCAACTCCGAGGAGGACAGCAAATTACAAGCCCAACATATGGAACTATGACTACTTACAATCTCTTACTACCAAATACCAGGATA

AGTTGGAGGAGCACAAACGAGAAGCCGAGGCACTAAAAGTTCAAGTTCGCAGCGAGTTTTGTGCGTTGAGAGATCCATTAGTGAAGCTGGAGTTGGTAGA

TCGGATTAACAAACTTGCTCTTTCTCATTACTTTGAGGAAGAAATCGACCATTACTTGAAGGAGATAGCATGCAAGGATGATTACACTAGTTTTAGCCTC

GACGCTCATGATCTTTACTCTTCTTCTTTCTATTTCAGGATTCTAAGGGAGCATGGTTATCACGTTCCTCAAGATGGAATTCTGCAGCTATTGGATGAAG

TGTGGGATGAGAAAGCGAGGGCTGAAATATTTGAAGCATCCCATCTCGGAGTGGAGGATGAATGCCTGTTTGATAGAGCAAAAGCGTCTGCGACGAAAAA

TATGATCTCCGATTGGAAAAACTATAATTCCGGCAAGGGGCCGTCGCATCGGAATGTGAGCTGGTTTAATGTAAGAAGGTACGTGCATGAAAGTAACGAC

GAGTCAGCTGCACTCACTCGGTTGGCCGGACTCAGCTTCAACACGATTCAAGTTCAGCACCACAAAGATCTCAAGGACATTTTAAGGTGGGACCCAAGTG

AAATTCAGCAACTTCCTGAACCCATAAAGAGGTGCTTCTGGTTGCTGTATGATACTGCAAATGACATCGATCTTGAAATTCAAAAGGAAAAGGGCTGGGA

CTCGGTTCTGCCCCATTTCAAGAAAGTGTGGATAGGGTTCTGCAAATCATTGCTTGTGGAAGCAAAGTGGGATAAGATGGGAGAATCTCCTTCCCTTAGA

GAATATCTTGATAATGGATGGACTTCATCGTCAGGCCCAGTTCTCTCTCTTCATGTACTCATCGGCGTCGGTTTAAAAAAGGCTGAAACTATGGCCTTCT

TCCATTCCAACCAAGAAATCATCCACCAAGCTTCCCTCATTATCCGACTCTGCAACGACCAAGGAACTTTTAAAGCGGAAGTGGAGAGAGGCGACGCGCC

TTCATCGATCACGTGCCACATGAGAGAAGCGAATGTGAGGGAGGAGGAAGGTCGTGAGCACATAAAAAACCTTATATTTGATTCATGGAGGAAAATAAAT

GGGATATTCATCGAGTGTCCGAAGGCGCATAAGGGAATGATGAGATATGTAGTGAATACAGCAAGAGTTGCTAATTTTATTTATCAAAATGGAGACGGAT

TTGGGGTTCAAGATCGAGAGACCAGAACCCAAATCCTATCGTGCTTGATCGAACCTTTGCATGTACACCCTTAA

>MlongTPS35

ATGCAGTCTGCAACTGCAGGTCCTCCTCCGAGGAGGACAGCAAATTACAAGCCCAACATATGGAACTATGACTACTTACAATCTCTTACTACCAAATACC

AGGATAAGTTGGAGGAGCACAAACGAGAAGCCGAGGCACTAAAAGTTCAAGTTCGCAGCGAGTTTTGTGCGTTGAGAGATCCATTAGTGAAGCTGGAGTT

GGTAGATCGGATTAACAAACTTGCTCTTTCTCATTACTTTGAGGAAGAAATCGGCCATCACTTGAAGGAGATATCATACAAGGATCGTCACAAACTTGCT

CATGACCTTTACTCTTCTGCTTTCTATTTCAGGATTCTAAGGGAGCATGGCTATCACGTTCCTCAAGAAGGAATTCTGCAGCTATTAGATGAAGAGTGGG

ACGAGAAGGCGAGGGTTGAAATATTTGAAGCATCTTATCTTGGAGTGGACGATGAATGCCTGTTTGAAAGAGCCAAAGCAGCGTTTGCGACGAAGAATAT

GATCTTCAATTGGAAAAACAATAATTCCGGCAAGGGGCCGTCGCATCGGAATGTGAGCTGGTTTAATGTAAGAAGGTACGTGCATGAAAGTGACGACGAG

TCAGCTGCACTCGCTCGGTTGGCCCGACTCAGCTTCACCACGATTCAAGCTCAGCACCAGAAGGATCTCAAGGACATTTTAAGATGGTGGAGAAATATGG

GGTTGTTAGAAGTCCTAACCTTCTCGAGAGATAGAGTGGTTGAAAGCTTTTTGTGGGCAGTGGGCGTTGCTTATGAGCCTCAGCATGGAAGCCTTAGAAA

ATGGCTCACAAAAGCCATCTTGTTTATACTAATAATTGATGGTGTGTATGATATTTATGGCTCCATGCATGAATTACACCAATTCACTACTGCAGTAGAA

AGGTGGGACCCAAGTGAAATCCAGCAACTTCCTGAAGCCATAAAGAGGTGCTTCTGGTTGCTGTATGATACTGCAAATGACATCGATCTTGAAATTCAAA

AGGAAAAGGGTTGGGACTCTGTTCTACCCCATTTCAAGAAAGTGTGGATAGGGTTCTGCAAATCATTGCTTGTGGAAGCAAAGTGGGATAAGATGGGCGA

ATCTCCTTCCCTAAGAGAATATTTAAATAATGGATGGACTTCATCGTCTGGCCCAATTCTCTCTCTTCATGTACTCATCGGTGTTGGTTTAAAAAAGTCT

GAAACTATGGCCTTCTTCCATTCCAACCAAGAAATCATCCACTGTGTCTCCCTCATTATCCGACTCTGCAACGACCAGGGAACTTTTAAAGCGGAAGTGG

AGAGAGGCGACGCGTCTTCATCAATCGCGTGCCACATGAGAGAAGCGAATGTGAGAGAGGAAGAAGCTCGTGATCACATAAAAAACCTTATATTTGATTC

ATGGAGGAAAATAAATGGGATATTCATCAAGTGTCCAAAGGCGCATAAGGGAATGATGAGATATGTAGTGAACTCAGCAAGAGTAGCTAATTTTTTTTAT

CAGAATGGAGACGGATTTGGGGTTCAAGATCGAGAGACGAGAGCCCAAGTCCTATCGTGCTTGATCGAGCCTTTGCATGTACACCCTTAA

>MlongTPS36

ATGTCTCTTGCTTCCACTCCGATCTCCTTCCGCCCATCCTCCACCGCGCACCCAGACCTCCCGGCTGCCGCCTTCTGTGCTCCGGCCGCGGCCCGCTCCT

CCGCCGCTTGTCTTCACCTTTTCCGACCACCTTCTCCCACCCCTACTTCTTCCCTGCAATGCAATGCAATCTCCAGACATCGTACACAAGAGTACATAGA

AGTGATTAAGAATGGATTACCGGTGATAAAGTGGCACGAGATCGTGGAAGATGACGAAGAAAAAGATTCTCTCAAGAAGGATAAGATAAGGGAGCTGGTC

GGAGTGGTTAGATCGATGCTGCAGTCCATGGACGACGGCGAGATAAGCATCTCCCCCTACGACACCGCGTGGGTGGCGCTCGTGTCCGACGGCGCCGCCT

CCGCGGCGCCGCAGTTCCCGTCGAGCCTCCATTGGATCTCGAGCAACCAGCTCCCCGACGGGTCGTGGGGGGACGACCGGACCTTCTCGATCTTCGACCG

GATCATCAACACCCTGGCCTGCGTCGTAGCGCTCACGTCTTGGAACCGGGATCCCCTCAAGACCCAAAGAGGGATTTGGTTTATAAAGGAGAACATGAAT

AGGCTGGAGGAAGAGAATATAGAGCACATGCCGATTGGATATGAAGTGGCGTTGCCTTCACTCATTGATATAGCTAAAAAGCTAAAAATCGATATCCCTC

ATGATACAAGAGGGTTGAGAGAGATCTATGCAAGAAGAGAGATTAAGCTCAAAAAGATACCAAGGGAGATACTGCATCAATTGCCAACAACATTGCTTCA

CAGCTTAGAAGGAATGGAAGGGCTAAAGTGGGAAAGGCTGTTGAAGTTACAAAGTGAAGACGGATCCTTCCTCTTCTCTCCTTCCTCCACTGCTTTTGCT

CTTCAACAAACTAAGGATGGTAACTGCCTCAAGTATTTGACCAACCACATTCACAAGTTTAAGGGTGGAGTACCAAATGTGTATCCTGTGGACTTGTTCG

AGCATCTTTGGGCAGTAGACAGGCTGCAAAGGCTTGGACTCTCGCGTTATTTTGAGCCAGAAATCGAAGAATGTGTCGCTTACGTTCGCAGGTACTGGAC

AGAGAAAGGAATATGCTGGGCAAGAAATTCACAAGTGCAGGACATAGACGACACGGCCATGGGGTTCAGGCTGCTGAGGTTGCATGGATACGAGGTTTCT

GCTGATGTGTTCAAGCATTTCGAGAGCGGGGGAGAGTTCTTCTGCTTCAAGGGGCAGTCGACGCAGGCGGTGACCGGAATGTACAATCTGTACCGAGCGT

CTCAGATAAAGTTTCCGGGAGAAGAGATTCTTCAAGAGGCAGCCGACTTCTCTGCTAAATTCTTGCATCAAAAGAGAGCTGATAATGAGCTCTTGGATAA

GTGGATCATCACCAAAGATCTTCCCGGCGAGGTGGGATATGCACTTGATGTTCCTTGGTACGCCAGCCTACCTCGAGTGGAAACAAGGTTTTATTTAGAA

CAGTACGGCGGTGAAGATGATGTTTGGATTGGCAAAACCTTGTACAGGATGCCATTTGTGAACAACAACAAATATTTGGAGCTGGCGAAACTCGACTATA

ATGATTGCCAAGCGTTACACCAACAGGAGTGGAAAAACATCCAAAAATGGTATCGTGGCTGCAGCTTGGGAGAATTCGGTTTGAGTGAAGAAAGCCTTCT

ACAAACATACTTCATCGCTTCTGCATCATTATTTGAGCCCGAAAAATCGCTAGAGCGCCTCTCTTGGGCCAAAACAGCGATTTTAATGCAAACCATTACG

TCGCACTTCGAAAATCGGCAACTCCCCGCAGAGCAAAAGCGCGCTTTTATCGAGAAATTCAAACACGGCAGCACCCTCAAGTACGCCAACGGAGGGAGGT

ATAAAACGAGGACGACTTTGGTGGGGACGTTACTGAGAACCCTAAATCAGCTGTCACTGGATATTTTGTTGGCTCACGGCCGAGATATTCATCAACCATT

GAACAATGCATGGCACAAGTGGCTGAACACTAGGGAACTGCAAGGTGGCGCGGAGCTACTGGTCCGGACGATCAACCTTAGCGGAGGGGTGGCGTCGGAG

GATCGGCTCTCGTCGTCCGATCCGAAGTACGAGCAGCTGATGGCGGCCACCGTGAGCGTCTGCGACAAGCTTCGTCTCTTTAAATCCCGAAAGGTGCAGG

ATGGGAATGGATGCATGAGCAATAGAGGAGGGATTACGACATCGGATATTGAATCGGAGATGCAAGAATTGGTGAAATTGGTTGTGACAAAATCTTCTTT

GGGAGATTTAGATTCTAAAATTAAGCAAAACTTTCTTACTATTGCTAAGAGTTTTTACTACGCTGCCTATTGTAATCAAGGAACCATCAATTTCCATATT

GCAAAAGTACTCTTTGAAAGAGTGCTTTGA

>MlongTPS37

ATGGCCTCTCTATCAATTGCATTCATCAACCGTTCTCCGGTGGCCAACAGCAAAGTTCTGCCGTCACCGGCGAAGCTTAACCTGCCGGAATATTTCGCTG

CCGGCAACTTCTTTTCGGCAGGAGCATGGCTGATCAGCAGTAAGAATCGCTCTCTCAACTGCCAGCTCAATCACAAGAGAACATCGAAAGTAATTCGAGT

TGCAACTGTGGATGCACCGCAAGTGCACGAGCACGAGCACGACGACTCCCGGCCTGTTCATCAAGGCCATGACGCGGTGAACGACATAGAAGATCCTATC

GAGTACATCAGAACACTACTGAGGACGACGGGCGACGGGAGAATCAGCGTGTCGCCCTACGACACCGCGTGGGTTGCCCTGATCAGGGACCTGCAAGGGC

GAGACGCCCCTCAGTTCCCATCCAGCCTCGAGTGGATCGTGAAGAATCAACTCGAGGACGGATCCTGGGGTGACGAGAAGCTTTTCTGCATATACGATCG

CCTCGTAAACACCATAGCATGTGTGGTGGCTCTTAGATCATGGAATGTCCATGCCGAAAAGGTCGAGAAAGGAGTGACGTATATAAAGGAAAATGTGGAT

AAATTCCGAGAAGGGAATGTGGAGCACATGACATGTGGGTTTGAAATCGTGTTTCCGGCGCTTTTGCAAAAGGCCAAAAGCTTGGGCATTGAACAACTTC

CTTATGATGCACCTGTGATAAAGGAGATCTACCACACTAGGGAACAAAAGTTGAAAAGGATTCCATTGGAGATAATGCACAAAGTGCCGACTTCTCTACT

GTTTAGTTTAGAAGGGTTGGAAAATTTGGAGTGGGACAAACTGTTGAAACTCCAGTCAACCGATGGTTCTTTTCTCACTTCTCCTTCTTCAACGGCCTTC

GCTTTCATGCAGACTAATGATGAAAAATGCTACCAATTTATCAAAAACACAGTAGACACTTTTAATGGAGGAGCACCCCATACTTATCCAGTTGATGTAT

TTGGGAGATTGTGGGCTATCGACAGGCTCCAGCGCCTCGGAATTTCTCGCTTTTTTGAATCTGAAATTGCCGATTGCTTAAGCCACATCCACAAATTTTG

GACGGATAAGGGAGTTTTCAGTGGAAGAGAGTCCGAGTTTTGCGACATCGACGATACTTCCATGGGAGTTAGGCTTCTCAGAATGCATGGATATGAAGTC

GATCCAAATGTACTTAGGAACTTCAAGCAGGACGACAAATTCTCTTGCTACGGTGGTCAGATGATCGAGTCTCCTTCTCCGATATACAATCTTTACCGAG

CTTCTCAGCTCCGATTTCCCGGAGAAGAAATTCTTGAAGAAGCCAATAAATTTGCATATGAGTTCTTACAAGAAAAGTTGGCACAAAACAAAATTCTTGA

TAAATGGGTTATATCCAAGCACTTGCATGATGAGATAAAGCTAGGACTAGAGATGCCATGGTACGCAACCCTACCTCGTGTCGAGGCTAAGTACTATACA

CAGTACTATGCAGGCTCAGGCGATGTGTGGATTGGCAAGACATTGTACAGGATGCCGGAAATCAGTAATGACACGTACCAAGAGCTAGCGAAGGCGGACT

TCAAGAGATGCCAAGCCCAGCACCAGTTTGAGTGGATCTACATGCAAGAATGGTACGAGAGTTGCAACATTGAAGAATTTGGAATTAGTAGAAAGGAGCT

TCTCCTTGCTTACTTCTTGGCGAGTGCAAGCATATTTGAAGTGGAGAGAGCAACCGAGAGAATTGCATGGGCAAAATCCCAAATCATTTCTAAGATGATC

ACTTCTTTCTTCAATAAGGAAACCACATCCTTGGAGGAAAAGAACTCTCTCTTCGATGAACTCAGAAATATTAATGGCCTAAACAAAAGTGCGAAGAAGG

GAGAAGATGGAGGTGCGAACATGGTGTTGGAAACCCTCAAAGAATACCTCGAGGGATTCGATAGATACACAAGACACCAACTGAGAAATTGCTGGAGCAC

ATGGCTAACGAAGCTGCAGCACGGCGGTGCCGACGGCGGCGCCGACGCGGAGCTCTTGACAGGCACATTAAACATTTGTGCCGGTCACATCGCCTTTAGG

GAAGAAATTCTCTCACACAATGAGTACAGCACTCTCTCCACCCTAACCACCAAAATCTGCCAACAACTTTCTAAAATTCAAAACGAAAAGGAGGTGGAAA

TAGAGGGAGCAAAAGCCAGCATAAAAAGCAAGGAACTGGAGAGAGACATGCAGGAATTGGTGAAATTGGTTGTTGGAAAATCAGGAATTAATAGAAATAT

TAAGAAAACATTTTTGGCAGTAGTGAAAACTTATTATTATAGAGCATATTATGCAGCGGAGACCATAGACACCCACATGTTCAAAGTGCTTTTCGAACCA

GTCGCTTGA

>MlongTPS38

ATGGCCGCTACATCCTCTCTTCGTGTGAGCAATGAACCAGCTGCCGGCGGCATGTTAAAGCTACTAGCAAAGGCTCACCTCCCTCAATATCACACCGTAT

GTGCATGGCTGAACAGCGGTAGCAAACACGAGCCCTTGAGTTGCAGAATTAGTCGCAGGAAAATTTCAGAAGTAACTGAATGTCGAGCAACAAGCCTGGA

GTCGTCACAACTGACAGAAAAAGTCAGCTCTCCTCCTCAATCTCCCGAAGAGGAGAATAAAAAGATCGAGGACTCTGTCGAGTACATTAAAAATTTGTTG

ATGACATCTGGCGACGGGCGAATAAGCGTGTCACCTTATGACACGTCGATAGTAGCCCTAATAAAGGACTTGAATGGACAAGATGCCCCTCAGTTTCCGT

CGTGCCTAGAGTGGATAGCGGAGCACCAACTGGCCGATGGGTCGTGGGGGGATGAGTTCTTCTGTATTTACGATCGGATTGTAAATACATTAGCCTGCCT

CGTCGCCCTGAAATCATGGAACCTTCATCCTCACAAGATTGAAAAAGGAGTGTCGTACATCAAGGAAAATGTACAGAAACTCAAAAATGGGAAAGCGGAG

CACATGACGTCGGGGTTCGAAATCGTGGTTCCCGCCATTCTGGAGAGAGCCAAAGCCTTGGGCATCCATGGTCTTCCCTACGATGATCCCATCATTAAGG

AGATTGCTAACACAAAGGAACGAAGATTGAACAAAATTCCCAATGACTTGATATACGAATCTCCAACAACTCTACTATTCAGTTTAGAAGGGTTGGAAAA

CTTGGAGTGGGAAAGGATACTAAAACTGCAGTCAGCTGATGGCTCCTTCCTTACTTCGCCGTCGTCCACCGCCTTCGTCTTCATGCAGACTAAAGACAAG

AAATGCCTCAAATTCGTCGAGAACGCCGTCAAAAACTGCGACGGAGGAGCGCCGCATACGTATCCAGTGGATGTCTTCGCAAGGCTTTGGGCAGTTGACA

GACTACAACGCCTAGGGATTTCTCGTTTCTTTCAACATGAGATTAAATATTTCTTAGATCACATAAATAGTATTTGGAGCGAGAAGGGAGTTTTCAGTGG

ACGAGATTCACAATTATGTGATATTGATGACACGTCCATGGGCGTTAGGCTTCTCAAACTGCATGGATACAACGTTGACCCAAATGTGCTCAAACATTTC

AAGCAGCAAGATGGAAAATTTTCTTGCTATAGCGGTCAAATGATCGAGTCTGCATCTCCAATATACAATCTCTATAGAGCTGCTCAGCTTCGATTTCCAG

GAGAAGAAATTCTTGAAGAAGCAAGTAAATTTGCCTATAACTTTTTGCAAGGAAAGCTAGCTAAAGATGAAATTCAAGAAAAATGGGTCATATCTGAGCA

CTTGATTGATGAGATAAAGTTGGGACTGAAGATGCCATGGTACGCGACTCTACCCCGAGTCGAGGCTGCATATTATGTGGAGTATTATGCTGGTTCTCGC

GATGTATGGATTGGCAAGTGTTTCTACAGGATGCCAGAAATAAGTAATGATACATACAAAGAGGTTGCCATATTGGACTTCAACAGATGCCAAGCTCAAC

ATCAGCTTGAATGGATTTATATGCAAGAGTGGTATGAAAGTAGCAACATTAAAGATTTTTGGATAAGCAAAAAAGAACTACTTGTTGCTTACTTTTTGGC

TGCATCAACCATATTTGAACCTGAAAGAACACAAGAGAGGATTACGTGGGCAAAAACCCTAATCATTTCTAAGATGATCACATCATTTTTTAACAAACAA

ACTACACTATCATCGGAGCAAAAGACTGCCTTCTTAACACAACTTGGACATAGTTTCAATGGTCATTACAAAATAATAAATAGTGGTGAGAAAAATTCTG

GACTAGCTCAGACTCTGATGGCAACCTTCCAGCAACTACTTGACGGATTTGATAGATACACTCGCCATCAATTGCGCAATGCTTGGAACCAATGGTTCAT

GAAACTTAAGCAAGGAGCAAAGGCCAGCGGCGGGGCAGACGCGGAGCTCATAGCAAACACGCTCAACATCTGTGCTGGCCTCGCTTTCAACGAGCACGTA

CTATTACAAGTAAAATCTGCAAGCGGCTCAGCCAGATTGAAGATAAAAAGACGCTTGAAATTATCGATGGCGGCATAA

>MlongTPS39

ATGAGCTCTCTATCCTCTATGAATTTGAGCAATGCACCAGCTGCACGCTGCAGGTTAAAGCTTCACTCGCCGGAATTTCACGCAGTAGAAAAAAGTGTGG

ATGTCTTACAAGTTGGTGAAAAAGTCAGTCCTCTTGTTCAGACACCCAAAGAGGTAAATAAAAAGATCGAGGACTCCATCAAGTACATCAAGAATCTGTT

GATGACATCAGGCGACGGGCGCGTAAGCGTGTCGCCTTACGACACATCGATAGTAGCCCTAATAAAGGATGTGAAAGGACGAAATAGCCCTCAGTTTCCG

TCGTGCCTGGAGTGGATAGGGCAGCACCAAAAGGCCGATGGCTCGTGGGGAGATGACTTCTTCTGTATTTATGACCGGATTGTAAATACTTTAGCATGCC

TCGTAGCGTTAAAAACATGGAACCTTCACCCAGACAAGATTGAAAAAGGTGTGTCGTACGTCAAGGAAAATGTGCATAAACTTAAAGATGGGAGTGCCGA

GCACGTGACGTCGGGGTTCGAAATCGTGGTTCCGGCCACGCTTGAAAGAGCGAAAGCCTTGGGCATCCAAGGCCTTCCTTACGATGATCCCATCATTAAG

GAGATTACTAACTCAAAAGAACGAAGATTGAAGGATTTACCCAAGGATTTGATATACGAAACCCCAACGACTTTACTATTCAGTTTAGAAGGGTTGGAAA

ACTTGGAGTGGGAAAGGATACTAAAACTGCAGTCAGCTGATGGCTCCTTCCTTACTTCGCCGTCGTCTACCGCCTTCGTCTTCATGCAAACGAAAGACGA

GAAATGCCTCAAATTCGTCGAGAACGCCGTCAAAAACTGCGACGGAGGAGCGCCGCATTCATATCCTGTGGATGTCTTTGCAAGACTTTGGGCAATTGAC

AGACTACAACGCTTAGGGATTTCTCGGTTCTTCCAACACGAGATTAAATATTTCTTAGATCACATTCACAGTGTTTGGAGTGAGAATGGAGTTTTCAGTG

CACGACATTCACAAATTTCGGATATTGATGACACGTCCATGGGTATCAGCCTTTTAAAAATGCATGGATACCACGTTCATCCAAGTGCACTTAAACATTT

CAAGCAAAAGGATGGAAAATTTTCCTGCTTCAGTGGTGGTCAAATGATCGAGTCTGCATCTCCAATGTACAATCTCTACAGGGCAGCTCAACTCCGATTT

CCAGAAGAAGAAATTCTTGAAGAAGCAAGTAAATTTGCCTATAACTTTTTGCAAGGAAAGCTAGCTAAAGATGAAATTCAAGAAAAATGGGTCATATCCG

AACACTTAATTGATGAGATAAAATTGGGACTGAAGATGCCATGGTACGCGACTCTACCCCGAGTCGAGGCTGCATACTATATAGAGTATTATGCTGGCTC

TGGCGATGTATGGAATGGCAAGACTTTCAACAGGATGCCAGAAATCAGTAATGATACATACAAAGAGGTGGCCATAATGGAATTCAACAGATGCCAAGCA

CAACATCAGTTTGAATGGATTTATGTGCAAGAGTGGTATGAAAGAAGCAACGTTAAAGATTTTGGGATAAGCAAAAAAGAATTACTTGTTGCTTACTTTT

TGGCTGCATCAACCATATTTGAACCTGAAAGATCACAAGAGAGGATTACTTGGGCGAAAACCCTAATCCTTTCTAAGATGATCACATCATTTTTTAACAA

AAAAACTACGCTATCATCAGAGCAAAAGACTGCCTTCTTAGCACAAGTTGGGAATAGTTTCAATGATCCCGATAAACTAACTAGTGGTGAGAAAGATTGT

GGACAAGCTGAGAGTTTGATGGCAACCTTCCATCAATTGCTATGCGGATTCGATAGTTACACTCGCCATCAATTGCGAAATGCTTGGAGCCAATGGTTGA

TGAAGCTGCAGCAAGGAGAGGGCAACGATGGTGCAGATGCGGAGCTCATAGCTAACACGCTAAACATCTGTGCTGGTCTCATCGCCTTCAATGAAGACGT

GTTGTCGTGCAACGAATACACGACTCTCTCCTCCCTCACTAATAAAATATGTCAGCGGCTTAGCCAGATTCAAGATAAAAAGACGCTTGAGATTATCGAT

GGCGGTATAAGAGATAAGGAACTGGAGCAAGATATGCAAGCATTAGTGAAGCTAGTCCTTGAAGAAAATGACAGCGGCATAGATAGGAACATCAAGCAAA

CATTCTTATCAGTTTTCAAGACTTTTTACTACAGCGCCTACCATGATGCTGAGATGATTGATGTTCATATTTTCAAAGTACTCTTTGGACCAGTCGTCTC

ATAA

>MlongTPS40

ATGCATTTTGCTTCCAACACCTCCTCCCTCCTCAGATCAACCGCCGTCGGAGTCTGTGTTTTTACTTTATGCGCCATACTGCCATCTAGTTTCAACGTAA

AGTTTGGGAGCGTAAGTTATGGAGATGAGAATGAGATACGTAGAAATAGGCAAATAGCAGTGGAAGCGTTGACAAATTTCGTCGGTCAAACATGGGAGGA

TACCTCAGCAAAGCCCATGAATCAAGAAAAGGAGGGGATAAAGCAACTGAGGGAGAGGATTCGATGGATGCTGCAGAACATGGAGGACGGCGAGATAAGC

GTGTCGCCATACGACACCGCATGGGTGGCCTTGGTGGAAGATGTCGACACCCGCGGACGACGGCCGCAGTTTCCGACAACCCTCGACTGGATTTCGACGA

ATCAGCTTGCCGACGGATCGTGGGGGGATCGCAAATTCGTTCTCTACGACAGAATCCTCAACACTTTAGCGTGTGTGGTTGCACTCGCCACATGGAAAAT

GCATCCTCACAAATGCGAACAAGGGATGAGGTTTATAAGAGAGAATATTGAGAAACTTGGGAATGAAGAAGATGAGGGGCTGATGCCCGTAGGGTTCGAA

GTCGCACTGCCATCACTCATCGAAACAGCTACAAAACTAGGAATTCGAATCCCAAGTGATTCTCCAGCCATGAAAAATATTTATGCAAAGAGAGATTTAA

AACTCAGAAATATACCAATGGATTTAGTGCACAAGAAGCCCACATCACTTCTCTTCAGCTTGGAAGGGATGGAAGGCCTTGTTTGGGAGAAGCTCTTGAA

ATACCGGGTTGAGGGCTCGTTCCTTACGTCGCCTTCATCCACCGCCTACGCCCTCCACCACACCAAGGATGAGTTATGCCTCCAGTATCTGCTCAAGACT

GTCAACAAATTCAACGGCGGAGTTCCGAATGTGTACCCTGTCGACATGTTCGAGCATCTGTGGTGCATAGACCGCCTGCAAAGGTTAGGAATTTCTCGGT

TTTTTCAGGCTGAAATTGAAGAATGCATCAACTATGTTTACAGATACTGGACAAGAAAAGGAATTTGTTGGGCAAGAAATAGCAATATCCAGGATATTGA

TGACACCTCAATGGGATTCAGGCTTTTAAGGTTGCATGGTTACGATGTTTCTATAGATGCTTTCAAACAATTCGAGAAAGGAGGAGAATTCTGTTGTATT

CCAGGGCAGACGACCCCCGCTATAACAGGAATGTACAACCTGTATAGAGCTTCTCAAGTGATGTTCCCTCAAGAACCCATACTTAACGATGCCAAAAACT

ACTCAGCCAACTTCTTGCATCAAAAAAGACTCAATAATGAAATAGTAGACAAGTGGATCATCACCAAAGACCTTTCGGGCGAGGTGGGATATGCATTGGA

TGTGCCGTTCTACGCTAGTCTGCCTCGACTGGAAGCACGATTCTTCTTAGAACAATATGGGGGCGATGATGATGTTTGGATTGGAAAAACTCTATACAGG

ATGCCATATGTGAATTGTGACACTTACCTAGAGCTGGCAAAGTTAGACTACAAAAACTGTCAGTCTGTGCATCTATTTGAGTGGGCGAACATGAAAAGAT

GGTATAGGGATTGGAACCTAGGAGAGTTTGGGCTGAGCGAAAGAAGCCTTCTCCAAGCTTACTACATAGCAGCCTCGACTATATTTGAGCCAGAAAAATC

AGGAGAGCGGATGACATGGGCTAAAACAGCAATTTTAGTCGAGACAATCACATCCCTACAACTCTCTGATGAACAAAAGGGTGACTGTATTGATGAATTT

AGCATTCTCAGTTACCAAAATGGAGAAAGATGTAAACCAAGAAACAGATTGGTAGAGATTTTAATCAACACTGTAACACTAGAAGCAGAAGTCAGAGGCA

CAGATCATCAGTTGTCTACTGCGTGGCTAAAATGGTTGAAGAAATGGAAAGAAGGAGGTGACCTGGGGGAAGCAGAAGCTCGGCTTCTTCTTCACTCGAT

ACACTTGAGCTCTGGATTGAACGAATCATCATTTTCCCATCCCAAATATCACTTGCTCCTCGAGGCTACCTGCAAAGTTTGCCACCAACTTCGCCTATTC

CAGAATCGAAAGGTGCATGATGAACAAGGGTGTATAAATCGATTGGCGATTGGGATAACTTACCAAATAGAAGCAGGCATGCAAGAAGTGGTGAAATTAG

TGTTCACCAAAACTTCAAAAGACTTGCCTTCTCTTATCAAGCAAAGCTTTTTCAATATAGCTAGAAGTTTCTACTACACTGCTTATTGTGATGCAGATGC

TATTGACTCACACATAGACAAAGTATTGTTTGGAAAAATAGTCTAG

>MlongTPS41

ATGAAAGTTGAAGCCGTCACCCCATTGCCTGCACCGTTTAACTTTCTCGTCGCATCCTTCCACAATTCCAGCAACGTGAAAAGCCGTCTTCCTTTCCACC

GTCGCCGCCGCTGCTCCGCCCCTGGTCCGCCGCCCGGTACCAAAACAAGAATGTCACTCTCCTTCAACCCCAGAGTTACCCCTCTCTCCGGCCACAGAGT

TCAGAGCAGAAGAGAAATTTTCCCAGTCGATAGATTTCTGATGTTCACAAATAAGTCATCAGTCGCCGTCGAGTGCAACTTCACTAGGGCAATGCCCTTC

CCTCAGGATTTGATGGGGAAAATAGAAGATAAATTCAAGGGCAAAATCGGTAATTTCCCTACTGCCGAAGCTGGCGATGTGCCTTCCGATCTCTGCATAA

TCGACACCCTCCAAAGATTGGGAGTCGACCGTTACTTCGAATCCGAAATCGATTCCGTTCTCGAGGAGACCTACATGTTATGGCAAAAGAAGGATTTAGT

TATATATTCGAATGTAGCAACTCATGCAATGGCGTTTAGACTTTTGCGAGTGAAAGGATATGAAATTTCTTCAGAGGAATTGGCTCCGTATGCTGATCGA

GAGTGTGTTAATGTGGACACGGCTGACGTGGCGACGGTTATCGAGCTGTACAGAGCAGCACAGGAGAGGATTTATGAAGATGAGAGGAGCCTTGAGAAAC

TCCATGGTTGGACCACCAACTTCCTCAAGCACCAGCTGCAGAGTAACTCAATTCTTGATCAAAAACTGCATAAATTGGTGGAATATTACTTGAAGAACTA

CCATGGAATGTTAGATAGAATGTGTGTTAGGCAGAGCCTCGACCTGTACGACGTATGCCATTATCAAAATCTAAAAGTTGGAGATAGGTTCTTTAGGTTG

TGTAATGAAGATTTCTTAGCATTCACGAGGCGAGATTTCAATGTTTGCCAAGCCCAACACCAGAGAGAACTTGATCAGCTGCAAAGGTGGTATGCAGATT

GTAGGTTAGACACCTTGAAGTTTGGTAGGAATGTACAACGCGTTTCGAATTTTCTGACTTCAGCGATTATCTGTGATCCTGAACTATCCGATGTTCGTCT

AGCCTTTGCCAAACATATAGTGCTGGTAACGCTTATTGATGATTTCTTCGATCATGGTGGATCTAGGGAAGAGTCCTATAAGATCCTTGAGTTAGTTAAA

GAATGGAAAGAGAAGCCAGCTGCAGAATATGGTTCTGAGGAAGTCGAAATCCTCTTCACAGCTGTGTATAACACAGTAAACGAGTTGGCAGGGATGGCTC

GTGTCGAACAAGGACACTGCGTTAAAGAATTTTTCATTGAACTGTGGGTTCAGATACTATCGATTTTCAAGATAGAACTAGATACGTGGAGCGATGACTC

GCCACTAACCTTGGATGAGTACTTGTCTTCCTCGTGGGTGTCGATTGGTTGCAGAATCTGCATCCTCGTCTCGATGCAGTTCCTTGGTGTGAAATTATCC

GATGAAATGCTAATGAGCGAAGAGTGCACTGATTTGTGTAGGCATGTCTCCATGGTTGATCGCCTCCTCAACGACGTGCAAACTTTCGAGAAGGAACGGA

AGGAGAACACAGGAAACAGTGTGAGCCTTCTGCTAGCAGCAGCAAAGAAAGACGCTAAGCCGATTACCGAAGAGGAAGCCATTGCAAAGATAAAAGAAAT

GGCTGAATGTAACAGGAGAGAACTGATGCGGATTGTGTACAAAACCGGAACCATTTTCCCGAGAAAATGCAAAGATATGTTCTTGAAGGTGTGCAGGATC

GGGTGCTACCTGTACTCGAGCGGAGACGAATTTACGTCTCCGCAACAGATGAAGGAAGATATGAAGTCCTTGGTTTATGATCCCCTAGCAGTTCAGCCTC

TTGAAGCTAAAGAACTTACTTCTCAGCAAATAGTTTTGCCAGTTGATCATGCTTTCTTTTATCACAAGGGAAAAGAATATCTTGAGTTGAGCAACTACCT

TCAGCTCTGTGAAGATCTGCACAATTATTATTCCTCAAATACCTTCAATGAAGAATTCATCGGTTTTGGACGAGATTCATGGTAG

>MlongTPS42

ATGACAGTTGCCTTCAACATCAGATCAATTATACCTTTCTCTGGCCACAGTGTCTGGAGCAGGAGACACCAAATATATCCAGTTCACGGATTTCGGAGGA

TGATCACTACCAATAAGTCGTCCGTCACCGATGAATGCAACATTAAGTCAACAGATTTGATGGGGAAAATAGAAAAGAAGTTGAAGGGCAAAAAGGGTAA

TACCAGTCAAGCTGGGGATATAGCCACAAATCTGTGTATAATCGACACCCTCCAAAGATTGGGAGTGGAGCGTTACTACCAAATTGAGGTCGATTCTGTT

CTAGAGGACACATACAGGCTATGGCAGCGGAAAGATAAAGATATATATTCAGACATTAGTATTCATGCAATGGCGTTCAGACTTTTGAGAGTCAAAGGAT

ATCAAGTCTCTTCAGAGGAATTGGCTCCATACGCTGATGAAGAGCGAATGAATCTGCAAACAATTGACGTTGCGACGGTTGTCGAGTTGTACAGAGCATC

GGAGGAGAGAATTTATGAAGATGAGAGCAGCCTCGAGAAACTTCATGCTTGGACCACCACCTTCCTCATGCATCAGCTGCAGACTAACTCCATTCCTAAC

AAGAAATTGCACAAGCTTGTTAAATACTATTTGAACAACTACCATGGTATAATACAAAGAATTGGAGTTAGGAAGAACCTCGACCTCTTCGACATAAGCT

ATTATCAAACTCCAAAAGCTGCACATAGGTTCTCTAATCTATGTAATGAAGATTTTCTAGCATTCGCGAGGCGAGATTTTAATATATGCCAATCCCAACA

CCAGAAAGAACTTCACCACCTGCAGAGGTGGTATGCAGATTGTAGGTTGAATACCATGAAGTTTGGAAGAGATGTAGTTGGCATTGCTCATTTTATGGCT

TCAGCAATTGTTAATGGTGATCCTGAATTTTCCGAAGCTCGTCTAGCCCTAGCCAAACATGTTGTGCTGGTTACGCGTGCTGATGATTTCTTCGATCATG

GTGGGACTAGAGAAGAGTCATACAAGATCATTGAATTAGTAAATGAATGGAAAGAGAAGCCAGCAGCAGAATATGGTTCTGAGAAAGTTGAAATCCTCTT

CACAGCAGTATACAATACAGTGAATGAGATTGCAGAAATGGCTCACCTCGAACAAGGGCGCAGTGTCAAAGAATTTCTAATCAGAATGTGGGTTCAAATA

CTATCGGTCTTCAAGATGGAACTAGACACGTGGAGCGACAAGACGGAAATAAGCTTGGATGAGTACATGTCTTCGTCATGGGTGTCCATTGGTTGCAGAA

TATGCAGCCTCATGTCGATGCAGTTTCTCGGTGTAAAATTATCCGATGAAATGCTTATGAGTGAAGAGTGCAATGATTTGTGTAAGCATCTTTCACTGGT

TGATCGCCTCCTCAACGACGTGCAAACTTTTGAGAAGGAACGCAAGGAGAATACGGAAAGCAGCGTGACGATTCTGGTAGCGGCAGCTGAGAGAGATGGA

AGAGTTGTTAGTGAAGAGGAAGCTATAGGAAATATAAGAGAAATAGTTGAATATAACAGGAGGAAATTGATGCAGATTGTGTATAAAAGTGGAACCATTT

TCCCAAGAGAATGCAAGGAAATATTCTTGAATTCATGCAGGGCTGGCTGTTATGTGTATGGGAGGAGCGATGAATTTTCTTCTCCTCAACAAGTGATGAT

GGAAGATATGAAAGCATTGATTCATCAACCTGCATAA

>MlongTPS43

ATGTCACTCGCCTTCAACATCGGAGTTGTCCTTTTCTCCGGCCACATAGTCCGGAGCCCAGTTCACGGATTTCCGACGATGATCACTACCAACAAGTCGT

CTCTCACCAATGAATGCAACCTTCGGACGACAGATTTGATGGGCAAAATAGGAAAGAAATTGAAGGGCAAAAGGGTAGATATGACTCAAGCTGGGCTGAC

TATTGAAGCTGGGGATATGGCCACAAACCTGTGTATAATTGACACTCTCCAAAGATTGGGAGTGGAGCGTTACTACCAAAGTGAGATCGATTCTGTTCTA

GAAGACACCTTCAGGTTGTGGCTACGTAAAGAGAAAGAAATATATTCTGATATTACTATTCATGCAATGGCATTTCGGCTTTTGCGAGTCAAAGGATATC

AAGTCTCTTCAGAGGAACTGGCTCCATACGCTGATGAAGAGCGAATGAATCTGCAAACAATTGACGTTGCGACGGTTATCGAGCTGTACAGAGCATCGGA

GGAGAGAATTTATGAAGAAGAGAGGAGCCTTGAGAAACTCCATGGTTGGACCACCACCTTCCTCATGCACCAGTTGCAGACTAACTCCATTCATGATCGA

AAACTGCATAAACTGGTGGAATACTATTTGAAGAACTACCACGGCATTATACATAGAATTGGACATAGAAGAAACCTCGACCTATTCGACATAAGCTATT

ATCAAAGTCCAAATTCTGCAGATAGGTTCTCTAATCTCTGCAATGAAGATTTTCTAGCATTCGCGAGGCAAGATTTTAATATATGCCAAGCCAAACACCG

GAAAGAACTTGACCAACTGCAGAGGTGGTATGCAGATTGTAGGTTGGATACCATGAAGTTTGGAAGAGATGTAGTTCGCATTGCTCATTTTATGGCTTCA

TCAATTGTTGACGGCGATCCTGAATTCTCCGAAGCTCGTCTAGCCTTAGCCAAACATGTTGTGCTGGTTACGCGTGCTGATGATTTCTTCGATCATGGTG

GGACTAGAGAAGAGTCATACAAGATCATTGAATTAGTAAACGAATGGAAAGAGAAGCCAGCAGCAGAATATGGTTCTGAGAAAGTTGAAATCCTCTTCAC

AGCAGTATACAATACAGTGAATGAGATTGCAGAGATGGCTGGTGTGGAACAAGGGTGCAATGTTAAAGAATTTCTAATTGGAATGTGGGTTCAGATACTA

TCGGTCTTCAAGATGGAACTAGACACGTGGAGTGACAAGACGGAAATAAGCTTGGATGAGTACATGTCTTCGTCATGGGTGTCGATTGGGTGTAGAATCT

GCAGCCTCATGTCGATGCAGTTTCTCGGTGTACAACTATCCGATGAAATGCTTATGAGTGAAGAGTGCAATGATCTGTGTAAGCATCTTTCACTCGTTGA

TCGCCTCCTCAATGACGTACAAACTTTTGAGAAGGAACGAGGGGAGAATACGGGAAGCAGTGTGACAATTCTGGTAGCAGCAGCTAAGAGAGATGGAAGA

GTTGTTAGTGAAGAGGAAGCTATAAGAAATATAAGAGAAATAGTTGAATATAACAGGAGGAAATTGATGGAGATTGTGTATAAAAGAGGAACCATTTTCC

CAAGAGAATGCAAGGATATATTCTTGAATTCATGCAGGGCTGGTTATTATGTGTATGGGAGCAGCGATGAATTTTCTTCTCCTCAACAAATGATGATGGA

AGATATGAAAGCATTGATTCATCAACCTGCATGCATAACACTTCCACCTTCATAA

>MlongTPS44

ATGCCATGTAGGATAAGTTTTAGTACTACGAACTCCTCCTCTCGAGGACACCTCATTACTATATGTACAGAGAAAGATAAGGATAAATTTGATAAAAGGA

TCCTGCCTTTAACAATATTTGCAGCAATAAAAATGCTACCTGCACGTCTAAAATTCAAAAACAATCCTTTCATGGCCCATAGAATCCGGAACAGCAGCAA

AGAATTTCCAGCAAGCATTCCCGTCACTGCCAAGTGCAGCCTCGATAGTTCAGAAGATTTGATAGCAAAAATAAAAGAGAGAATGAATGGGAAAGTCAAG

GTTTCGCCGATGACAGAATATTCCGACAGCAAGCCTCAATTTCCGGCGTGGGGATTGCAATCCAAAACATGGGTAGATGTGTGCCCTAGTCTGTGTCCCG

TCGATCGCCTTCAAGATTTAGGAGTGGACCGCTTCTTTCAATCAGAAATTCAAAATGTTCTACATGAGGCATTTAGATTATGGCAGCAGAAAGACGAAGA

AATTTTTGGGGACGTTACGTGTTGTGCCATAGCATTTAGGCTCTTGCGACTCGAGGGTTATCATGTCTCATCAGATGAACTGGGTGAATATGTTGACGAA

GAAAATTTCCTTAATAAGGTAAGTTTAGCATCGAGTGGTACAGAAACAGTTCTTGAGCTGTACAAAGCATCACAGGTAAGATTACCTGAAGACGACGACG

ATGGCAATCTTGAAAAATTGCACGATTGGACCTTCAACTTTTTGAAGAAGCAATTAAGGTCTAAAACCATTCTTGATAAAAATTTAGAGAGAAAGGTGGA

ATTCAACTTGAAGAATTACCATGGCATATTAGATGCTATTAAACACAGACGAAGCATTGATCTATTATCTGATGTTGATCACACACCAATCTTCAAAACT

GCATATAGTTGCCCTGCACTGTATTATGAGGAATTATCTCTATTGTCGGCGCACGATTTCATGACACGGCAAGCTCAAAACCAGAAGGAGCTTTATATAT

TGCTGAGGTGGTATGATGAATGTAGACTGGACAAGATGGAGTACGGACGGAACGTGATACGCGTTTCCCATTTCCTCAACGCAAACAATTTCCCCGATCC

TCAGCTCTCTGAAGCTCGTCTATCCTTTGCGAAAACCATGACTCTCGTCACTCGTTTGGATGATTTCTTCGATCACCACGGCTCTAGAGAAGATTCTCTC

CTCATCGTTGAATTAATAAGACAGTGGAATGAGCCGTCAACAATAAGATTCCCCTCCGAAGAAGTCGAGATTCTCTACTCTGCGCTTCACTCCACAGTTA

CAGATATAGCAGAGAAGGCTTATCCCATGCAGGGCCGCTGCATCAAATCACTCATAATTCATCTGTGGGTTGAGATATTGACGAGTTTCATGAGCGAAAT

GGACTCATGCACTGCGGAAATTCCACCAAACATGCATGAGTACTTAGGGTTTGCATGGATCTCCATCGGCTGCAGAATTTGCATTCTTGTAGCCATCCAT

TTCTTGGGGGAGAAGGTATCCGAAGAACTGGTTATGGGTGCAGAGTGCACCGAGTTATGCAAACACGTTTCTTCAGTCGCACGCCTTCTCAACGATCTCC

AAACCTTTATGAAGGAGAGAGAAGAGAGGAAGGTAAACAGCGTGATAATTCAGCTCAACGGTGATGAGAAGATACCGGAGGAGGAGGCGGTATCGAATGT

AGAGAAAATGATTGAATTTCACAGGAAAGAGCTGCTGAAGATGGTGGTGCAGAGAGAAGGAAGCTTGGTTCCTAAAAAATGGTTTGTCTCCGCCCCTGGC

CAGACGGTATGCAAGTTGGGGCCTTAA

>MlongTPS45

ATGAATGGAAAAGTCAAGATTTTGCCGTCGGCACCCTTGCTGACGACGGAATATTCCGGCAGCAAGCCTCATTCTCCGGCGTGGGGTTTGCAATCCAAAA

CATGGGTGGATGTGTGCCCTAGTCTGTGTCTCGTCGATCGCCTTCAAGATTTAGGAGTGGACCGCTTCTTTCAATCAGAAATCCAAAATGTTCTACATGA

GGCATTTAGATTATGGCAGCAGAAAAATGAAGAAATTTTTGGTGACGCTACGTGTTGTGCCATGGCATTTAGGCACTTGCGACTCGAAGGCTATCATGTC

TCATCAGATGAACTGGGTGAGTATGTTGAAGAAGAAAATTTCCTTAATAAGGTAACTTTAGCATCGAGTGGTACAGAAACAGTTCTTGAGCTGTACAAAG

CATCACAGGTGGAATTCAACTTGAAGAATTACCATGGCATATTAGATGCTATTAAACATAGACGAAGCATTGATCTATTATTTGATGTTGATCACACACC

AATCTTCAAAACTGCATATAGTTGCTCTGCAGTGAATAATGAGGATCTATTTCTATTGTCGGCGCAAGATTTCATGGCTCGCCAAGCTCAAAACCAGAAG

GAGCTTCATATATTACTGAGACTGGACAAGATGGAGTACGGGCGGAACGTGATACGCATTTCACATTTCCTCAACGCAAACAATTTCCCCGATACTCAGC

TGTCTGAAGCTCGTCTGTCCTTTGCGAAAACCATGACTCTCGTCACTCGTTTGGATGATTTCTTCGATCACCATGGCTCTAGAGAAGATTCTCTCCTCAT

CATTGAATTAATAAGACAGTGGAATGTGCCATCAACAATAAGATTCCCCTCCGAAGAAGTTGAGATTCTCTACTCTGCACTTCACTCCACAGTAACAGAC

ATAGCAGAGAAGGCTTATCCCATGCAGGGCCGCTGCATCAAATCACTCATAATTCATCTGTGGGTTGAGATATTGACGAGTTTCATGAGAGAAATGGACT

CATGCACTGCGGAACTTCCACCAAACATGCATGAGTACTTAGGGTTTGCATGGATCTCCATCGGCTGCAGAATTTGCATTCTTGTAGCCATCCATTTCTT

GGGGGAGAAGGTATCCGAAGAACTGGTTATGAGTGCAGAGTGCACCGAGTTATGCCGGCACGTTTCTTCAGTCGCACGCCTTCTCAACGATCTCCAAACC

TTTAAGAAGGAGAGAGAAGAGAGGAAGGTAAACAGTGTGATAATTCAGCTCAACGGTGACGAGAAGATACCGGAGGAGGAGGCGGTATCGAATGTAGAGA

AAATGATTGAATTTCACAGGAAAGAGCTGCTGAAGATGGTGGTGCAGAGAGAAGGAAGCTTGGTTCCTAAAAAATTGAATAATGAGGATCTATTTCTATT

GTCGGCGCAAGATTTCATGGCTCGCCAAGCTCAAAACCTAAAGGAGCTTCCATTGTTGTTGAGCGCAAACAATTTACCCGATCCTCAGCTGTCCGAGGCT

CGTCTGGCCTTCGCGAAAACCATGAATCTCGTCGCTCGTCTGGATGATTTCTTCGATCACCGCGGCTCCAGAGAAGATTGTCTCCTCATCATTGAATTAA

CAAGGCAGTGGAATGAGCCTTCAACAATAAGATTCCCCTCCGAAGAAGTTGAGATGCTCTACTCTGCACTTCACTCCACTGTAACAGATATAGCAGAGAA

GGCTTATCCCATGCAAGGCCGCTGCATCAAATCACTCCTAATTCATCTGTGA

>MlongTPS46

ATGTCGCTAGCGTTTTACACCGGAGTGACCGCTTTCTCCGGCCACACAGTTCGGAGGAGGAAAGAAATTTTTCCAGGTCACCGTCAAATTACAGTCCACG

GATTTTCGACGATGAGATCTCCGGTCACCGTCAAATGCAACGTTAGCCCAACGGATTTGATGGAGAAAATAGGGAAGAAAGTCGAGGGCAAAAACGGTAA

TATATCTCTAGTTGGCGCGAATAATGAAGGTGAAGATATAGCCTTTAATCTGTGTATAGTCGACACCCTCCAAAGGTTGGGAGTCGACCGTTACTTCCGA

TCTGAAATCCATACTATTCTTGAAGACACGTTTAGGTTGTGGCTACGTAAAGAGAAAGAAATATATTCTGATATTACTATTCATGCAATGGCATTTAGGC

TTTTGAGAGTCAAAGGATATCCAGTCTCTTCCGTTATCGAGCTGTACAGAGCATCGGAGGAGAGAATTTATGAAGAAGAGAGGAGTCTTGAGAAACTCCA

TGCTTGGACCACCACCTTTCTCATGCACCAGCTGCAGACTAACTCAATTCCTGATCAAAAAATGCATAAACTGGTGGAATACTACTTGAAGAACTACCAT

GGCATATTAGATAGGGTGGGAGTTAGACGAAACCTCGAGCTATACGACATAAGCCATTATCCAACTCTAAGAGTAAGGCTTCCGAACCTATGTAATGAAG

ATTTTCTATCATTCGCGAAGCAATATTTCAATATTTGCCAAGCCCAACACCAGAAAGAACTTGAGCAACTACAGAGGTGGTTTGCAGATTGCAGATTCGA

CACCTTGAAGTTTGGAAGAGATACAGTAGCCGCTGCTAATTTTCTGACTTCAGCAATTATCAGTGAACCTGAATTATCTCATGTTCGTCTAGCCTTCGCC

AAACATATGATGCTTGTGGTATATATCGATGATTTTTTCGATCAGTACGGATCAAGAGAAGACTCCTTCAAGATCCTCCAATTAATCAAAGAATGGAAAG

AGAAGCCAGCTGCAGAATATGGTTCTGAGGAAGTCGAAATCCTCTTCACAGCAGTGTACAATACAGTGAATGAGATTGCAGAAATGGCTGGTGTTGAACA

AGGGCGCAGCGTCAAAGATTTTCTTATCCAACTGTGGGTGGAATTTCTATCAATGTGCATGGTAGAGTTGGACACGTGGAGCGACGAGACAGCAGTAACC

TTGGATGAGTACTTGTCTTCTTCATGGGTTTCAATCAGTTGCAGACTCTGCATTCTCATGTCCATGCAATTCATGGGACTCAAACTGTCTGATGAAATGC

TTATGAGTGAAGAGTGCATTGATTTATGTAGGCATGTTTCTATAGCTGATCGTCTACTAAACGACGCCCACACTTTTGAGAGGGAACGCGAGGAAAAGAA

AGGGAATAGTGTGACTATTCTCATAGCAGCTGGGAATGGTGAAAGGGCATTGAGTGAAGAGGAAGCTATTGTTGAAATAAAAGAAATGGCGGAATGCGAG

AGGAGGAAACTGATGCAGATTGTGTATAAAAGAGGAAGTGTTTTCCCAAGAGAATGCAAGGACATGTTCTTGAATGTATGCAGAATTGGGTGTTATTTGT

ACTCGAGCGGCGACGAATTCACATCTCCTCAACAAATGAAGGAAGACGTCAAGTCCTTGATTTATGAACCCTTAACACTTCATAATTAG

>MlongTPS47

ATGTATGCATTGAATGTTATTCCCCTCTCAAGCTCTATTGTCGGGTTTTCGGGTTGTCGGATCCTCCGTACCAAATGCAGCTTGCACCTCGATGTTCCGG

TGGCCGAGAAGAAGCTGCAGGTTGATAAAGTTAGAGAGAGGATAACAAATGGGAAGGCGGAGATGTCGCCGTCGGCGTACGACACGGCGTGGGTGGCGAT

GGTGCCGTCGAGGGAATATTCCAGCAGGCCGGGTTTCCCGGAGTGCTTGGAATGGATAAAGGAGAATCAGAATCCGAACGGGTCGTGGGGTCTGGATCCG

GGTCACCGGTTTCTGGTGAAAGATTCCCTCTCATGCACCTTGGCATGCCTACTTGCCCTGCGCAAATGGAACACAGGCAACCAACTTGTCCAAAGGGGTC

TGGAGTTCATGGAGTCCCATGGTTGGGCTGCAACTGATGAGAACCAGCTTCCTCCTATTGGATTCGATATCCTATTTCCTGCTATGATTGATTATGCCAA

TGAACTGGATCTAACTCTGCCTTTTAACCCAACTTCACTTCATTCATTGCTAAATTTTAGAGATTCTGAATTAAGAAAAGGAAATTGGGAATATGTCGGT

GAAGGAGTAGTAGACGATTCAAGAAATTGGAAGAAAATAATAAGCAGCCATCAAAGAATTAATGGCTCCTTGTTCAATTCACCTGCTACCACAGCTGCCG

CTCTTATTCACACTCACGACGATAAATGCTTCCATTATTTGCTCTCTGTTCTTCATAATTCCAACGGATGGGTACCTACTATGTATCCCATGGATTTATA

CGCTCGTCTGTGCATGATCGATAAGCTTCAAAGGTTAGGAATAGACCGATACTTCAAAGCTGACCTCACCCCCATTTTCGATGAGATATACAGGAATTGG

GAAGAGAGAGAAGATGAAATATTTTCGAACATTACTTGTCTAGCCCTAGCATTTCGGCTTCTACGAATGCAAGGATATGATGTTTCATCTGATGAGCTGG

CAAAATTTGTGGAGGAGGAAGGTTTCTTGAATGGAGTGAGCATGGAGCAAAGAGGTACAGGCACAGTGCTTGAGCTTTACAGAGCTTCACATATAAGAAT

GAAGGAAGAAGAAATAATTCTCGACAAAATTAATGCTTGGACTAAACCATTTCTCAAGCTCCAGCTTCTTAACCGTAGCATTCGCGACAAACGATTGGAG

AAGCAGGTGGAATACGACTTGAAGAACTTGTACGGTGCACTAGACCGGTTTCAGCACAGACGAACCATAGATTTATACGATCCTAAATCTTCCCCAATGG

CCAAAACATCATACAGGTTCTCCGCAGTTCATAATGAAGACTTATTTCATTTCGCTGTTGAAGACTTCAAACTCTCCCGAGCAGAATACCTTAAAGAACT

TGAACAAATGAAGAAGTTGGACCTCTTAACAAAGGGAAGAAATGCATGTAGAGAATCTTACATTTTAACCGCTGCTCTTATAGTTGATCCTCAGCTGTCC

ATGGCTCGAATGACTTATGCTAAAGTTGCTATCCTCATCACCAATTTCGATGACTTTTTCGATCATTATGGCTCTAAAGAAGAAGCCTTCGATATTATTC

ATCTCATAAAGGAATGGAAGCTAGCAGACAGCTACAGCTCTAAAGAAGTGGAGATTCTATTCACTGCGCTATACCACACCATAAACGACGTCGCAGCCAA

GGCTGATGCAGAGCAAGGCTTTTGTTCCAAACAACAACTTATCAACCTGTGGGTGGAGCTGTTAGAGAGCGCAGTGAGAGAAAAGGACTCATTGAGCGGA

AGGAACATGACGACTCTAGAGGAGTACTTGTCTTTCGCTCCGACTACCATCGGTTGCAGAAGCTGCGTGATGACTTCCGTCCATTTCCTCGGAGTCAAAC

TCTCCGAGAAAATCTGGACCTCTGAGGAGCTGAGCAGTCTCTGCAAACACGGCGCTGTTGTTTGCAGACTGCTCAACGACCTCAAGACCTACAAGAGAGA

ACGTGAAGAGGGGACGCTCAACAGCGTGAGTGTGCAGATGGTGGGAGGAGGCGTTTCGGAGGAGGAGGCGGTGGCGAAGGCGGAGGAGGTGATGGAGTAC

CATCGGAGGAAAGTGATGGAGGTTGTGTATGGAAGAAAAGGGAGAAGTAGTGTTCCTAGAGAGTGCAAAGAGCTAGTGTGGAAGACGTGCAAGCTTGCCT

ATTGCCTGTACGGTCACGACGGAAGCGACGAATTCTCATCCCCCAAAGATATTCTCAAGGATATTAACGCTATGATGTTTGAGCCTCTACCATGA

>MlongTPS48

ATGTATCCCATGGATATCTACGCTCGTCTCTGCATGATCGATACACTTGAAAGATTAGGAATAGACCGATACTTCAAAGTTGAGCTCACCGCCATTTTTG

ATGAGATATACAGGTTCTCTGCAGTTCATAATGAAGACTTATTTCATTTCGCTGTTGAAGACTTCAAACTCTCCAGAGCAGAATACCTTAAAGAACTTGA

ACAAATGAAGAAGTGGTACTCTGATTGTAGGTTGGACCTCTTAACGAAGGGAAGAAATGCATGTAGAGAATCTTACATTTTAACCGCTGCTCTTATAGTT

GATCCTCACCTATCCATGGCTCGAATGACTTATGCTAAAGTTGCTATCCTCATCACCAATTTCGACGACTTTTTCGATCATTATGGCTCTAAAGAAGAAG

CCTTCGATATTATTCATCTCACAAAGGAAGTTGCATTATCATATGCTAGATGGAAGCTAGCAGACAGCTACAGCTCTAAAGAAGTGGAGATTCTATTCAC

TGCGCTATACCACACCATAAACGACGTCGCAGCCAAGGCTGATGCAGAGCAAGGCTTTTGCTCCAAACAACAACTTATCAACCTGTGGGTGGAGCTGCTA

GAGAGCGCAGTGAGAGAAAAGGACTCATTGAGCGGAAGGAACATGACGACTCTGGAGGAGTACTTGTCTTTCGCTCCGACTACCATCGGTTGCATAAGCT

GCGTGATGACTTCTGTCCATTTCCTGGGAGTCAAACTCTCCGAGGAAATCTGGACCTCTGAGGAGCTGAGCAGTCTCTGCAAACACGGCGCTGTTGTTTG

CCGACTGCTCAACGACCTCAAGACCTACAAGAGAGAGCGTGAAGAGAAGACGCTGAACAGTGTGAGCGTGCAGATGGTGGGAGGCAGCGTTCCGGAGGAG

GAGGCGGTGGCGAAGGCGGAGGAGGTGATGGAGTACCATCGAAGAAAAGTGATGGAGGTTGTTTATGGAGGAAAAGGCAGCCGTAGTAGTGTTCCTAGAG

AGTGCAAAGAGCTAGTGTGGAAAACGTGCAAGCTTGCCTATTGCTTGTACGGTCACGATGGAAGCGATGAGTTCTCTTCCCCCAAAGATATTCTCAAGGA

CATTAACGCTATGTTGTTTGAGCCTCTACCGTGA

>MlongTPS49

ATGTATCCCATGGATATCTACGCTCGTCTCTGCATGATCGATACACTTGAAAGATTAGGAATAGACCGATACTTCAAAGTTGAGCTCACCGCCATTTTTG

ATGAGATATACAGGAATTGGGAAGAGAGAGAAGATGAAATATTTTCGAACATTACTTGTCTAGCCCTAGCATTTCGGCTTCTACGAATGCAAGGATATGA

TGTTTCATCTGATGAGCTGGCAGAATTTGTGGAGGAGGAAGGTTTCTTGAATGGAGTAAGCATGGAGCAGAGAGGTGCAGGTACAGTGCTTGAGTTTTAC

AGAGCTTCACATATAAGAATGAAGGAAGAAGAAATAATTCTCGACAAAATTAATGCTTGGACTAAACCATTTCTCAAGCACCAGCTTCTTAACCGAAGCA

TTCGCGACAAACGATTGGAGAAGCAGGTGGAATACGACTTGAAGAACTTGTACGGCGCACTAGACTGGTTCCAGCACAGACGAACCCTAGATTTATACGA

TCCTAAATCTTCCCGAATGGCCAAAACATCATACAGGTTCTCTGCAGTTCATAATGAAGACTTATTTCATTTCGCTGTTGAAGACTTCAAACTCTCCAGA

GCAGAATACCTTAAAGAACTTGAACAAATGAAGAAGTTGGACCTCTTAACGAAGGGAAGAAATGCATGTAGAGAATCTTACATTTTAACCGCTGCTCTTA

TAGTTGATCCTCACCTATCCATGGCTCGAATGACTTATGCTAAAGTTGCTATCCTCATCACCAATTTCGACGACTTTTTCGATCATTATGGCTCTAAAGA

AGAAGCCTTCGATATTATTCATCTCACAAAGGAAGTTGCATTATCATATGCTAGATGGAAGCTAGCAGACAGCTACAGCTCTAAAGAAGTGGAGATTCTA

TTCACTGCGCTATACCACACCATAAACGACGTCGCAGCCAAGGCTGATGCAGAGCAAGGCTTTTGCTCCAAACAACAACTTATCAACCTGTGGGTGGAGC

TGCTAGAGAGCGCAGTGAGAGAAAAGGACTCATTGAGCGGAAGGAACATGACGACTCTGGAGGAGTACTTGTCTTTCGCTCCGACTACCATCGGTTGCAT

AAGCTGCGTGATGACTTCTGTCCATTTCCTGGGAGTCAAACTCTCCGAGGAAATCTGGACCTCTGAGGAGCTGAGCAGTCTCTGCAAACACGGCGCTGTT

GTTTGCCGACTGCTCAACGACCTCAAGACCTACAAGAGAGAGCGTGAAGAGGAGACGCTCAACAGCGTGAGCGTGCAGATGGTGGGAGGAGGCGTTTCGG

AGGAGGAGGTGGTGGCGAAGGCGGAGGAGGTGATGGAGTACCATCGGAGGAAAGTGATGGAGGTTGTGTATGGAAGAAAAGGGAGAAGTAGTGTTCCTAG

AGAGTGCAAAGAGCTAGTGTGGAAGACATGCAAGCTTGCCTATTGCTTGTACGGTCACGATGGAAGCGATGAGTTCTCTTCTCCGAAGGATATTCTCAAG

GACATTAACTCCATGATGTTTGAGCCTCTACCATTATTGGAAATGGCTATCGCCGCCGCTAGATCCGTTTTCCGGTCGTCAAACTTTCGTAGCGCCGCCA

CGTGGCTGGCGTCGCAATCGAAGTCGGCTCCCTCGTCGCCATTCCGCCTCGCCTTCAAATCTCCTTTACTAGCTCATCGCATCTTCCGGTGGCCTGCTGA

ATTTGGCACGTGCTTGGAGTCGATGCAGTTTTAG

>MlongTPS50

ATGTATCCCATGGATATCTACGCTCGTCTCTGCATGATCGATACACTTGAAAGATTAGGAATAGACCGATACTTCAAAGTTGAGCTCACCGCCATTTTTG

ATGAGATATACAGGAATTGGGAAGAGAGAGAAGATGAAATATTTTCGAACATTACTTGTCTAGCCCTAGTATTTCGGCTTCTACGAATGCAAGGATATGA

TGTTTCATCTGATGAGCTGGCAGAATTTGTGGAGGAGGAAGGTTTCTTGAATGGAGTAAGCATGGAGCAGAGAGGTGCAGGTACAGTGCTTGAGCTTTAC

AGAGCTTCACATATAAGAATGAAGGAAGAAGAAATAATTCTCGACAAAATTAATGCTTGGACTAAACCATTTCTCAAGCACCAGCTTCTTAACCGAAGCA

TTCGCGACAAACGATTGGAGAAGCAGGTGGAATACGACTTGAAGAACTTGTACGGCGCACTAGACTGGTTCCAGCACAGACGAACCCTAGATTTATACGA

TCCTAAATCTTCCCGAATGGCCAAAACATCATACAGGTTCTCTGCAGTTCATAATGAAGACTTATTTCATTTCGCTGTTGAAGACTTCAAACTCTCCAGA

GCAGAATACCTTAAAGAACTTGAACAAATGAAGAAGTGGTACTCTGATTGTAGGTTGGACCTCTTAACGAAGGGAAGAAATGCATGTACAGAATCTTACA

TTTTAACCGCTGCTCTTATAGTTGATCCTCACCTATCCATGGCTCGAATGAGTTATGCTAAAGTTGCTATCCTCATCACCAATTTCGACGACTTTTTCGA

TCATTATGGCTCTAAAGAAGAAGCCTTCGATATTATTCATCTCACAAAGGAATGGAAGCTAGCAGACAGCTACAGCTCTAAAGAAGTGGAGATTCTATTC

ACTGCGCTATACCACACCATAAACGACGTCGCAGCCAAGGCTGATGCAGAGCAAGGCTTTTGCTCCAAACAACAACTTATCAACCTGTGGGTGGAGCTGC

TAGAGAGCGCAGTGAGAGAAAAGGACTCATTGAGCGGAAGGAACATGACGACTCTGGAGGAGTACTTGTCTTTCGCTCCGACTACCATCGGTTGCATAAG

CTGCGTGATGACTTCTGTCCATTTCCTCGGAGTCAAACTCTCCGAGGAAATCTGGACCTCTGAGGAGCTGAGCAGTCTCTGCAAACACGGCGCTGTTGTT

TGCCGACTGCTCAACGACCTCAAGACTTACAAGAGAGAGCGTGAAGAGAAGACGCTGAACAGTGTGAGCGTGCAGATGGTGGGAGGCAGCGTTCCGGAGG

AGGAGGCGGTGGCGAAGGCGGAGGAGGTGATGGAGTACCATCGAAGAAAAGTGATGGAGGTTGTTTATGGAGGAAAAGGCAGCCGTAGTAGTGTTCCTAG

AGAGTGCAAAGAGCTAGTGTGGAAAACGTGCAAGCTTGCCTATTGCTTGTACGGTCACGATGGAAGCGATGAGTTCTCTTCCCCCAAAGATATTCTCAAG

GACATTAACGCTATGTTGTTTGAGCCTCTACCGTGA

>MlongTPS51

ATGTATGCATTGAATGTTATTCCCCTCTCAAGCTCTATTGTCGGGTTTTCGGGTTGTCGGATCCTCCGTACCAAATGCAGCTTGCACCTCGATGTTACGG

TGGCCGAGAAGAAGCTGGATGATAAAGTTAGAGAGAGGATAACGAATGGGAAGGCGGAGATGTCGCCGTCGGCTTACGACACGGCGTGGGTGGCGATGGT

ACCGTCGAGGGAATATTCCAGCAGGCCAGGTTTCCCGGAGTGCTTGGAATGGATAAAGGAGAACCAGAATCCGAACGGGTCATGGGGACTGGATCCGGGT

CACCCGTTTCTGGTGAAAGATTCCCTCTCATGCACCTTGGCATGCCTACTTGCCCTCCGTAAATGGAACACAGGCAACCACCTTGTCCAAAGGGGTCTGG

AGTTCATGGAGTCCCATGGTTGGGCTGCAACTGATGAGAACCAGCTTCCTCCTATTGGATTCGATATCCTATTTCCTGCTTTGATTGATTATGCCAATGA

ACTTGATCTAACTCTGCCTTTTAACCCAACTTCACTTCATTCATTGCTAAACTTTAGAGATTCTGAATTAAGAAAAGGAAAGTGGGAATATGTAGGTGAA

GGAGTAGTAGACGATTCAAGAAACTGGAAGAAAATAATAGGCAGCCATCAAAGAATTAATGGGTCCCTGTTCAATTCACCTGCTACCACCGCTGCCGCTC

TTATTCACACTCACGACGATAAATGCTTCCATTATTTGCTCTCCGTTCTTCAATATTCCAACGGATGGGTACCTACTATGTATCCCATGGATATCTACGC

TCGTCTCTGCATGATCGATACACTTGACAGATTAGGAATAGACCGATACTTCAAAGTTGAGCTCACCGCCATTTTTGATGAGATATACAGGAATTGGGAA

GAGAGAGAAGATGAAATATTTTCGAACATTACTTGTCTAGCCCTAGCATTTCGGCTTCTACGAATGCAAGGATATGATGTTTCATCTGATGAGCTGGCAA

AATTTGTGGAGGAGGAAGGTTTCTTGAATGGAGTGAGCATGGAGCAAAGAGGTACAGGCACAGTGCTTGAGCTTTACAGAGCTTCACATATAAGAATGAA

GGAAGAAGAAATAATTCTCGACAAAATTAATGCTTGGACTAAACCATTTCTCAAGCTCCAGCTTCTTAACCGTAGCATTCGCGACAAACGATTGGAGAAG

CAGGTGGAATACGACTTGACGAACTTGTACGGTGCACTAGACCGGTTCCAGCACAGACGGACCATAGATTTATACGATCCTAAATCTTCCCCAATGGCCA

AAACATCATACAGGTTCTCTGCAGTTCATAATGAAGACTTATTTCATTTCGCTGTTGAAGACTTCAAACTCTCCCGAGCAGAATACCTTAAAGAACTTGA

ACAAATGAAGAAGTTGGACCTCTTAACAAAGGGAAGAAATGCATGTAGAGAATCTTACATTTTAACCGCTGCTCTTATAGTTGATCCTCAGCTGTCCATG

GCTCGAATGACTTATGCTAAAGTTGCTATCCTCATCACCAATTTCGACGACTTTTTCGATCATTATGGCTCTAAAGAAGAAGCCTTCGATATTATTCATC

TCATAAAGGAATGGAAGCTAGCAGACAGCTACAGCTCTAAAGAAGTGGAGATTCTATTCACGGCGCTATACCACACCATAAACGACGTCGCAGCCAAGGC

TGATGCAGAGCAAGGCTTTTGTTCCAAACAACAACTTATCAACCTGTGGGTGGAGCTGTTAGAGAGCGCAGTGAGAGAAAAGGACTCATTGAGCGGAAGG

AACATGACGACTCTAGAGGAGTACTTGTCTTTCGCTCCGACTACCATCGGTTGCAGAAGCTGCGTGATGACTTCCGTCCATTTCCTCGGAGTCAAACTCT

CCGAGAAAATCTGGACCTCTGAGAAGCTGAGCAGTCTGTGCAAACACGGCGCTGTTGTTTGCAGACTGCTCAACGACCTCAAGACCTACAAGAGAGAACG

TGAAGAGGGGACGCTCAACAGCGTGAGTGTGCAGATGGTGGGAGGAGGCGTTTCGGAGGAGGAGGCGGTGGCGAAGGCGGAGGAGGTGATGGAGTACCAT

CGGAGGAAAGTGATGGAGGTTGTGTATGGAAGAAAAGGGAGAAGTAGTGTTCCTAGAGAGTGCAAAGAGCTAGTGTGGAAGACGTGCAAGCTTGCCTATT

GCCTGTACGGTCACGACGGAAGCGACGAATTCTCATCCCCCAAAGATATTCTCAAGGATATTAACGCTATGATGTTTGAGCCTCTACCATGA

>MlongTPS52

ATGTATCCCATGGATATATACGCTCGTCTTTGCATGATCGATACACTTGAAAGATTAGGAATAGACCGATACTTTGGAGTTGAGCTCAACGCCATTTTTG

ATGACATATACAGGAATTGGGAAGAGAGAGATGAAGAAATCTTTTCTAACGTTACTTGTGTAACCCTCGCATTTCGACTTCTACGAATGAAAGGATATGA

TGTTTCATCCGATGAGCTGGCAGAATTTGTGGAGGAGGAAGGTTTCTTGAATGGAGTAAGCATGGAGCAGAGAGGTGTAGGCACAGTGCTTGAGCTTTAC

AGAGCTTCACATATAAGAATGAAGGAAGAAGAAATAATTCTCGACAAAATTAATGCTTGGACTAAACCATTTCTCAAGAACCAGCTTCTTAACCGTAGCA

TTCGCGATGAACGATTGGAGAAACAGAACTTGTACGGCGCACTAGACCGGTTCCAGCACAGACAAACCCTAGATTCATACGATCCTAAATCTTCCCACAT

CGCCAAAACATCATACAGGTGCTCTGCAGTTTACAACGACGACTTCCTCCGTTTCTCCGTTGAAGACTTCAGAATCTCCCGAGCGGAATACCTAAAGGAA

CTTGAACACATGAATAAGTGGTACTCTGATTGTAGGTTGGATCTGTTGAGTGAGGGAAGGAATGCATGTAAGCATTCTTACATTTTAACAGCTGCAGTAA

TTGTTGATCCTCAGCTATCCATGGCTCGAATCTCTTACACTCAAGTTGTGCTCCTCATAACTGTTTTGGACGACTTTTTCGACAATTATGGCTCTAAACA

AGAAGCATTCACTATTATTCATCTAATAAAGGAATGGAAGCTAGCAAAGAACTACTGCTCCAAAGAGGTGGAGATAATATTCACTGCTCTATACCACACC

ATAAACGGAATTGCAGCAAAGGCTCATGCAGAGCAAGGCTTTTGTTCCAAACAACAACTTATCAACTTGTGGGTGGAGCTACTCGAGAGCGCGGCGAGAG

AGAAGGACTCACTGAGCGGAAACCGAGTCGCGACTCTCGAGGAGTACTTATCCTTCGCCCCCGATACGATCGGATGCAAGACCTGCGTCATGACGTCCGT

TCATTTCCTCAGAATCAGACTTTCCGAGGAAATTTGGACTTCTGAGGAGTTGAGCAGTCTGTGCAAACACGGCGACGTCGTTTGCAGACTGCTCAACGAC

CTCAAGACCTACAAGAGAGAGCGCGAAGAGAAGACGCTCAACAGTGTGAGCGTGCAAATGGTGGGAGGCGGGGCCTCGGAGGAAGAGGCGGCGGCGAAGG

TGGAGGAGGTGTTGGAGTTCCATAAAAGAAAATTAATGCAGATTGTGTACGGAAGAAAAGGGAGCAGCAGTGTTGCTAGAGAGTGTATGGAGCTAGTGTG

GAAATCATGCAAGCTTGCTTATTGCTTCTACGGTCACGATGGAGGCGATGAGTTCTCTTCACCCCATAATATTCTCAAGGATATTAATGACATGATGTTT

GAGCCCTTACCATGA

>MlongTPS53

ATGACAATCGCCTTCAAGCTCAGATCAATTATCCCTTTCTCTGGCCATAGTGTCTGGAGTAGGAGACACCAAATATTTCCAGTTCACGGATTTCCGGTGA

TGATCGCTACCAATAAGTCGTCTCTCACCGATGAATGCAACCTTAGGTCGACGGATTTGATGGGCAAAATGGGAACAAACTTGACGGCCAAAAAGGGTAA

TATAATTCAAGTTGGTGCGAATATTGAAGCTGGGGACATAGCCACCAACCTCTGCATAATCGACACCCTCCAAAGACTGGGAGTGGACCGTTACTACCAA

ACTGAGATCGATTCTGTTCTAGAGGACACCTACAGGCTATGGCAGCGGAAAGATAAAGATATATATTCGGACATTAGTATTCATGCAATGGCGTTCAGAC

TTTTGCGAGTCAAAGGATATCAAGTCTCTTCAGAGGAACTGGCTCCATATGTTGATGAAGAGCGAATGAATCTGCAAACAATCGAGGTGGAAACAGTTGT

CGAGCTGTACAGAGCATCGGAGGAGAGAATGTATGAAGAAGAGAGCATTCTTGAGAAACTCCATGCTTGGACCACCACCTTTCTCAAGCACCAGTTGCAG

ACTAACTCCATTCCTAACCACAAATTGCACAAGCTTGTGGAATACTATTTGAACAACTACCACGGTATAATACATAGAATTGGAGTTAGAAGAAACCTCG

ACCTCTTCGACACAAGCTATTATCAAACTCCAACAGCTCCAGATAGGTTCTCTAATCTATGCAATGAAGATTTCCTAGCATTCGCGAGGCAAGATTTTAA

TATATGCCAAGCCCAACTCCAGAAAGAACTTGAACAACTGCAAAGGTGGTATGCAGAATGTAGGTTGGATACCATGGAGTTTGGAAGAAATGTAACTCGC

ATTGCTCATTTTATGGCTTCAGCAATTGTTAATGGTGATCCTAATTTTTCCGAAGCTCGACTAGCCTTAGCCAAACATGTTGTCCTCGTTACGCGTGCCG

ATGATTTCTTTGATCATGGTGGGACTAGAGAAGAGTCCTACAAGATCATTGAATTAGTAAACGAGTGGAAAGAGAAGCCAGTAGCAGAATATGGTTCTGA

GAAAGTTGAAATCATGTTCACAGCAATATACAATACAGTGAATGAGATTGCAAAGATGGCTCACATCGAACAAGGGCGCAGTGTCAAAGAATTTCTAATC

GGAATGTGGGTTGAGATACTGTCGATCTTCAAGATGGAACTAGACACGTGGAGCGACGAGACGGAAATAAGCTTGGATGAGTACATGTCTTCCTCATGGG

TTTCCATTGGTTGCAGAATCTGCAGCCTCATGTCGATGCAATTCATTGGTGTAAAATTATCCGATGAGATGCTTCTGAGTGAAGAGTGCAATGATATGTG

CAAGCATCTTTCTCTAGTTGATCGCCTCCTCAACGACGTGCAAACTTTTGAGAAGGAACGCAACGAGAATACAGGAAGCAGCGTGACAATTCTGGTGGCG

GCAGCTGAGAGAGATGGAAGAGTTGGTAGTGAAGAGATAGCTGTAAGAAAGATAAGAGAAATAGTTGAATATAACAGGAGGAAATTGATGCAGATTGTGT

ATAAAAGAGGAAGTGTTTTCCCAAGAGAATGCAAGGATATATTCTTGAATTCATGCAGGGCTGGTTGTTATGTGTACGGCAGCAGCGATGAATTTTCTTC

TCCTCAACAATTGATGATGGAAGATATGAAATCCTTGATTCATGAACCTGCTTAA

>MlongTPS54

ATGGAGAAAACACGAGAGGAGTTCAAGGGGCAAGTCAGTAATTCTCCGATAGCCCCGGCTCTTCGACTTTCGGATATACCCTCTAGTCTGTGTATAATCG

ACACAGTCGAAAGGTTGGGAATCGACCGCTACTTCCAATCTGACATCGATAATGTTCTAGAGCACACATACAGGCTATGGCAACAGAAAGACAAAGATAT

ATATTCCGATGTTACTACTCATGCAATGGCGTTTAGACTTTTGCGAGTCAGAGGATATGAGGTTTCATCAGAGGAGTTGGCTCCATATGCTCACTTGAGC

CAGGAAACAATAGATGTGGCGATGGCTATAGAGCTATTCAGAGCAGCACAAGAGAGAATATACGAAGACGAGAGCAGTCTCGACAAACTACTTGCCTTCA

CCTCTGCTTTTCTCAAGAACCAGCTGCTCACTAACTCCATTCTTGACAACAAATTGCATAAACTGGTGGAATACTACTTGAATAACTACGACGGCAGAAT

AGGTAGAATGGGAGTTAGACTAAACATCGACGTGTATGACATGAGCCATTATCAAACTCTAAAAACTTCACATAGGTTGTATAATCTATGTAATGAAGAC

TTTCTAGCATTTGCAAGGCAAGATTTCAATAAGTTTCAATCCCAACAGCAGAAAGAGCTTGAGCAACTACAAAGGTGGAATGCAGATTGTGGGTTGGACA

AGTTGAAGTATGGAAGAGATGTTGTAAGGATTTGTAATTTCTTGTGTTCATCGATGGTCGAAGAACCTGAATTATCTGAAGTTCGTCTATCTATGGCCAA

ACAATTTGTGCTTTTAACACGTGTTGATGATTTCTTCGATCTTGCTGGCTCTAAACAAGAATCCTACAAGATCATTGAATTAGTAAAGGAATGGAAAGAG

AATCCAACTACAGAATATGATTCCGAGGAAGTTAAAATCCTTTTTACAGCAGTATACAACACAGTAAATGAGGTGGCAGAGAAGGCTCATGTTCAACAAG

GACGTAACGTCAAAGAATTTCTAGTTAAACTGTGGGTTGAGATACTATCAGCTTTCAAGATGGAATTAGATACATGGAGCGATGGTACGGAAGTAAGCTT

GGATGAGTACTTGTCGTGGTCGTGGCTTTCGATTGGCTCCAGACTATTTATAGTAACCTCCATGCATTTGTTCCCTGCAAAATTATGCAACCAAGAAATG

CTTATGAGTGAAGAGTGCGCTGATTTGTGTAGGCATGTTTCAATAGTTTGTCGCCTTCTCAATGATATCCGTTCTTTTGAGGTAATTAATGTTCCCGTGT

CCAATCGAGTCATAAATGGATCGAACTTTGACATGATATTCAGTTCATAA

>MlongTPS55

ATGGAGAAAACAAGAGAGGAGTTCAAGGGGCAAGTCGATAATTCTCCGATAGCCCCGGCTCTTCGACTTTCGGATATACCTTCTAGTCTGTGTATAATCG

ACACTGTTGAAAGGTTGGGAATCGACCGCTACTTCCGATCTGACATCGATAATGTTCTAGAGCACACATACAGGCTATGGCAACAAAAAGACAAAGATAT

ATATTCCGATGTTACTACTCATGCAATGGCGTTTAGACTTTTGCGAGTCAGAGGATATGAGGTTTCATCAGAGGAGTTGGCTCCATATGCTCACTTGAGC

CAGGAAACAATAGATGTGGCGATGGCTATAGAGCTATACAGAGCAGCACAAGAGAGAATATACGAAGACGAGAGCAGTCTCGACAAACTACTTGCCTTCA

CCTCTGCTTTTCTCAAGAACCAGCTGCTCACTAACTCCATTCTTGACAACAAATTGCATAAACTGGTGGAATACTACTTGAATAACTACGACGGCAGAAT

AGGTAGAATGGGAGTTAGACTAAACATCGACGTGTATGACATGAGCCATTATCAAACTCTAAAAACTTCACATAGGTTGTATAATCTATGTAATGAAGAC

TTTCTAGCATTTGCAAGGCAAGATTTCAATAAGTTTCAATCCCAACAGCAGAAAGAGCTTGAGCAACTACAAAGGTGGAATGCAGATTGTGGGTTGGACA

AGTTGAAGTATGGAAGAGATGTTGTAAGGATTTGTAATTTCTTGTGTTCATCGATGGTCGAAGAACCTGAATTATCTGAAGTTCGTCTATCTATGGCCAA

ACAATTTGTGCTTTTAACACGTGTTGATGATTTCTTCGATCTTGCTGGCTCTAAACAAGAATCCTACAAGATCATTGAATTAGTAAAGGAATGGAAAGAG

AATCCAACTACAGAATATGATTCCGAGGAAGTTAAAATCCTTTTTACAGCAGTATACAACACAGTAAATGAGGTGGCAGAGAAGGCTCATGTTCAACAAG

ACTGGGTTGAGATACTATCAGCTTTCAAGATGGAATTAGATACATGGAGCGATGGTACGGAAGTAAGCTTGGATGAGTACTTGTCGTGGTCGTGGCTTTC

GATTGGCTCCAGACTATTTATAGTAACCTCCATGCATTTGCTCCCTACAAAATTATGCAACCAAGAAATGCTTATGAGTGAAGAGTGCGCTGATTTGTGT

AGGCATGTTTCAATAGTTTGTCGCCTTCTCAATGATATCCGTTCTTTTGAGAGGGAACGCGAGGAAAAAACAGGAAACAGTGTGAGCATTCTAGTAGGGG

ATGGAAGAGGTGTTAGTGAGGAGGAAGCTATTACAAAGATAAAAGAAATAGTTGAGTATCACAAGAGGAAACTGATGCAGATTGTGTACAAAAGAGGAAC

CATTTTCCCAAGAGAATGCAAGGACATGTTTCTGAAGACATGTAGGGCTATTTCTTATTTGTACTTGAGCACCGACGAATTCACGTCGCCTGAGCAAATG

AACGAAGATATGAAACGTCTAAGTTGTCCTTGA

>MlongTPS56

ATGGGGAAAGCAAAAGAGAAGTTCGAGGGGCAAATCGGTAATTCTCCGATAACCCCGGCTCTTCAACTTGCAAATATACCCTCTAGTCTGTGCATAATCG

ACTCCCTCGAAAGGTTGGGAATCGACCGATACTTCCGACGTGAAATTGACATTGTTCTAGAGAACACATACAGGCTATGGAAAGAGAAAGACAAAGATAT

ATATTCCGATGTTACTACTCATGCAATGGCGTTTAGACTCTTGCGAGTCAAAGGATACAGGGTTTCATCAGAGGAACTGGCCCCATATGCTAACCAAGAG

CGCTTCAGCCATCAAAAGATTGATGTGGCGATGACTATAGAGCTTTACAGAGCAGCACAAGAGAGAATATACGATGACGAGAGCAGTCTCGACAAACTAC

TTGCTTGGACCACCACTTTTCTCAACCACCAACTGCTCAGTAACTCCATTCTTGACAACAAGTTGCATAAGCTGGTGAAATACTACTTGAAAAACTACCA

TGGCAGAATCGATGCAGTGGGAGTTAGACTAAACCTCGACCTATATGAAATGAATCATTACAAAACTCTAAAGACTTCACATAGGTCCTCTAGTCTTAGT

CTATGTAATGAAAACTTTGTACCATTTGCAAGTGAAGCTTTTCATATTTCTCAAGCTCAATATCAGAAAGAACTTGAGAAACTGCAAAGGTGGTATGCAA

ATTGTAGGTTGGACAAGTTGAATTTTGGAAGAGATGTAGTAAGGATTTGTAGTTTTATGTCTTCAATCTTGATTGACGATCCTGAATTTTCCGATGCTCG

TCTATTCATGGCTAAACAAATGGTGCTTGTAACACGTGTCGATGATTTCTTCGATCATGGAGGCTCTCGAGAAGAAGCCTACAAAATCATCGAATTAGTA

AAAGAATGGAAAGAGAAGTCAGCTGCAGAATGTGATTCGGAGGAAGTCGAAATCCTTTTTACAGCAGTATACAAAACAGTAAATGAAATGGCAGAGAACG

CTCATGTTGAACAAGGACACAGTACTAGTACTGCCAAAGAATTTCTAGTTAAACTGTGGGTTGAAATACTATCAGCTTTCAAGATGGAATTAGATACATG

GAGTGATGGTACGGAAGTAAGCTTGGACGAGTACTTGTCGTGGTCGTGGATTTCGAATGGCTGCAAACTATTTATACTAAGCTCCATGCATTTGCTCCCT

GCAAAATTATGCGATGAAGAAATGCTTATGAGTGAAGAGTGCACTGATTTGTGTAGGCATGTTTCAATAGTTGGTCGCCTTCTCAACGATATCCACTCTT

TTGAGAAGGAGCGCGAGGAAAATACGGGAAATAGTGTGAGCATTCTTGTAGCAGCTCGGGATAATGAAGAGGAAGCTATTACAAAGATAAAAGGAATAGT

TGAATACCACAGGAGGAAATTGATGGAGATTGTGTACAAAAATGGAGGCATTTTCCCAAGAGAATGCAAAGACATATTTCTCAAGGCATGTAGGGCTGCA

TTTTACGTGTACTCGAGCAGCGATGAATTTACTTCTCCTAAACAAGTGATGGAAGATATGAAATCCCTAAGCTTCTAA

>MlongTPS57

ATGTCTCTTGCGCTCTCCTCTTTCTCACTTTGTCGGAGCTCTCGCGTTTCTTCAGCTTCTTTGGACACTGGGCATCCAAGAGCAGCACCGCCTAAAATTG

CTTCTGTTTCACCGTGGTTTGAGGAGACAAAAGGAAGGATAGCAAAATTGTTTGAGAAGAATGAAGAAATATCAATCTCAACGTACGACACAGCATGGGT

TGCTATGGTGCCTAGTCCATTCTCTAAGGCGGACCCTTGCTTCCCGGATTCTGTATTATGGTTGCTGGAGAACCAGTGCCCCGATGGCTCGTGGGCCCGT

CCGCATCACCACTCTCTCCACCGGAAAGACGTCCTCTCTTCTACCTTGGCGTCCGTCCTCGCTCTTAAGAAATGGGGACTCGGCCAACAACACATCGACA

AGGGATTGCATTTTCTAGAATTGAATTTCGGTTCGGCTACGGATGAGAGTCAGATTACACCCATGGGGTTTGAGATTATATTCCCAGCTATGCTTGATGA

AGCCAGAGCCTTATCTTTGAACCCTAGCTTGGAGCAAAGCACGTTGAATGATGTGATGAATCTGAGGGATTTGGAACTTAAAAGAATATACTCATCAAAG

GATAAGGAGGATTACGTGGCATATATTGGTGAAGGAATGGGGAAAATGCATGACTGGGAATCCTCAGTTATGAAATATCAAAGAAAGAATGGATCTCTTT

TCAACTCTCCTTCCACAACAGCTGCTGCTATTACTGCCTTACCTAATAATTCTAATTGCCTTAATTACCTCAATCTAGTGGTAAACAAGTTTGGCGGTGC

AGATATATATTCTCAGCTATGCACAGTTAATAATCTTGAAAGGTTGGGAATCAGCCGATATTTTATAACAGAAATTCGAAGCACGTTGGATCAAATATAC

AGGTGTTGGTCGCAGGGCGATGAAGAGATATTCATGGACGCCTCGACTTGTGCCTTAGCCTTCAAAATATTGCGAATGAATGGATACACTGTGACTTCAG

ATGCGATTACAAAAGTTATACAAGAGCGCTTTCCAAATACGGATGTCAACACAACTCTTGAATTATTTAGAGCATCCCAACTCATGCTACATCCTCATGA

AACAGAGCTTGAACATCAAAATTTAAAGCTTAGATCTATACTTGAACAAGAATTATCCAACGCTCCTATTAAATCATCGTCGTCTCGACTAAATTCAGAG

GTGAAGCATGCTCTCGACTTTCCCTTTTATGCGATTCTGGAGAGGATGGCAACTCGCAAAAATATAGAGCATTACAACTTTGATAATACGAGAATCCTCA

AAACTTCTTATTGTATGCCAAATTTGGGCAACAAGGATTTCTTTCTTTTTTCGGTAGAAGACTTCAACCGATGCCAAGCTATACATCGTGAAGAATTCAA

AGAATTTGAGAGATGGTTCGTAGAGCATAGGTTGGATGAAGTAGATCTTGCGATGAACAAGTTCGTATACTGTTATTTCACTGCAGCTGCAACTTTCTTT

GCTCCAGAGCTATCCCATGCACGCATGTCGTGGGCCAAGAACGCCGTTATGATCGCAATCACAGATGATTTGTATGATGTTAAAGGTTCTTACGAGGAGA

AGAAGAAACTAATCGAGTTGCTTCAAGTATGGGATGTGGATATGAGCACAGTATGCTGCTCCAAAGATGTGGAGATAATATTCTTAGCACTTAAGAGCAT

AGTTGTTGAGCTTCAACAAAAAGGTTCCAAGCTACAAGGCCGTTCCCTCACGACCCAAGGAATTACCATTTGGCTAGATTGTCTAAATGCATACATGCAA

GAAGTGGAATGGGAAGAAATCAAGTATGTACCCACATTCGACGAGTACTTGAACAACGCCTACGTGTCATTCGGCCTGGGGCCCATCCTTCAAATACCTA

TCTACCTTGTGGGGCCCAAGATCTCGGAAGAGATGGTCAACCATCCCGAGTACCATCGCCTCTTCAAATTGATGAGCACGTGCGGTCGCCTTCTCAACGA

CATCCGCGGCCACGACAGGGAAAGCCAACAGGGTAAAGTATCCGGTGTGGCGCTGTACATTGCTCAAAACGGCGGCGAGATGAGTACGGAGGCTGCCGTT

TCGGAGATCATGAGCTCCATCGACGGCCAGAGGAGAGAACTGCTCGGCCTCGTTTCCGACAACTGCAGTGTGTTTCCAAAGGCAATCAAGGAATTATATT

GGCACATGAACACCGTGAATCAGCTGTTTTACAAGAAGGATGACGGTTTCTGGTCAAAGGAGTTGACTCAAGTCGCGCACCATATTATTCAACGCCCTAT

TGTTCTTGAGGATTATGTTTAA

>MlongTPS58

ATGACCGATGCCGAAACAACTCTCGAATTATTTAGAGCATCGGAACTCATGCTGCATCCACACGAAAAAGAACTTGAAAGTCAAAATTTAAAGCTTAGAT

CTGTTCTTGAACATGAATTATCAAGCGCTTCCATCAAATCGTCTCGACTAAATGCAGAGGTGAAGCATGCTCTCGAATATCCCTTCTATGCAATTTTGGA

AAGGATGGCAACGCGCAAAAATATAGAGCATTACAACTTTGACAATACAAGGATACTCAAAACTTCATATTGTTTGCCAAATTTGGGCAACAAGGATTTC

TTTCTTTTTTCAGTAGAAGACTTTAACCGGTGTCAAGCCATGCACCGTGAAGAATTTAAAGAATTTGAGAGATGGTTTGTAGAGCACAAGTTGGATGAGT

TGGAGTTTGCAATGGATAAGTTGGTATACTGTTATTTCACTGCAGCTACAACTTTGTTTGCTCCAGAACAATCCGATGCACGCATGTCTTGGGCCAAACA

TGCTCTTATAGTCTCAGTAGCAGATGACTTGTATGATGTTAGAGGCTCTTTCGACGAAAAGAAGAAACTAATTGAGTTGCTTCACTTATGGGACGTGGAT

GTTAACACACAAGAATGCTGCTCCAATGATGTCAAGATAATATTTTCAGCAATTAAGAACTTAATCCGTGAGACTGCAGACAGAGGATCCAAGCTACAAA

ACCGCAACATTACCGGCCATATAATCGGCATCTGGCTAGATTGCTTAAATGCTTTCATGAAAGAAGTGGAGTGGGAAGAAATCAATTATGTCCCCACATT

CGACGAGTACATGAGCAACGGCTACGTGTCATATGGATTGGGGCCCATCATCCAAATACCCCTTTACCTTGTGGGGCCCAAGCTCTCAGAGGAGATGGTC

AACCACTCCGAGTATCATAGCCTCTTCAAACTCATGAGTACAACCGGCCGTCTTATCAATGATGTCCGTGGCGATGAGAGGGAAAGGCAAGATGGTAAAG

TATCTGGGGTGGCACTGTACATTGCTCAAAATGGAGGTGGGGAAATAATGAGTACGGAAGCTGCGGTTGCGGAGATGATGAGCTCCATGGAAACCCACAG

GAGAGAATTGCTGAAGCTGGTTTTGGAGGAGAATTACGGTTCTGCCCTTCTTCCCAAGCCATGCAAGGAATTGTTTTGGCACATGAGCGTGGTGAACCAG

TTGTTTTACAAGAAAGGTGACGGTTATTATTCAAAGGAGTTGACTCATGTAGCGCGGGAAATTATTCAACACCCTATTATTGTTCTTGATGAGGAGGAAG

TTAATTAG

>MlongTPS59

ATGGAGCCCTCACTTTCCTCAATACACTCCCTTGCAACCGAGGTTAAAACCGAGATTTTCTCCGTCCTCCTCGACAACAATAATATCGAAGCATTCGTCT

CCACATCAGCCTACGACACAGCATGGTTAGCCATGATTCCCCTCGAAAAAGAAAGTAATGAAAAGATTCCCATGTTCTCGAGTTGTCTGAGCTGGATACT

CCGAAACCAGAAAGATTCCGGATTCTGGGGAGAATCCGACGATCAAGATTTGCCCACCATTGATGCTCTCCCCTCTACTCTCGCTTGCTTGGTTGCCCTC

AAAATGTGGAATGTCGGCCATCAGAATATCGAATCAGGGCTGAGGTTTATCAACTCGGAGGCGGAGATGGTTATGAGAATGAACTACCGGAAGCTGCCAC

GGTGGTTTGTGCTGACTTTTCCGGCGATGGTTGAGCTGGCGGAGGCGGCGGGGCTTCACGTTGTTTTCCCACCGGGATTAAACGCCGCCGTAGCTCACAT

TTTCGTCATGCGCCACCATATTCTTCAAAAGGAGGGGCTAGAAGTAGAAGGTGATTACGAATCGCGGTGTTTCCCGCCACTTTTATCGTACTTGGAGACG

CTGCCGTCAAGTTATGGGTTTGACCGTGAAGAGACCGTTAAGAAGTACTTGAGCAGCGACGGCTCTCTGTTTCAGTCGCCGTCAGCCACCGCTCAAGCCT

TCATATCCACCTCCAATGTCCACTCTCTCAAATATCTGCAGTCCCTCCTTCACAAATTCCCCAATGGAGGCGTACCTGCGAAATATCCAGTGGATGGAGA

GTTGATGAAACTGTACATGGTGGATCACGTGCAAGGACTTGGTCTAGCTTCCTATTTCAATCAAGAAATTGACCAACTACTTTCTCAACTTTACAACAGT

GAAAAATCAAATCCGAGCGCCTCCCTCCCCTTCAAATTATTTAAAGACGCATTGGCTTTTCGCCTTCTTCGGATGCGGGGCCACCGCGTAGATCCAGGAA

GCTTCTGCTGGTTTGTGCACGAACCCGAAACGATGTCGTACATGGAGGAAAACTGCGAAGAGTTCACCAGCGCAATGTACAGTGTATACAGAGCCACAGA

TTTATCCTTCCCAGGGGAGAAGGAAATGGAAGAAGCGAGAAATTTTGCTATAAAACTGCTCCAAAATACCAATACCAACACCATGCACGCAGATCATAAT

TTACTCATTACAAAAGGACTTCAAAATATGATAAAGTATGAGGTTGATGTTCCGTGGTATGCTCGACTTGATCGCCTCTATCATAGAAAGTGGATTGAAG

AAAATAAAACTAGCACTTTATTGCTTGGGAAGGCCTCTTTCTATAGGTTATCCTGCCTAGACAACGCGAAGCTGATGCGAGTGGCGGTTGAAAATTTCGA

GTTCATGCAGTCTGTATACGTACGTGAATTGGAGGAGCTCAAAGGGTGGTCGAAGAAATGGAGGCTAAGTGAGATGGGATTTGGGAGAGAGAAGACAGTG

TACACGTATTTTGCAGTAGCGTCTTGCTCTCTCTTTCCCCACAATTCAATTATGCGTTTGCTCATTGCAAAGGCAGCCATTATTGTTACCGTGGCCGATG

ATTTCTACGATATGGAAGCTCCTTTGCCTGATTTGGAAATCCTCACTCTTGCGGTTCAAAGATGGGAGGGTGAAGGATTAGAGGGTCATAGCAAGACTAT

CTTCTTCGCCCTTGATGACTTGGTGAAACACATCGTTGCTAAATGCTACCCTCTGCAAGGATCTCAAGTTTTGCCCAAGTTGCAGAATCTATGGCGGGAA

TGCTTCGAGTCGTGGATGGTGGAGAGAAGATGGAGCTCGACGGAGTACAGACCGGCCATGGACGAATACCTCGAAAGGGGAATGGCGTCGGTCGCGGCAC

ACGCCATCGCTCTTCCGGCAACCGGTTTCTTGAACCAGAACGGAGGTTTCGAGTACCAGAACATCACCAAATTGCTCATGGCCGTCGCCCGTTTAGCAAA

CGACGCTCAGAGCTATGAAAAAGAAGTGGTGGATGGGAAGATGAACATGGTGATGCTCCACTCCCACGAGAATCCGGGCGCCAGCATCCAAGAATCGGAG

GCCTACGTGAAGGCGATACTCGAGCTGAAACGGAAAGAATTTCTGCAGCACGTCTTCACGGGCGAAGAAATGCCCACATCGTGCAAGCAGACACACCTCT

ACTGCATGAAAGTGTTTGAGATGTTCTACAACTCTGCAAACTTGTTCGACAGCGAAACTGCCCTCGTCGAAGACGTGAAGAAGTCCATTTACCTTCCCAT

TCGTCGCGCCAAAACCTCGAAGCCCGTCCCCTCCCTCGACAAACCCAAGGTTTCTGCTGTCTTTAAGTTCCCTGCAATTCCTAGAACTCCAAACACCACC

AGTTTTATCAAGAATTCCTCTGTTTCGGGCCAACCAAAAGCCCGGCTTCCCCTTCGACTTAATCTAAGCTTTGCATAA

>MlongTPS60

ATGCAGGAGAAAAAAACAGAGCAAGTAAGAGATTTGTTGGTGAAAGTGAGGCAAGATTCTTTGGAAAGCTTGAAACTCGTGGATTCCATCCAACGCCTTG

GCATTTGCTATCTCTTCAAAGATGAGATCGATTCCATTCTCCATCACCAATATTCGGCTAAAATGGGAAATGACCATGATGCCCTTTTCGAGGCTTCGCT

TCGTTTCTGTCTGCTCAGGCAACATGGATACGAAGTCGTCGCAGATGACTTCTTCGGGCATTTTCTACGTTGCGAAGGTGGCCTAGTTGGTGAAACGAGA

GACGTGATGGCGGTGCACGAAGCATCCCATTTAAACATGGGAGGGGAAGAAATTTTGTATGAAGCTGCAGTATTTAGCTCTAGCTATCTCAAGAAAAAGG

CCAGCATTTGCCTTGATGACAATGTCGTGGAGGCGAAAATGGCGAAGCATTGCTTGGTTTATCCCCAGCACAAGAATCTGGCTGGGTTTACTGAGAAAAG

TTATCTTCAGTTTCTGAATGGAGAAAACCCTCTCTTGGGAGAGCTTGCAGAATTCGAATTTCGAAACCTCCAGTCCTTGCACAAACTCGAAATTCTTCGC

GTTCTCGAGTGGTGGAAAGGGGTGGGGCTGAATTCCTCAACCTCAAGGAATAAGCCAATAAAATGGCACATGTGGTCCATGGCAACTCTGAGGAATCCTA

GTTGGTCCAAACATAGGATTCTGCTAACAAAGCCAATTTCTCTTGTTTACGTCGTCGATGATATATTTGATCTTAACGGTACAATTCTTCAAGACCTCAT

TCTCTTCACTCAAGCCGTTAATAGATGGGATGCTTCTGCAATTGATGAGCTACCAAGTTACATGAAAGCATGCTTTGATGCAATCTATGAAACCACCAAT

GAAATCAGCAGCGTTGTCCGTAGGGAGCATGGATGGGACCCTATCCACTTCCTCATAAAAGAGTGGGGGAAATTGATGGATGCATTTCTTGTGGAGGCAA

AATGGTTTAGAAGTGGAGAATTGGCAAAGGCAGAAGAATATCTCAAGAATGGAGTGATGAGCTCGGGTGTGCCCATGGTGCTATCTCATCTCTTCTTCCT

TATGGGAAACCCTCTCACTAATCAAACCCACATTCTACTCAATGATCCTCATGGACTCACTCATTCCGTCGCCAAGCTCCTCCGACTCCTCGATGATTTG

GGCTCCGCCCAGGACGAGCAACAAGAAGGGTACGATGGATCGTACATAGATTGTTACATGAAAGAAGGGAAAGTGGAGTCACGTGAAGCTGCAAGAGAGC

ACGTGATGTGCATGGTTTCAGACACGTGGGAAAACATCAACAAGCACGGCCTCTCTTCCGCGGCTCCCCTCTCTGCTTCTTTCCGATCGGCTTGTCTCAA

CGCAGCGCGTATGGTTCCGACCATGTATACCTACGACAGAGATCATCGTCTCCCTCTTCTCCACCGCCATGTCAGCTCCATGTTTCGCGACCCCACCTTC

GCCAAATCAATCGCGGAATAG

>MlongTPS61

ATGGCAGTCTGTAATTTCTTGTCAACTTATGGTTCCCATCCCAGGTTTAGAAGAATAATTAATCCAAAGAGGAACAGCTTTGGAGCAACAAGCTGCTGTA

CAACAAGTCGTCAGAAATGGAAAATCTCAAAAGATCTTGGATCCCATTTGAAGGGAAAGGGATACCACTTCTCGGAGTACTTTTTGGAAGACTACAAGAA

AAAAATGGGAGAGATGAAGGAACTCTTATTGCAACATATAAATCTTGATCAAGCTTTGATTTTAGTGGATGCGATTCAAAGGGTAGGAATGGAACACCAT

TTTGATGAGGAGATTGAGATGATACTTGAACGACTCTATTATTCAACCAACAAATCTCCTTACATTCATCATGATTTATATCACGCTTCTTTGCATTTTC

GATTACTCAGAAATCACGGATACTATGTTTCACAAGATACGTTCAGCAGTTTCAAGGGAAATGATGGGAGGTTCAAAGAAAAGTTAAGGCAAGATATTAG

AGGATTGATGGAATTGCATGAAGCATCCCAACTAAGGTTCAGCGGTGAAGATACAATTGATGAAGCAGAAGAATTCAGCAGAATGAATCTGAATAAATTC

TTGGAAAATATCAAGGATGATGATGATTGGCATTATAAAAAAGTGATAAGGAATTCAATGACACGGACTCAACACATGAACATAGCAAGATTAACAGCAA

GAAATTACATGGATGCAGGTTTCCTTAAAGGTTCTAAAAAGGGATGGGAAAAAACATTATCCGAGCTGGCAAAAATGGATTTAATGATTGGGGACATGTT

GCATCAAGAGGAATTACTAGAGATTTCTAAGTGGTGGGAAAACATTGGCATAGCTAAGAAATTAGACCGTGCAAGAAACCAACCGGTGAAGTGGTACACA

TGGTCTATGGCCATCCTCATCAACAATCCAAGCTTGCGACTTGAAAGAATAGAGCTAACCAAGGCCATTGCATTTATCTACCTGCTCGATGATATCTTCG

ATCTATATGGAACAATAGATGAACTTACCCTCTTCATGCAAGCCCTAAATAGATGGGAGTATGGTGCAAGTGATACATTGCCAGAATACATGAGAAAGAG

TTACAAAGCCCTTCTCGATACCACCAACAGTACAGCCCAAAAGGTTGAGGAAAAGTACGGTACTAATCCTATCGAATCTCTTAAGGACACGTGGGGAAGT

CTGTGCAGGGCATTCCTAGTAGAAGCGCAATGGTTTAGATCAGAAAAGTTGCCTAGCGCAAAAGAGTATTTGGAAAATGGTAAAGACGAGCACCAAGATG

GTTTGGATGGTTCATACATAAAATGCTGCATGTACGATGAACCAAATCTGTCGGAGAAGGAAGCACGACAACATGTGGAGGAGATGATGTCAAACCAATG

GAAACACCTAAATGGGGAATGTTTTCTACGCCTGAATCGTCCCTCGGTCTCAGGTCTCAGAAGGGCTTCTCTGAATGTTGCAAGAATGATACCTCTCATG

TACACCTATGATAAAAATCAGAGGCTTCCGCTCCTTGAGGAGCATATCAACACTTCTAAATTGTTCATCTGA

>MlongTPS62

ATGGCAACATTATGCAATAGCTTCTCGCCTATTTTCATAAAATCTTCTTCTTCTTCAGTTATAGTTTTATTTAACGAAGTCAAATATTTTGGAAATACTA

ATAAACAAGCTGTTTGGGTGGCTAAGAAGCAGTATCAGTGTTATTCTGTATCAACTTCTGCACCTCAATTTGATCACAACGACAACATTTTCCAGGATAC

TTTTCTAAATCACTACCAAGAGAAATTGGAGAACATTAGGGGCTTACTTGAGTCCAAGGAAGAAGAAGAAGAAGAAGATCCAGTAGAAAGTTTGATATTT

GTTGACACCATCCAACGGCTAGGAGTCGCCGCTCATTACCAGAAGGAGATCCACAGTATTATGCATCAGCACCACCATTATTTCAATAATAATAAATGTG

GGTACGAAAAGCAAAGGCTCCATGATGTTGCCCTTTCATTCCGACTCTTGAGACAGAGAGGATACCATGTTTCTCCTCAAGATGTGTTCAAGAGTTTCAA

GGGCAGAGATGGGAAGTTCAGAGGCGATCTAAAGCAAGATATTTGGGGATTGTTGGAGTTATACGAAGCTACACATATGAGTTTTGAAGGAGAAAGTGTA

CTGGATGAAGCACAAGATTTTTGCAGGCAAGCGCTATTAGCAAACAAGTGTGAGGAAGAAGTTGTTGAGATGAGGCTGAGGCTGAGGCACCCTTTTCACA

AAACCATTGCAAAATTCACACAAAAACACTTCCAATTCCAAAGAGATGATAATATTAATGTTGGAGTTGGAGGCATCAGTACAACTAATTTAACAGAACT

GTCGTTACTCGATTCTCTCTGGGGAAAACACGTGCACCGCCAAGAACTCCTCCAACTTTCCAAGTGGTGGAATGATTTAGGGTTAAACACAGAGCTAAAG

CTAGCCAGGAATCAACCGGTGAAATGGTACACGTGGTCAATGGCCATCCTCGTCGATGATATTAGCTTATCGGAGCAGAGAGTAGATCTCACCAAGTCCA

TCGCTTTTATTTACTTGATTGATGATCTTTTTGACCTTTATGGGACTCTCAATGACCTGGCTATCTTTACTGAAGCAGTCAAAAAATGGGAGTACGGTGC

TATGGAAATGCTACCCGACTACATGAAGTTGTGTTACAAATCCCTCCTTGATACCACACACCAAATTGGGCTCACCATTTCTCTAAACCATGGACACAAT

CCTATCAACTCTCTTAAACAAACGTGGGCAAGTTTGTGCAATGCGTTTTTAGTGGAAGCGAAGTGGTTGAGGTCGAAGAATTTGCCAGGGGCAAAAGAGT

ATTTAACCAACGGAAAGGTGAGCTCCGGGGTGTATGTGGTGCTAGTCCACTTATTCTTTCTTTTGGGTTACAGCGGAACCAATTTAGATGACGTGTCCCC

ACTCATTTCATCTGTTGCTACAATTCTTCGCCTTTGGGATGACTTGGGGACTGCCCAGGATGAACACCAGAATGGTAGCGATGGATCTTACATCGAATGC

TACATGAAAGATCACCCAAACTCAGAATTCGAGCACGCACGGGAGCACGTGGTGGATACGATAGAAGGTGAATGGAAACGGCTCAACAAAGAGTGTTCAT

CTGAATCACTCTCGAGAGCTGCTCTTAATCTGGCGAGAATGGTTCCTCTCATGTACAGTTACGATGGCAATCAACGACTCCCTGAGCTTGAAAAGCATGT

TCAACTAATGTTGAATCAGCTCATTTGA

>MlongTPS63

ATGTCTTGTGCAAGGAGCGCCGTGTCATTGTTGTCGCAATCCTCGTATCGCTTCCCAAGGACCTCAATTCCATCGCGAACAAGGGTTTGCCCCGCCGCCG

CGGTACCCCTCCGCCGCTTCTCCACCTGCAAGTCGGCGGCTACGGCGCCAGCGCCACCAACTTCTTTGCATCCCATCATCAACGGCAACGGCAATGGCCC

TCTGCTTGAAACTCAGCTGTTTCCTTGGGAGGAAACGAAGAGGAGTACTGAATATTTACTGGAGAAGACCGCAGCGAAACTCCAGAGCACAAGCATGGAA

GCATCGGAGAAACTCAAACTCATCGACGAGATCCAACGGCTAGGAATCGGCCACCACTTCGAAGATGCGATCGATAGCGTACTACAGGTCCAGTGCTCCG

CTTTCTCCAAAGACGAAGACCTCTTCACCACGGCCCTGCGCTTCCGCCTCCTCCGCCACGCTGGCTTCCACGTCACCCCCGAGGTGTTGTTGAGATTCAA

GGGCAAAAATGGAAAGTTCGAAGAGTCCTTGAACGGGGACACGATAGGGTTACTGAGTTTGTACGAGGCGTCGAATATGGGGGCTCAAGGCGAAGAAATA

TTGGAGGAAGCAATGGAGTTCGCCGAGTCTCGCCTCCGGCGGTCGCTTGCTGAGCTGGCACCGAGCCTGCGTGGCGAGGTGGGCCAGGCCCTAGATGTCC

CGAGGCACCTGAGGATGGGTAGGTTGGAAGCAAGGCGGTTCATCGAGGAGTACGGTGAGCGAAGCGAGCACGACGGAGACGTTCTGGAGCTAGCAATTTT

GGATTATAACCAAGTCCAGCATCAGCACCAAACGGAACTCACGGAAATTACAAGGTGGTGGAAGGAATTAGGTTTGGTGGAGAAATTGAGTTTTGCGAGA

GATAGACCGTTGGAGTGCTTTTTGTGGATGGTGGGGCTTCTTCCGGAGCCAAAGTACTCAAGCTGCAGAATAGAGGGGGCGAAGACCGTCGCTATTCTGT

TGGTGATCGACGATATTTTCGACACCTACGGCAAGATGGATGAACTCGTTCTCTTCACCCATGCAATTCGAAGATGGGATCTTGAAGCAATGGAAACCCT

GCCCGAGTACATGAAAATATGCTACATGGCTTTGTACAACACCACCAACGAAATATGCTACAAAGTCCTCAAGGACACCGGACGAACTGTCCTCCCATAT

CTCAAATCTACGTGGGTAGACATGATTGAAGGTTTCATGGTGGAGGCAGAGTGGTTCAACGGCGGAAGTGCACCAAATTTGGAAGAGTACATAGAGAATG

GAGTGTCTACGGCGGGTGCATACATGGCTTTGGTGCACCTCTTCTTTCTAATTGGAGAAGGCGTCACCGACCAAAATGCTTCGCTTTTGACCCAAAAACC

CTATCCCAAGCTCTTCTCCGCCGGCGGCCGGATTCTTCGCCTATGGGATGATCTCGGAACCGCCAAGGAGGAGGAAGAGCGTGGCGATCTGGCGTCGAGC

ATACACTTAGTTATGAAGGAGTACAACCTGTCGACGGAAGAGGAAGCTAGAAGTCGCATTTCACAAGATATTTTCCGGCTATGGAAGGATCTCAACGGAG

CGCTCATCTCCAACAAAAACGTGTTGCCACCATCCATAATTAAAGTCGCACTTAACATGGCGCGAGCTTCCCAAGTTGTGTACAAGCACGAACAAGACAC

CTATCTTTCAAGCGTCGACAATTATGTGGAAGCCCTCTTCTTCACTCCTATACTTTCATCTTAA
